# Thematic Mapping and Evolution of Social Media Mining in Health Research: Hybrid Bibliometric Synthesis

**DOI:** 10.2196/86200

**Published:** 2026-05-08

**Authors:** Mia Jiming Yang, Sabine Bohnet-Joschko

**Affiliations:** 1 Chair of Management and Innovation in Healthcare, Faculty of Management, Economics and Society, Witten/Herdecke University, Alfred-Herrhausen-Str. 50, Witten, North Rhine-Westphalia, 58455, Germany, 49 2302-926-38043

**Keywords:** social media mining, bibliometrics, machine learning, strategic mapping, health research

## Abstract

**Background:**

Social media platforms offer extensive data, as they are widely used globally. Social media mining (SMM) enables real-time monitoring of user-reported health information and serves as a supplement to traditional health data analytics. However, the rapid proliferation of literature has produced fragmentation, and a comprehensive knowledge map regarding SMM is lacking. Further, existing bibliometric reviews in health fields are easily undermined by synonym fragmentation and parameter settings, reducing their robustness. Thus, a more robust, reproducible, and decision-oriented bibliometric framework is required.

**Objective:**

This study aimed to (1) outline key thematic clusters in health-related SMM and map their dynamic evolution, and (2) methodologically demonstrate how machine learning–based bibliometric analysis can strengthen the robustness, transparency, and foresight capacity of evidence synthesis.

**Methods:**

This study designed a fully automated and reproducible bibliometric analysis of PubMed journal articles published from 2015 to 2025 (n=250) and analyzed records with both abstracts and keywords (n=189). We performed cleaning and standardization for titles, abstracts, author keywords, and MeSH terms, and carried out an exploratory descriptive analysis to obtain preliminary insights into publication patterns. Subsequently, we used SPECTER2 and PubMedBERT embeddings with keywords and abstracts to construct a hybrid similarity matrix. Then, we applied Uniform Manifold Approximation and Projection for dimensionality reduction, followed by Hierarchical Density-Based Spatial Clustering of Applications with Noise for thematic clustering, and visualized the results in a 3D strategic coordinate system (maturity, influence, and recency). We performed intercluster relationship analysis and time-slice analysis to examine thematic intersections and evolution. To ensure robustness and enhance interpretability, we implemented dual-level validation.

**Results:**

We identified 6 thematic clusters: cluster 1 (candidate incubator pool of peripheral cross-cutting topics in health-related SMM), cluster 2 (computational methods in health informatics), cluster 3 (public attitudes and sociopsychological determinants), cluster 4 (infodemiology and the COVID-19 information ecosystem), cluster 5 (health communication and public health engagement), and cluster 6 (social media analysis and network methods). Strategic 3D mapping revealed that methodological clusters (clusters 2 and 6) occupied high-maturity and high-influence positions, while application-driven clusters (clusters 3 and 4) occupied high-influence and high-recency positions, representing rapidly expanding frontiers. Clusters 1 and 5 demonstrated strong potential for further growth. Temporal slicing confirmed a trajectory moving from methodological consolidation and thematic diversification to a renewed focus on convergence and problem-solving. Validation showed strong semantic coherence and robustness of the methods and findings.

**Conclusions:**

We developed a semantic-structural hybrid bibliometric framework with dual-level validation, reducing synonym fragmentation and parameter sensitivity inherent in traditional approaches. The resulting decision-oriented knowledge map offers strategic guidance for infodemiology-informed and audience-segmented public health communication, research priority settings, and the deployment and evaluation of real-world surveillance and pharmacovigilance workflows while supporting evidence-driven and patient-centered decision-making in public health and health care.

## Introduction

Social media has become a ubiquitous component of modern life, with over 5 billion people worldwide using social platforms as of February 2025 [1]. The value of social media data in health research lies in its volume, velocity, and variety, and the insights derived from user-reported content. Unlike traditional historical data, social media offers a large-scale, real-time representation of the natural state of public discourse and behavior. Content from platforms, such as Twitter and Reddit, constitutes a rich data source for gaining insights into population health trends, behaviors, and opinions [2-4]. Researchers have recognized that the computational analysis of social media data—often referred to as social media mining (SMM)—enables real-time health monitoring and health-related interactions that are unattainable through traditional data sources [5]. In fact, SMM is flourishing as an emerging science aimed at providing health care stakeholders with additional evidence for decision-making.

Over the past 5 years, there has been explosive growth in digital health research utilizing social media data, spanning areas such as epidemiological studies and pharmacovigilance [5-8]. Moreover, SMM techniques have been applied in health communication and promotion (eg, evaluating public responses to health campaigns), mental health monitoring (eg, identifying depression-related discourse online), and behavioral research (eg, analyzing lifestyle-related topics) [2,9,10]. Mapping such a fragmented and rapidly evolving field has become especially important. Therefore, a literature review in this area can provide an overview of existing research.

Four reviews have previously addressed the use of social media and the analysis of social media data for health purposes. A review of the application of SMM in health outcomes included 19 papers [11]. However, it was published in 2019, prior to the major expansion of social media data mining, and only provided an overview of literature related to medication and treatment side effects. The authors suggested at the time that adverse drug events and health-related quality of life would be future research directions. Another review on social text mining conducted in the early stages of the pandemic focused on the lack of ethical consensus and guidelines in this emerging field, thus emphasizing ethical standards in the use of social media text data analysis [12]. Subsequently, a 2021 review of the literature on social media for health purposes identified at least 10 applications in the health domain—spanning health interventions, public communication, surveillance, education, and research—highlighting the breadth of the field [13]. While this review did not provide an overview of SMM, it illustrated the diverse potential application scenarios of SMM. Another scoping review on the analysis of social media data in health care searched 5 academic databases, including PubMed, Web of Science, and Embase, and ultimately included 134 papers on social media data analysis [14]. However, only 55 of these papers fell under the category of SMM, with the rest being traditional qualitative studies. Although this review only briefly described the methods used in SMM in the section on computational tools, it pointed out that future analysis of social media data will inevitably move in a technology-driven direction.

In terms of research methodology, existing bibliometric reviews on health research and medical innovation predominantly follow 2 typical approaches. The first approach uses co-word networks and thematic mapping based on keyword co-occurrence, combined with descriptive metrics, such as publication volume, country- and institution-level collaboration, and core journals, to summarize the research landscape [15-22]. The second approach uses topic modeling techniques, such as latent Dirichlet allocation (LDA) and structured topic modeling, to extract latent themes from textual data [15,21,23-28]. While valuable, these methods often exhibit 2 limitations when applied to rapidly evolving research fields. First, pure co-occurrence statistics rely excessively on string sequences and frequency counts, frequently overlooking synonymy, near-synonymy, and conceptual fragmentation caused by variations in expression, and the results are highly sensitive to threshold selection [24,29-31]. Furthermore, traditional topic models typically rely on the bag-of-words assumption and require predefined topic counts, potentially compromising comparability and robustness across different time windows [23,24,29]. Addressing these methodological gaps, this study proposes a hybrid bibliometric framework integrating semantic and structural analysis [30]. We introduce semantic similarity while preserving the co-occurrence network structure to mitigate synonym fragmentation [30]. Aligned with this approach, a recent study has integrated structural and semantic information within citation networks to more precisely characterize the influence of publications and their capacity for interdisciplinary knowledge diffusion [32]. Density-based clustering enables topic cluster identification without prespecifying the cluster numbers [33]. By integrating time slicing, burst detection, and multidimensional strategic coordinates (maturity, influence, and recency), we systematically characterize a topic’s knowledge accumulation, real-world impact, and recent growth potential. This provides a more decision-oriented, reproducible evidentiary landscape for the field. To ensure the robustness and reproducibility of this hybrid framework, we further detail the rationale for key parameter settings in the Methods section and demonstrate the validity and stability of the methodology and results through multiple validation tests.

In summary, prior studies have involved highly diverse application scenarios of social media data and have made fragmented statements. While previous reviews have cataloged applications and thematic summarization, they have rarely integrated advanced computational approaches for a structured synthesis. In particular, bibliometric reviews often rely on co-occurrence statistics or topic models with limited robustness. This leads to a limitation in sustaining a prospective strategic vision. To address this, our study developed an automated, reproducible pipeline for bibliometric analysis of SMM in health care, introducing several methodological innovations.

Furthermore, this study aimed to not only identify hot topics but also trace their temporal evolution. Additionally, this study attempted to explore the future hotspots of research innovation and reveal the composition of the innovation incubation pool, thereby helping researchers, funding agencies, and policymakers to anticipate future directions and foster interdisciplinary integration. Thus, this study had the following questions:

1. What are the main topics of research on SMM in health care and their latent patterns?
2. What are their developmental stages, their strategic positioning, and the future potential research themes?

To answer these questions, we used the following guiding goals: (1) substantively outline key thematic clusters in health-related SMM, map their pre-, during-, and postexplosive growth period evolution, and evaluate the implications for fostering social equity, effective health management, and informed policy, and (2) methodologically demonstrate how advanced bibliometric methods can strengthen the robustness, transparency, and foresight capacity of evidence synthesis.

## Methods

### Study Design

This study aimed to systematically review and analyze the research progress and thematic evolution of SMM in the context of health by constructing a fully automated and reproducible bibliometric analysis framework. To achieve this objective, we designed a multistage methodological pathway in accordance with BIBLIO (Preliminary Guideline for Reporting Bibliometric Reviews of the Biomedical Literature) [34]. Figure S1 in Multimedia Appendix 1 illustrates the research process. The methods are elaborated in more detail in Figure S2 in Multimedia Appendix 1. Additionally, the BIBLIO checklist has been provided in Checklist 1.

### Data Retrieval and Processing

The first phase of this study involved constructing a comprehensive literature dataset. We selected the PubMed database as the primary data source, as it is one of the most extensive and authoritative bibliographic databases in the field of health research. We summarized the search strategy, record identification, and inclusion process using an adapted PRISMA (Preferred Reporting Items for Systematic Reviews and Meta-Analyses) 2020 flow diagram. Following the PRISMA-S extension for reporting literature searches, we have presented the search process in Checklist 2 to strengthen the transparency and reproducibility of the search [35]. To ensure the comprehensiveness of the search, we used a search strategy utilizing Boolean logic operators to combine free-text terms and Medical Subject Headings (MeSH) terms. The query included terms for social media platforms (eg, “social media,” “Twitter,” and “Reddit”), combined with terms related to data mining and analysis (eg, “mining” and “data mining”). We also applied filters to include only journal articles from January 01, 2015, to July 31, 2025 (excluding news and retracted articles). The literature download process was automated using the Entrez module. Necessary parameters, including *Entrez.email* and *Entrez.api_key,* were configured to comply with National Institutes of Health application programming interface (API) access guidelines and prevent connection errors during high-frequency requests. All retrieved results were stored to ensure transparency and traceability throughout the research process. Furthermore, to mitigate data loss due to API updates or network instability, we implemented comprehensive logging after each batch download and recorded completion timestamps, the search strategy used, the number of records returned, and other relevant metadata, thereby guaranteeing data integrity and reproducibility.

In addition to the metadata obtained from PubMed, this study integrated supplementary metrics from external databases and official journal websites. Specifically, we collected the H-index of relevant journals from the SCImago database to reflect their academic prestige and long-term impact. Concurrently, the most recently released impact factor (IF) and 5-year IF were acquired from official journal websites and matched with the publication records. Then, we retrieved the relative citation ratio (RCR) of the included articles, which is a normalized measure of citation influence across fields and time. The RCR was used only for post-hoc external comparison and was not included in embedding, clustering, parameter selection, or labeling. The integration of these supplementary data laid a solid foundation for subsequent external validity checks.

During data processing, we extracted the core metadata, including title, keywords, abstract, publication year, journal, etc. Then, we performed keyword standardization by unifying plurals and synonyms, expanding abbreviations, and mapping entry terms to MeSH descriptors with the NLM MeSH descriptor dataset (accessed on July 31, 2025; latest record revision date: July 15, 2025).

### Exploratory Descriptive Analysis

After processing the bibliographic dataset, we conducted an exploratory descriptive analysis to characterize SMM in health research. This stage provided a phenomenon-level overview to guide subsequent structural modeling. We explored annual publication trends with autoregressive integrated moving average (ARIMA)-based exploratory projection, collaboration networks between institutions and researchers, countries, citation impact, and keyword dynamics (keyword statistics and Kleinberg burst detection). Overall, this multifaceted descriptive analysis established an overview of the research. The methods of keyword burst detection and parameter robustness assessment are described in Multimedia Appendix 2.

### Thematic Clustering and Strategic Mapping

This study proposes an automated bibliometric pipeline specifically designed for literature on SMM in the health domain. Although we performed normalization, residual terminological variations may still exist. Therefore, we developed an automated process integrating structural and semantic similarity, where semantic proximity is derived from contextual embeddings (PubMedBERT and SPECTER2) computed from titles and abstracts to help reduce fragmentation caused by near-synonymous or variably expressed terms and to capture contextual semantics. A hybrid similarity matrix was reduced with Uniform Manifold Approximation and Projection (UMAP) and clustered via Hierarchical Density-Based Spatial Clustering of Applications with Noise (HDBSCAN) under multiple parameter profiles. Outputs included interpretable topic clusters with representative keywords and their positions in a 3D strategic diagram (maturity, influence, and recency). In this study, the number of papers published for a keyword represents maturity, the number of connections a keyword has in co-occurrence networks represents influence, and the proportion of papers published within the last 5 years represents recency. Details of parameter tuning, thresholds, and ablation tests are provided in Multimedia Appendix 3. After clustering, we performed an intercluster relationship analysis on the weighted keyword co-occurrence network to quantify cross-theme connectivity. We computed a cluster-to-cluster coupling metric based on the total cross-cluster co-occurrence strength, ranked cluster pairs to identify the most strongly connected themes, and interpreted these links (information is provided in the Results section). To explain what drives these cross-cluster connections, we further identified pair-specific bridging keywords contributing the most to the coupling between each highly coupled cluster pair.

### Dynamic Temporal Slicing

For the dynamic analysis, the study period was divided into 3 slices with the cutoff of explosive growth: 2015‐2019, 2020‐2023, and 2024‐2025. Within each slice, we performed spectral clustering on the keyword co-occurrence network. This allowed us to observe the emergence, growth, and decline of research themes over time. Details of the process are provided in Multimedia Appendix 3.

### Validation

This study conducted either macro-level or micro-level validation. To assess the robustness of our methods, we conducted internal validation through a series of sensitivity analyses. Moreover, we validated the findings with external checks using RCR alignment.

In addition, we conducted micro-level interpretive triangulation through evidence mapping and expert content analysis. The 3D strategic diagram was divided into 8 quadrants based on a reference point (x=0, y=3.51, z=0.5). From each cluster in each quadrant, we sampled 1 representative keyword that was the farthest from the reference point. For each sampled keyword, we retrieved 2 full-text articles: 1 with the highest RCR and 1 published in a journal with the highest IF. Two reviewers examined the full texts to extract evidence on research introduction, methods, social media sites, contributions, challenges, and future outlook. The protocols for the analysis of the selected articles are described in Multimedia Appendix 4. This interpretive triangulation was performed after the mathematical analyses were finalized and was not used to fine-tune parameters, select models, modify cluster assignments, or remove records and keywords.

### Tools and Software Utilized

All analyses were conducted primarily in Python (via PyCharm IDE), with the exception of burst detection, which was implemented in RStudio (Posit). Microsoft Excel was used for supplementary manual inspection and verification of selected outputs, and Microsoft Office Visio and Draw.io were used to draw flow charts.

### Ethical Considerations

This study was a bibliometric and secondary analysis of bibliographic data about academic publications from an open-access database. Neither humans nor animals were involved in this work. Therefore, ethical approval was not required. Moreover, the original data for this study consisted entirely of publicly available bibliographic information. During the research process, we did not collect any individual-level participant data or recruit human participants. Therefore, informed consent was not required, and no compensation was provided. Furthermore, the study exclusively processed publicly available article-level metadata and reported the results in summary form. None of the data collected, processed, or included in the manuscript text and supplementary materials contain any personally identifiable information. Consequently, there is no risk of individual privacy disclosure, and no additional authorization or consent documents are required.

## Results

### Publications Identified

**Figure 1.**
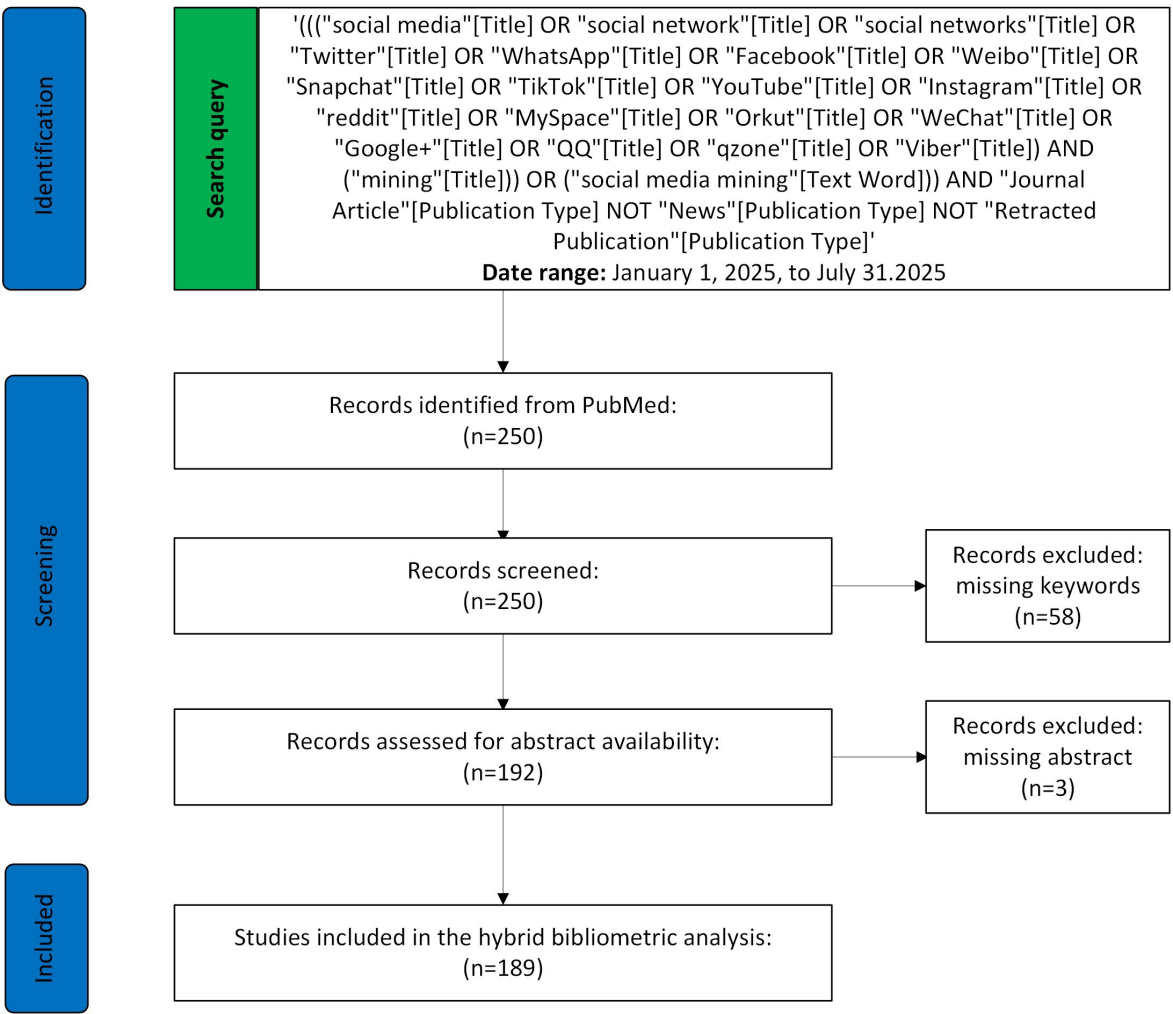
Adapted PRISMA (Preferred Reporting Items for Systematic Reviews and Meta-Analyses) flow diagram for bibliometric dataset construction using PubMed. Exclusions were based on objective metadata completeness (missing author keywords/abstract), without manual title/abstract screening or full-text eligibility assessment. The image has been reproduced from Page et al [36], which is published under Creative Commons Attribution 4.0 International License [37].

In the initial stage, we retrieved 250 publications from PubMed covering the period between January 1, 2015, and July 31, 2025. After the exclusion of articles without an abstract or author-supplied keywords, a total of 189 publications were retained for systematic analysis (Multimedia Appendix 5). The PRISMA flow diagram is presented in Figure 1. Moreover, details of the search strategy and variations of the key search terms are presented in Figure 1.

### Exploratory Descriptive Analysis

**Figure 2.**
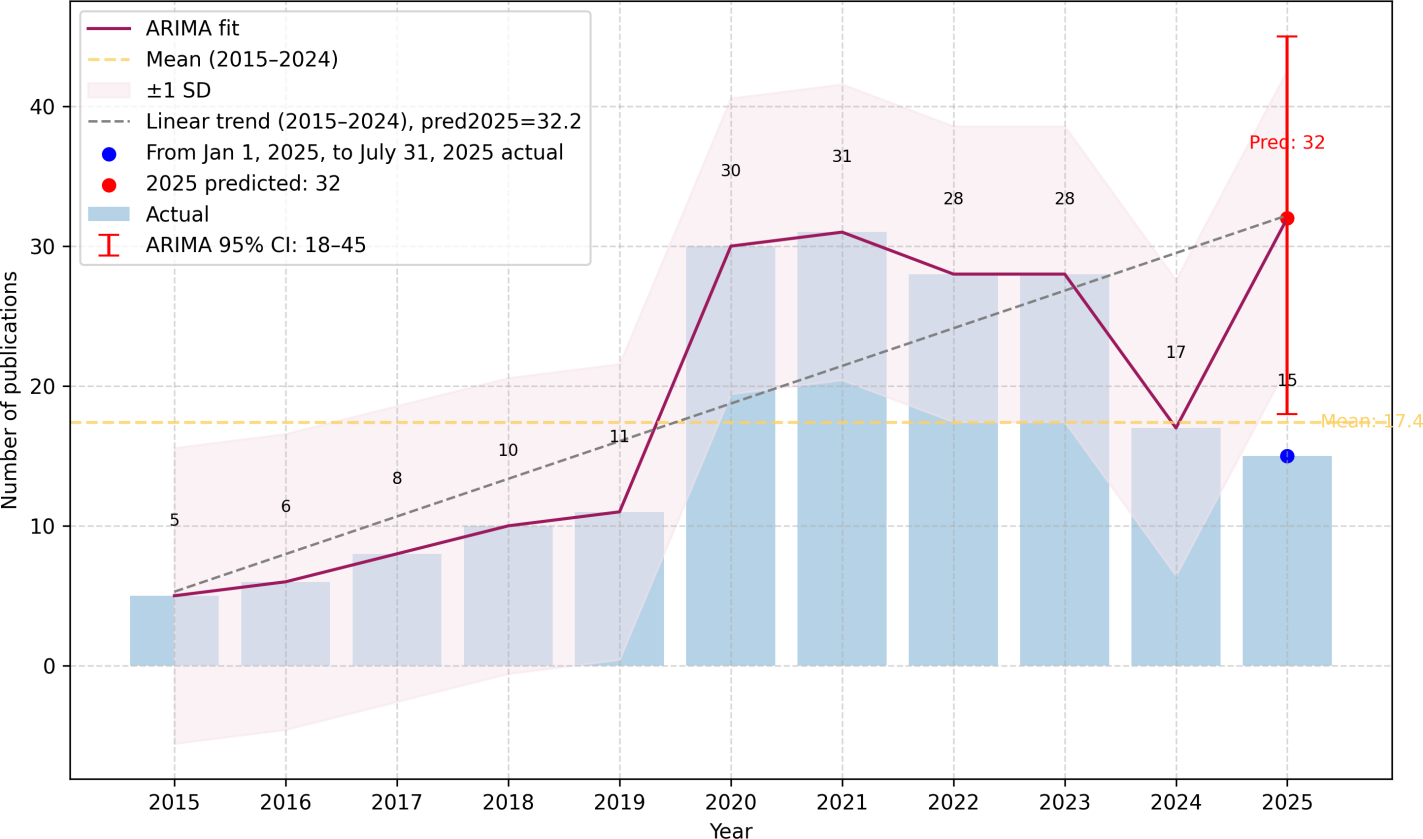
Annual publication trends and autoregressive integrated moving average (ARIMA)-based exploratory projection (January 1, 2015, to July 31, 2025, including the predicted number for 2025). In this study, each year is treated as a single observation, resulting in a short time series based on annual aggregation. The ARIMA model serves only as an exploratory projection, yielding a broad prediction interval. The predicted counts for 2025 should be interpreted with caution.

As shown in Figure 2 and Table S1 in Multimedia Appendix 6, annual publication trends in SMM exhibited a consistent upward trajectory over the past decade. Between 2015 and 2024, the annual number of PubMed-indexed articles averaged 17.4. By July 31, 2025, 15 publications had already been recorded. Using an ARIMA-based exploratory projection fitted to the annual series, we estimated approximately 32 publications for 2025, with a wide 95% prediction interval (18-45) reflecting uncertainty due to the short annual time series. Overall, these findings indicate sustained growth and underscore the growing scholarly interest in the field.

The distributions of countries, institutions, and journals are summarized in Table 1. The United States was the leading contributor (54/189, 28.6%), followed by China (27/189, 14.3%), Italy (11/189, 5.8%), and France (10/189, 5.3%). Among institutions, the University of Texas at Austin (5/189, 2.7%) and Kazan Federal University (3/189, 1.6%) appeared most frequently. The Journal of Medical Internet Research was the dominant journal (21/189, 11.1%), followed by the International Journal of Environmental Research and Public Health (7/189, 3.7%) and JMIR Public Health and Surveillance (7/189, 3.7%). Beyond these core contributors, a long tail of less-represented countries, institutions, and journals reflects the field’s broadening boundaries and thematic heterogeneity.

**Table 1.** Top 10 entities (countries, affiliations, and journals) by frequency in social media mining publications.

Variable	Value (N=189), n (%)
Country	
United States	54 (28.6)
China	27 (14.3)
Italy	11 (5.8)
France	10 (5.3)
United Kingdom	7 (3.7)
Canada	6 (3.2)
Korea	6 (3.2)
Republic of China (Taiwan)	5 (2.7)
Spain	5 (2.7)
Japan	4 (2.1)
Affiliation	
Center for Health Communication, The University of Texas at Austin, Austin, Texas, United States	5 (2.7)
Kazan Federal University, Kazan, Russian Federation	3 (1.6)
Center Régional de Pharmacovigilance, Hôpital Européen Georges-Pompidou, Paris, France	2 (1.1)
College of Nursing and Public Health, Adelphi University, Garden City, New York, United States	2 (1.1)
Department of Psychiatry, Singapore General Hospital, Singapore	2 (1.1)
Division of General Internal Medicine and Primary Care, Brigham and Women’s Hospital, Boston, Massachusetts, United States	2 (1.1)
Division of Rheumatology, Allergy and Immunology, Massachusetts General Hospital, Boston, Massachusetts, United States	2 (1.1)
Institute of Informatics and Telematics (IIT), National Research Council (CNR), Pisa, Italy	2 (1.1)
Kap Code, Paris, France	2 (1.1)
National Research University Higher School of Economics, Moscow, Russian Federation	2 (1.1)
Journal	
Journal of Medical Internet Research	21 (11.1)
International Journal of Environmental Research and Public Health	7 (3.7)
JMIR Public Health and Surveillance	7 (3.7)
Journal of Biomedical Informatics	7 (3.7)
Frontiers in Psychology	6 (3.2)
Journal of the American Medical Informatics Association: JAMIA	6 (3.2)
PeerJ Computer Science	6 (3.2)
Studies in Health Technology and Informatics	6 (3.2)
JMIR Infodemiology	4 (2.1)
Social Network Analysis and Mining	4 (2.1)

The author collaboration network in Figure S3 in Multimedia Appendix 1 displays a pattern in which a few highly connected researchers, such as Graciela Gonzalez-Hernandez, Davy Weissenbacher, and Abeed Sarker, occupy central hub positions, maintaining multiple cross-institutional collaborations. Their cooperative studies are summarized in Tables S2 and S3 in Multimedia Appendix 6, highlighting broader collaboration portfolios, notably those of Nathalie Texier and colleagues. Overall, the field is shaped by a small number of core investigators complemented by a long tail of occasional contributors. Importantly, this authorship structure is mirrored in citation patterns, which further reflect the influence and reach of these core groups.

Citation analyses highlighted both globally influential work and highly interconnected contributions within the field. As shown in Figure S4 in Multimedia Appendix 1, the ranking of publications by RCR identified a set of cornerstone articles, with articles by Ayyoubzadeh et al [38] on COVID-19 trend prediction and Nikfarjam et al [39] on adverse drug reactions consistently leading the field. Other high-RCR papers addressed vaccine hesitancy, mental health, and methodological advances, such as machine learning (ML) and natural language processing (NLP), reflecting both topical diversity and methodological innovation among the most impactful studies. Within the internal citation network (Figure S5 in Multimedia Appendix 1), the article by Nikfarjam et al [39] again occupied the central position with the most citations, followed by articles by Tapi Nzali et al [40] on breast cancer and Lazard et al [41] on public communication. Several COVID-19–related studies (eg, Li et al [42] and Zhang et al [43]) also ranked prominently, underscoring the pandemic’s role in driving recent scholarly influence.

The keyword statistics in Table 2 and Figure S6 in Multimedia Appendix 1 revealed both stable core terms and dynamically emerging concepts. As shown in Figure S6 in Multimedia Appendix 1, “social media,” “data mining,” and “natural language processing” consistently ranked among the most frequent keywords forming a foundation in SMM. In contrast, the occurrence of terms, such as “COVID-19,” “vaccine hesitancy,” and “mental health,” increased sharply in specific years, highlighting the responsiveness of the literature to external events and public health concerns. Certain topics (eg, “sentiment analysis”) extended beyond 2025, suggesting potential growth.

**Table 2.** Top 20 keywords in social media mining publications (2015‐2025).

Rank	Keyword	Overall frequency, n
1	Social media	84
2	Data mining	58
3	COVID-19	43
4	Natural language processing	36
5	Twitter	34
6	Sentiment analysis	25
7	Social media mining	24
8	Machine learning	22
9	Infodemiology	14
10	Topic modeling	10
11	Deep learning	9
12	Pandemics	9
13	Pharmacovigilance	9
14	Coronavirus	8
15	Mental health	7
16	Big data	6
17	Latent Dirichlet allocation	6
18	Public health	6
19	Reddit	6
20	Social networking	6

**Figure 3.**
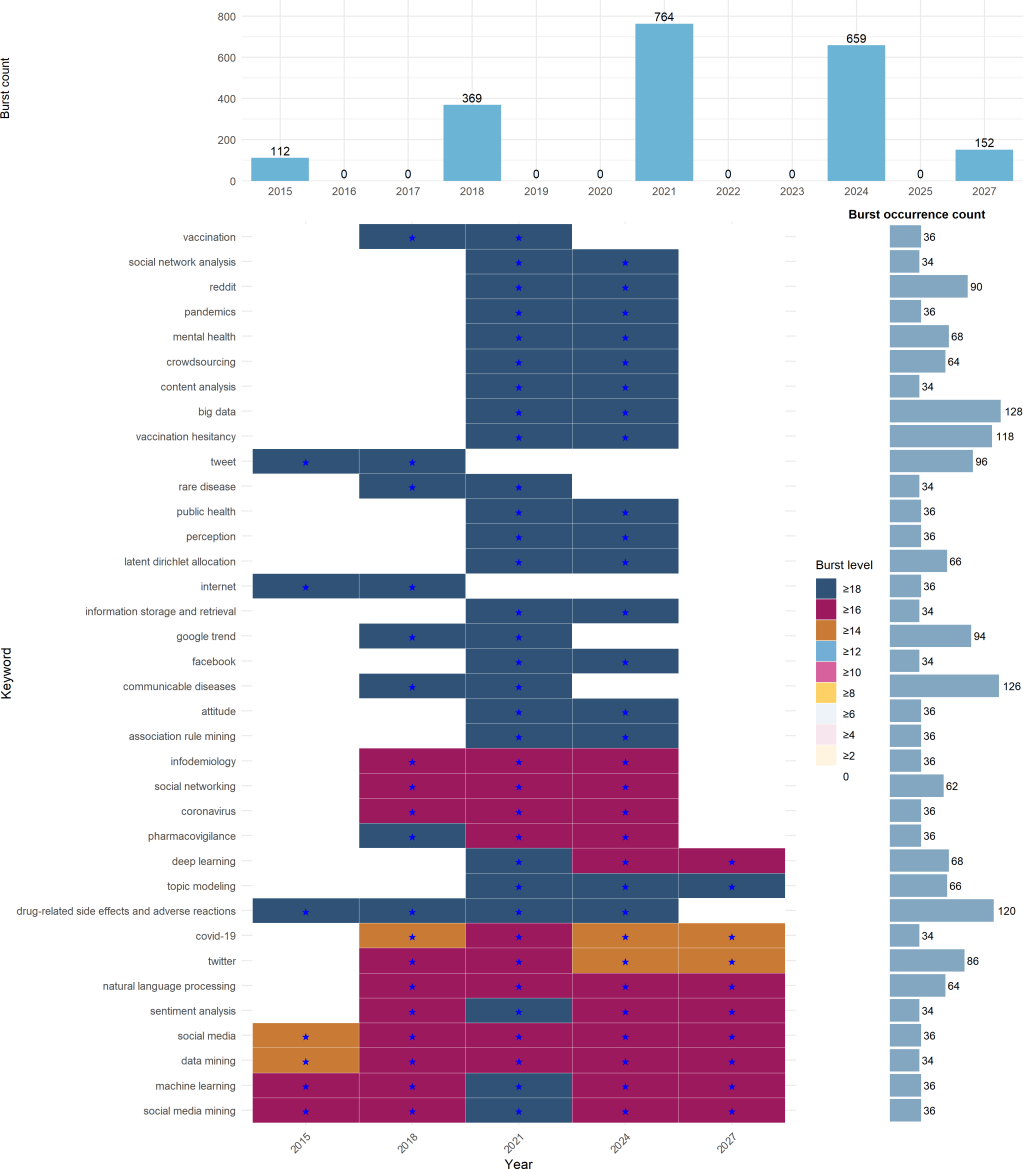
Heatmap of keyword burst events in PubMed literature. Each row represents a keyword identified from PubMed articles, and columns denote 2015 to 2025. The central heatmap displays burst periods detected by the Kleinberg burst detection algorithm under parameter configuration G0.5_W3_L1. Burst levels are shown in 10 discrete tiers with specific hex colors, where darker shades indicate stronger bursts (eg, level ≥18 in navy). Blue stars mark individual burst periods. The top bar plot shows the total number of keyword bursts per year, with counts labeled above each bar. The right bar plot indicates the total number of burst occurrences per keyword across all years. These bursts often correspond to emerging or intensifying topics in biomedical and social media research.

**Figure 4.**
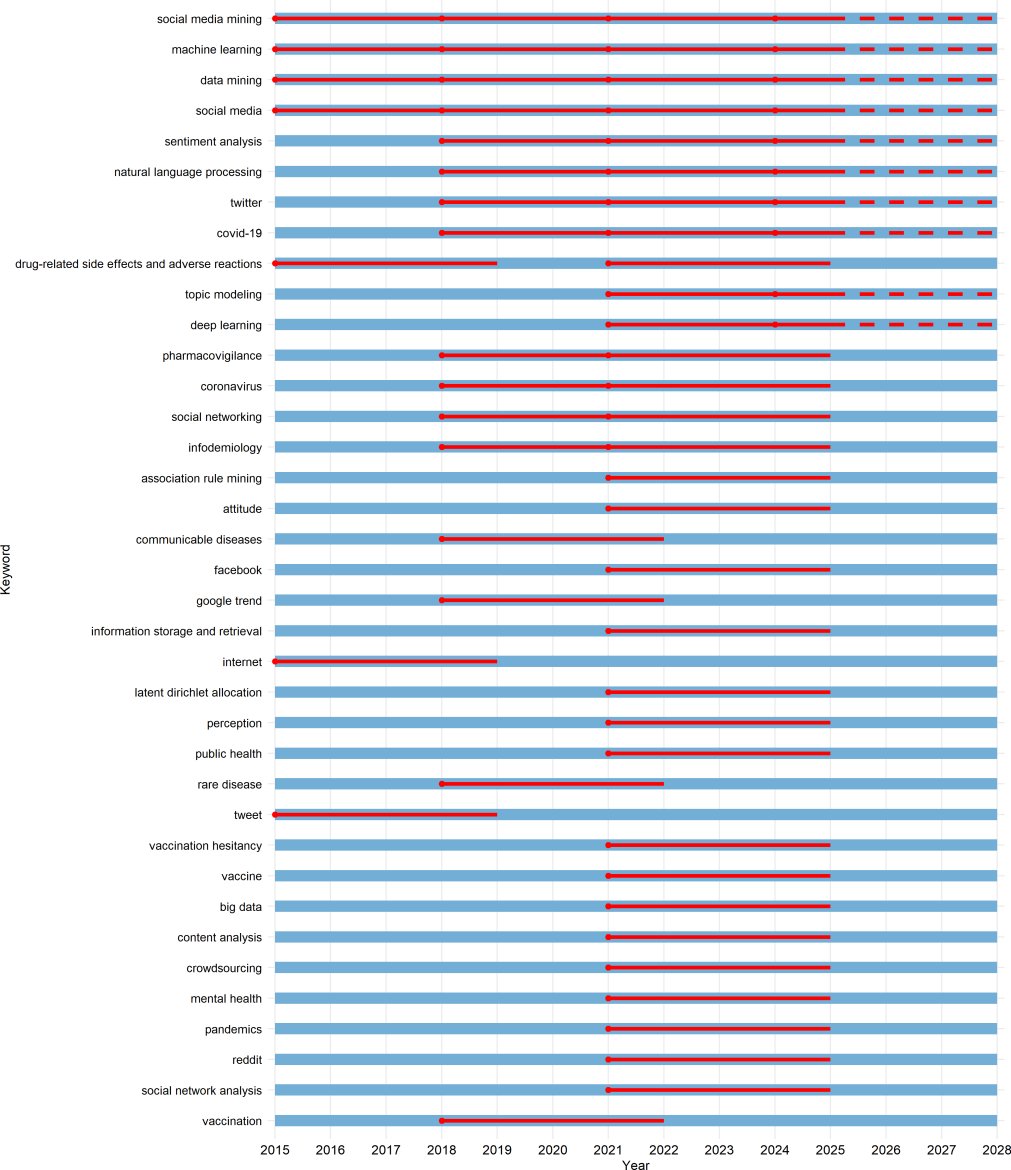
Burst timelines of keywords with burst levels ≥2 in social media mining from 2015 to 2027. The image shows the temporal burst patterns of 37 keywords identified as most significant in terms of burst strength. Each keyword is represented by a horizontal timeline from the start of 2015 to the end of 2027 (blue lines), with red solid lines indicating the duration of burst periods and red dots marking the onset of each burst. If a burst extends beyond the year 2025, the line becomes dashed to signify projected or ongoing trends. The visualization highlights the emergence and persistence of research interests within the field of digital health and social media analytics.

We applied the Kleinberg burst detection algorithm to capture topic dynamics. The resulting heatmap (Figure 3) and timeline (Figure 4) identified multiple high-intensity bursts, with peak activity observed in 2020‐2021. Burst events were not limited to pandemic-related terms, and method-focused keywords, such as “topic modeling” and “ML,” indicated that methodological innovation engages in dynamic interaction with real-world demands.

### Static Thematic Clustering, Strategic Positioning, and Intercluster Relationships

#### Overview

A total of 1157 author keywords were present in the included literature. Initially, low-frequency words with an occurrence frequency of ≤2 were removed, along with the 2 most frequently occurring words. This approach prevents noise from affecting the co-occurrence network and clustering results while also avoiding overdominating the network outcomes. Then, to prevent isolated or incidentally collinear edges from affecting structural similarity, a minimum co-occurrence threshold of 1 was set. Only keywords with edges present in both the structural and semantic spaces were retained. Finally, 78 high-quality keywords (Table 3) were included for subsequent analysis.

**Table 3.** Detailed metrics of keywords and clusters.

Cluster and keywords	Freq^a^, n	Overall degree	Network strength	Centroid distance^b^	Quadrant
Cluster 1^c^ (noise; n=19)					
Mental health	7	11	4.195	2.019	Core emerging hotspot (x≥0, y≥3.51, z≥0.5)
Big data	6	10	3.829	1.903	Potential breakthrough theme (x<0, y≥3.51, z≥0.5)
Public health	6	24	9.121	0.872	Core emerging hotspot (x≥0, y≥3.51, z≥0.5)
Depression	3	5	1.722	2.099	Peripheral mature theme (x≥0, y<3.51, z<0.5)
Google trend	3	7	2.460	1.330	Peripheral mature theme (x≥0, y<3.51, z<0.5)
Perception	3	15	5.166	1.918	Potential breakthrough theme (x<0, y≥3.51, z≥0.5)
Cyberincivility	2	3	1.705	1.758	Exploratory emerging theme (x<0, y<3.51, z≥0.5)
Digital health	2	3	1.199	1.822	Peripheral emerging theme (x≥0, y<3.51, z≥0.5)
Drug repositioning	2	6	0.983	2.100	Exploratory emerging theme (x<0, y<3.51, z≥0.5)
LSTM	2	7	2.198	0.980	Peripheral emerging theme (x≥0, y<3.51, z≥0.5)
Medium	2	1	0.492	0.989	Peripheral obsolete theme (x<0, y<3.51, z<0.5)
Neoplasms	2	5	1.979	1.688	Exploratory emerging theme (x<0, y<3.51, z≥0.5)
Nurse	2	6	2.240	1.669	Peripheral emerging theme (x≥0, y<3.51, z≥0.5)
Republic of Korea	2	3	0.944	1.542	Exploratory emerging theme (x<0, y<3.51, z≥0.5)
Social networking site	2	3	1.705	1.661	Exploratory emerging theme (x<0, y<3.51, z≥0.5)
Suicide	2	4	1.426	2.450	Peripheral emerging theme (x≥0, y<3.51, z≥0.5)
Thematic analysis	2	6	2.165	1.401	Exploratory emerging theme (x<0, y<3.51, z≥0.5)
Web scraping	2	3	0.757	1.685	Exploratory emerging theme (x<0, y<3.51, z≥0.5)
YouTube	2	3	1.127	1.599	Exploratory emerging theme (x<0, y<3.51, z≥0.5)
Cluster 2^d^ (n=15)					
Natural language processing	36	41	13.465	0.752	Core emerging hotspot (x≥0, y≥3.51, z≥0.5)
Social media mining	24	27	8.489	0.067	Core emerging hotspot (x≥0, y≥3.51, z≥0.5)
Machine learning	22	28	8.820	0.473	Core emerging hotspot (x≥0, y≥3.51, z≥0.5)
Pharmacovigilance	9	8	2.776	0.511	Peripheral mature theme (x≥0, y<3.51, z<0.5)
Information storage and retrieval	5	6	2.311	0.511	Exploratory emerging theme (x<0, y<3.51, z≥0.5)
Drug-related side effects and adverse reactions	4	6	1.841	0.404	Peripheral emerging theme (x≥0, y<3.51, z≥0.5)
Rare disease	3	4	1.080	0.452	Peripheral obsolete theme (x<0, y<3.51, z<0.5)
Computational social science	2	2	0.360	0.551	Exploratory emerging theme (x<0, y<3.51, z≥0.5)
Epidemiology	2	4	1.285	0.788	Peripheral obsolete theme (x<0, y<3.51, z<0.5)
Patient forum	2	5	1.318	0.469	Exploratory emerging theme (x<0, y<3.51, z≥0.5)
Patient-reported outcome measures	2	6	1.959	0.840	Exploratory emerging theme (x<0, y<3.51, z≥0.5)
Post-acute COVID-19 syndrome	2	5	1.755	0.925	Exploratory emerging theme (x<0, y<3.51, z≥0.5)
Psychological	2	3	0.786	1.153	Peripheral emerging theme (x≥0, y<3.51, z≥0.5)
Quality of life	2	1	0.181	0.507	Exploratory emerging theme (x<0, y<3.51, z≥0.5)
Symptom	2	5	2.282	0.939	Exploratory emerging theme (x<0, y<3.51, z≥0.5)
Cluster 3^e^ (n=8)					
Pandemics	9	23	8.704	0.567	Core emerging hotspot (x≥0, y≥3.51, z≥0.5)
Vaccination	6	18	7.074	0.347	Core emerging hotspot (x≥0, y≥3.51, z≥0.5)
Vaccination hesitancy	4	7	2.706	0.163	Peripheral emerging theme (x≥0, y<3.51, z≥0.5)
Attitude	3	14	5.578	0.340	Potential breakthrough theme (x<0, y≥3.51, z≥0.5)
SARS-CoV-2	3	13	5.132	0.150	Potential breakthrough theme (x<0, y≥3.51, z≥0.5)
Human	2	4	1.927	0.480	Exploratory emerging theme (x<0, y<3.51, z≥0.5)
Influenza	2	4	1.927	0.480	Exploratory emerging theme (x<0, y<3.51, z≥0.5)
Twitter mining	2	4	1.447	0.411	Exploratory emerging theme (x<0, y<3.51, z≥0.5)
Cluster 4^f^ (n=13)					
COVID-19	43	49	16.294	0.326	Core emerging hotspot (x≥0, y≥3.51, z≥0.5)
Twitter	34	37	13.168	0.829	Core emerging hotspot (x≥0, y≥3.51, z≥0.5)
Infodemiology	14	25	8.831	0.220	Core emerging hotspot (x≥0, y≥3.51, z≥0.5)
Topic modeling	10	18	6.685	0.678	Core emerging hotspot (x≥0, y≥3.51, z≥0.5)
Coronavirus	8	13	5.151	0.173	Core emerging hotspot (x≥0, y≥3.51, z≥0.5)
Tweet	6	15	5.508	0.603	Immature but declining theme (x<0, y≥3.51, z<0.5)
Public opinion	4	5	1.906	0.800	Exploratory emerging theme (x<0, y<3.51, z≥0.5)
Communicable diseases	3	4	1.704	0.291	Peripheral mature theme (x≥0, y<3.51, z<0.5)
Internet	3	5	2.005	0.673	Peripheral mature theme (x≥0, y<3.51, z<0.5)
Communication	2	7	2.648	0.245	Exploratory emerging theme (x<0, y<3.51, z≥0.5)
Electronic nicotine delivery systems	2	4	1.250	0.668	Peripheral obsolete theme (x<0, y<3.51, z<0.5)
Geolocation	2	6	1.965	0.770	Exploratory emerging theme (x<0, y<3.51, z≥0.5)
Neural network	2	6	2.124	0.273	Exploratory emerging theme (x<0, y<3.51, z≥0.5)
Cluster 5^g^ (n=10)					
Content analysis	5	28	10.239	0.354	Potential breakthrough theme (x<0, y≥3.51, z≥0.5)
Facebook	5	10	2.980	0.519	Exploratory emerging theme (x<0, y<3.51, z≥0.5)
Adolescent	3	9	3.117	0.618	Exploratory emerging theme (x<0, y<3.51, z≥0.5)
Association rule mining	3	7	2.490	0.518	Exploratory emerging theme (x<0, y<3.51, z≥0.5)
Health communication	2	3	1.296	0.175	Exploratory emerging theme (x<0, y<3.51, z≥0.5)
Health promotion	2	5	1.930	0.876	Exploratory emerging theme (x<0, y<3.51, z≥0.5)
Smoking	2	9	2.878	0.387	Exploratory emerging theme (x<0, y<3.51, z≥0.5)
Social media analysis	2	6	1.984	0.450	Exploratory emerging theme (x<0, y<3.51, z≥0.5)
Tobacco	2	8	2.620	0.498	Exploratory emerging theme (x<0, y<3.51, z≥0.5)
User engagement	2	3	1.255	0.311	Exploratory emerging theme (x<0, y<3.51, z≥0.5)
Cluster 6^h^ (n=13)					
Sentiment analysis	25	21	7.476	0.462	Core emerging hotspot (x≥0, y≥3.51, z≥0.5)
Deep learning	9	15	5.526	0.351	Potential breakthrough theme (x<0, y≥3.51, z≥0.5)
Latent Dirichlet allocation	6	19	7.123	0.805	Core emerging hotspot (x≥0, y≥3.51, z≥0.5)
Reddit	6	14	4.554	0.699	Potential breakthrough theme (x<0, y≥3.51, z≥0.5)
Social networking	6	9	3.350	0.627	Exploratory emerging theme (x<0, y<3.51, z≥0.5)
Social network analysis	5	3	1.074	0.382	Exploratory emerging theme (x<0, y<3.51, z≥0.5)
Vaccine	5	19	7.156	1.018	Potential breakthrough theme (x<0, y≥3.51, z≥0.5)
Crowdsourcing	4	9	2.570	0.603	Exploratory emerging theme (x<0, y<3.51, z≥0.5)
Network analysis	3	8	2.729	0.637	Exploratory emerging theme (x<0, y<3.51, z≥0.5)
HPV	2	4	1.603	0.453	Peripheral obsolete theme (x<0, y<3.51, z<0.5)
Recurrent neural networks	2	3	0.789	0.382	Exploratory emerging theme (x<0, y<3.51, z≥0.5)
Social media data	2	3	0.746	0.202	Exploratory emerging theme (x<0, y<3.51, z≥0.5)
Word embedding	2	2	0.671	0.639	Exploratory emerging theme (x<0, y<3.51, z≥0.5)

a Freq: frequency.

b Representative terms can be identified from centroid distance values, providing both structural and semantic insights into each cluster. A smaller centroid distance indicates that a keyword is closer to the cluster center and thus more representative of the cluster’s overall theme.

c Cluster 1: Candidate incubator pool of peripheral cross-cutting topics in health-related social media mining.

d Cluster 2: Computational methods in health informatics.

e Cluster 3: Public attitudes and sociopsychological determinants.

f Cluster 4: Infodemiology and the COVID-19 information ecosystem.

g Cluster 5: Health communication and public health engagement.

h Cluster 6: Social media analysis and network methods.

**Figure 5.**
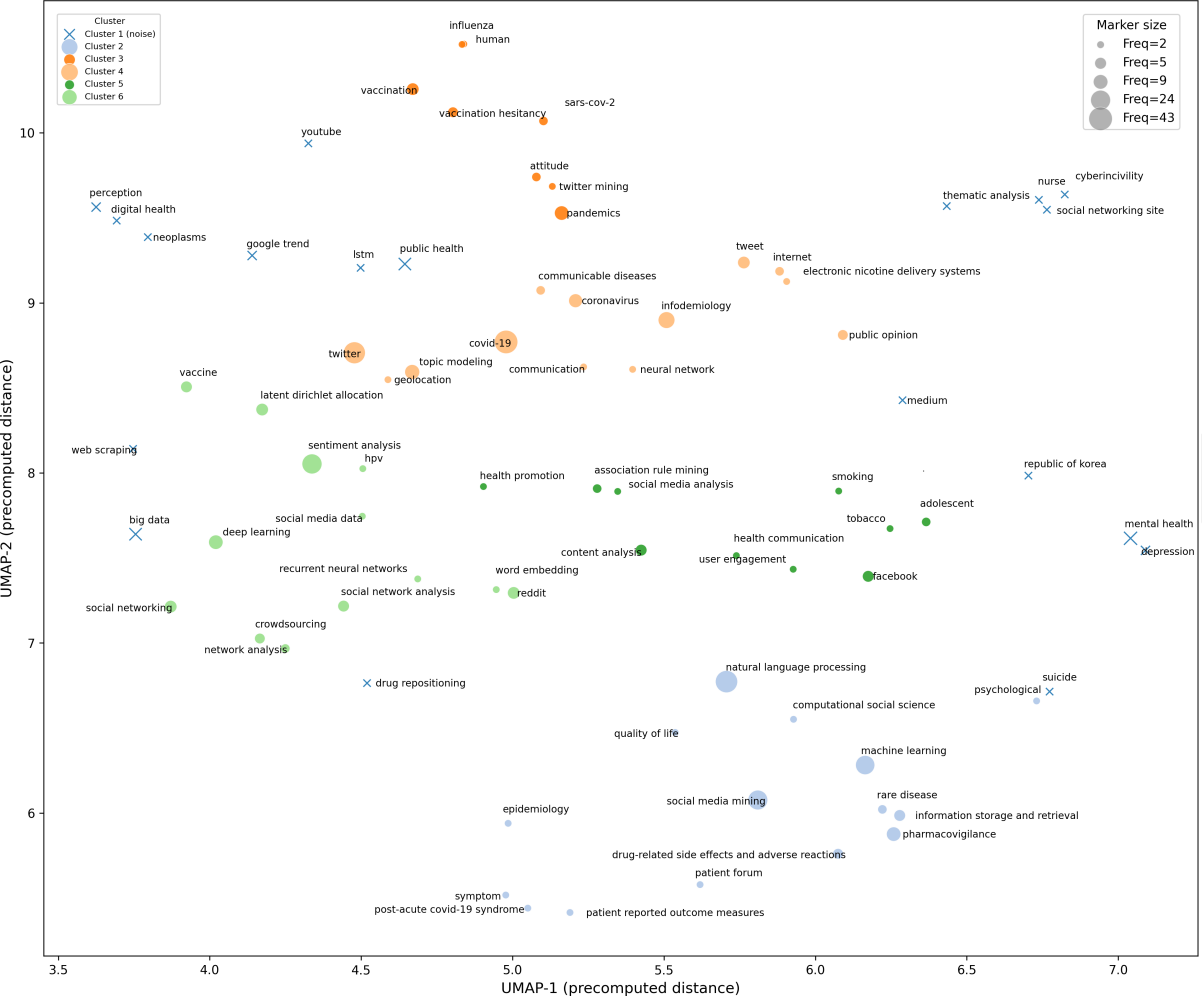
Keyword clusters identified by Uniform Manifold Approximation and Projection (UMAP)–Hierarchical Density-Based Spatial Clustering of Applications with Noise (HDBSCAN) with cluster-level metrics. This image illustrates the thematic structure obtained by performing semantic-structural integration of keywords, followed by UMAP to reduce high-dimensional similarity relationships in 2D space and HDBSCAN for density-based clustering. The horizontal and vertical axes represent the 2 UMAP embedding coordinates (UMAP-1 and UMAP-2), which are precomputed as 2D representations of distances. The relative positions of points on the plane indicate the comprehensive similarity between keywords (closer distances indicate greater semantic and co-occurrence similarity). Different colors denote distinct cluster labels (clusters 2‐6 represent assigned thematic clusters). The blue cross labeled cluster 1 (noise; HDBSCAN label=−1) indicates a set of keywords not stably assigned to any high-density cluster by HDBSCAN (unassigned/peripheral set), which does not equate to meaninglessness. Point size is proportional to keyword frequency (Freq) in the sample literature, reflecting each keyword’s relative importance and coverage within the dataset. Six labels were identified under this parameter configuration: 5 nonnoise thematic clusters and 1 unassigned set.

**Figure 6.**
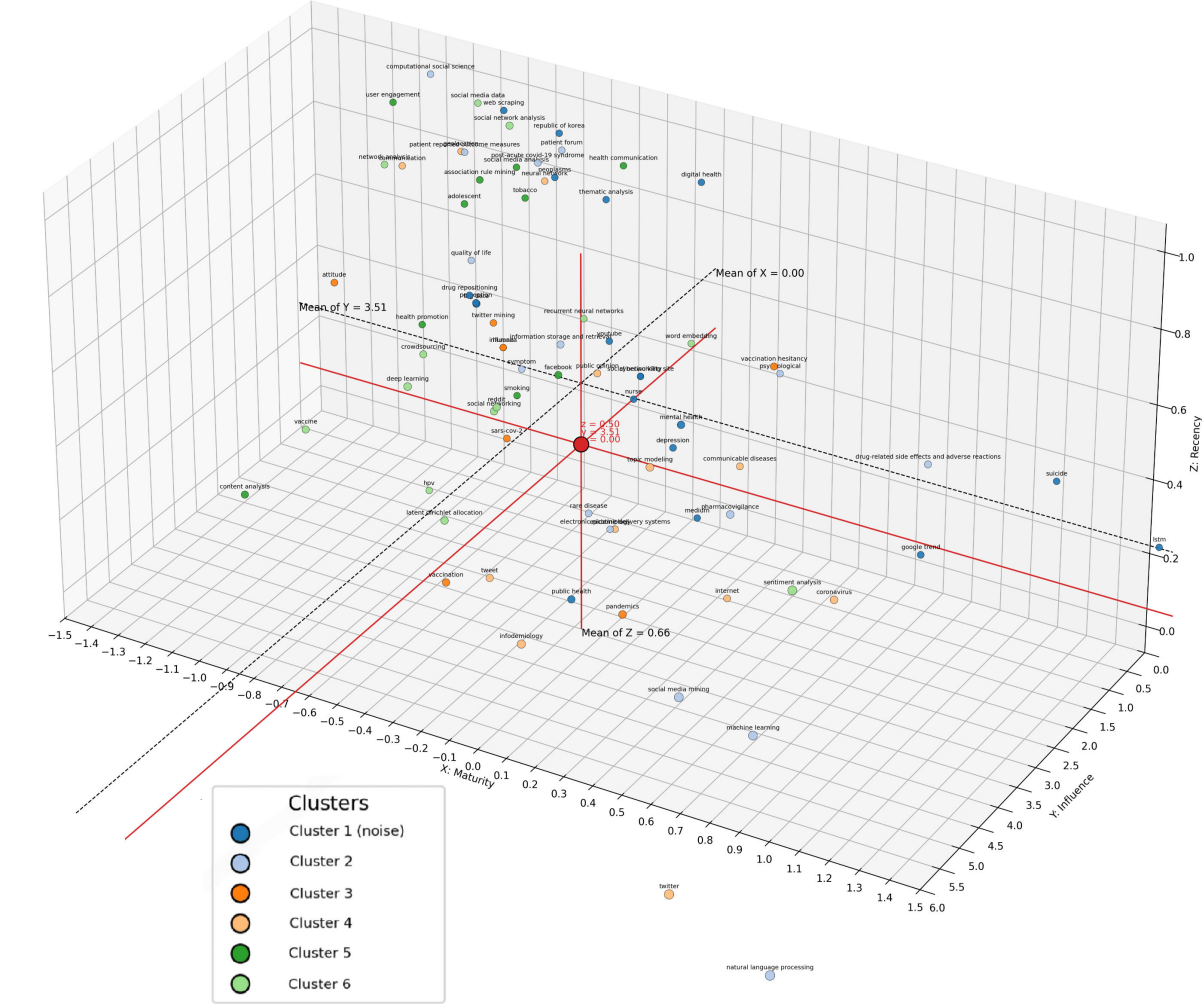
Three-dimensional thematic map of research on social media mining from January 1, 2015, to July 31, 2025. The image maps the keyword thematic structure identified by Uniform Manifold Approximation and Projection (UMAP)–Hierarchical Density-Based Spatial Clustering of Applications with Noise (HDBSCAN) onto a 3D strategic coordinate system to assess the historical accumulation, structural influence, and recent growth potential of different research directions. The x-axis (maturity) represents the degree of research accumulation associated with each keyword, and it is characterized by the number of papers associated with the keyword, namely its occurrence frequency, and is normalized. The y-axis (influence) represents the structural importance of the keywords within the co-occurrence networks, and it is represented by network connection strength, namely centrality metrics, and is normalized. The z-axis (recency) represents the recent activity level of the keywords, and it is represented by recency metrics, such as the proportion of publications in the last 5 years, and is normalized. Each point in the image represents a keyword, with color denoting its assigned cluster. Clusters 2‐6 represent assigned thematic clusters. Cluster 1 (noise) includes peripheral keywords that are not stably assigned, and it is used to present cross-thematic or sparse yet potentially valuable research signals. The red coordinate axis and central red dot denote the reference origin in 3D space. The black dashed planes mark the overall mean thresholds for the 3 axes (eg, mean of x=0.00, mean of y=3.51, and mean of z=0.66), which are used to divide the space into multiple strategic quadrants. This identifies distinct research directions such as core hotspots, potential breakthroughs, emerging frontiers, and declining themes.

The UMAP-HDBSCAN clustering of the author keywords yielded 6 thematic clusters (Figure 5 and Table 3) with detailed statistics (Table S4 in Multimedia Appendix 6). Their 3D strategic positioning has been summarized in Figure 6 (2D projections are shown in Figures S7-S9 in Multimedia Appendix 1) and Table 3.

#### Cluster 1 (Noise): Candidate Incubator Pool of Peripheral Cross-Cutting Topics in Health-Related SMM

The UMAP-HDBSCAN analysis identified 6 thematic clusters, with 1 cluster identified as an unassigned peripheral noise set (cluster 1). We retained this unassigned set as a cluster for structural interpretation, as it contained meaningful cross-thematic elements, keywords disrupted by major event windows, and early-stage keywords. Its retention helped prevent information loss and aided in the capture of potentially meaningful cross-cutting and early-stage signals that did not form stable dense regions in the embedding space. The supporting rationale is presented in Multimedia Appendix 7. This cluster is a collection of heterogeneous, peripheral, and thematically varied research that cannot be categorized into any other single specialized cluster. The themes in this group are highly fragmented, with representative keywords ranging from “public health,” “mental health,” and “perception” to specific technologies like “big data” and “LSTM.” However, this “noise” cluster is not without value. It represents the emerging and cross-disciplinary signals of the field, serving as an “incubator” for potential research directions and an important reference for defining the boundaries of current core research areas. The majority of keywords in this group fell within the *exploratory emerging theme* quadrant (x<0, y<3.51, z≥0.5), representing recently active, immature research topics such as “neoplasms,” “YouTube,” etc. Therefore, we treated cluster 1 as a candidate incubator pool of peripheral cross-cutting topics that may contain topics that could evolve into coherent thematic clusters as the field matures, and its detailed interpretation is provided in Multimedia Appendix 7.

#### Cluster 2: Computational Methods in Health Informatics

Cluster 2 represents the technical and methodological foundations of the entire research field. It focuses on the application of core computational technologies, particularly “SMM,” “NLP,” and “ML,” to diverse health scenarios. Its research involves the development and application of these technologies to address specific issues such as “drug-related side effects and adverse reactions,” “pharmacovigilance,” identification of “rare diseases,” and analysis of “patient-reported outcomes” and “quality of life.” It aims to efficiently extract valuable health insights from vast amounts of unstructured text data, serving as the technical foundation for all other applied clusters. Most topics in this cluster were located in the *exploratory emerging theme* quadrant (x<0, y<3.51, z≥0.5), particularly the keywords representing patient-reported outcomes and the topic about symptoms.

#### Cluster 3: Public Attitudes and Sociopsychological Determinants

Cluster 3 represents a mature field dedicated to analyzing public psychology and reactions during health crises, particularly “pandemics” and “vaccination hesitancy.” Using methods like “Twitter mining,” it delves into public “attitudes,” sentiments, and concerns. This cluster aims to uncover the deep-seated sociopsychological drivers behind public health attitudes, providing scientific evidence for developing targeted communication strategies and public policies.

#### Cluster 4: Infodemiology and the COVID-19 Information Ecosystem

Cluster 4 represents the most critical and dynamic research frontier, driven by the major global health crisis of “COVID-19.” This cluster focuses on “infodemiology,” examining how various types of information, including misinformation, spread, evolved, and influenced public opinion on social media platforms like Twitter during the pandemic. This is a quintessential crisis-driven research domain characterized by high timeliness and strategic importance. It aims to provide real-time monitoring tools and response strategies for addressing information challenges during public health emergencies.

#### Cluster 5: Health Communication and Public Health Engagement

Cluster 5 represents a highly application-oriented proactive intervention field, centered on designing and evaluating effective digital health communication campaigns. Using platforms like Facebook as its primary arena and focusing on specific issues, such as tobacco control, it uses “user engagement” as a key metric. Through methods like “content analysis,” it explores which information frameworks most effectively reach and influence target audiences (eg, adolescents). Its findings offer direct practical guidance for public health institutions to optimize routine online health promotion efforts. All the themes in this group represent highly promising research trends for the coming decades.

#### Cluster 6: Social Media Analysis and Network Methods

Cluster 6 represents a group of advanced analytical techniques, deepening and advancing the methodologies explored in cluster 2, which involves general computational methods. It incorporates established technologies like “sentiment analysis” and “topic modeling” while introducing more sophisticated approaches such as “deep learning” and “social network analysis.” Its core distinction lies in focusing not merely on the content of the text, but on revealing the mechanism of information dissemination. By analyzing user connections and the network structures of information flow, it uncovers the transmission dynamics of complex digital discourse.

#### Global Thematic Landscape and Cross-Cluster Bridging

Overall, the representative keywords in each quadrant clearly outline the stratified research landscape: flagship core themes, such as NLP, ML, and COVID-19, drive the development of the entire field; traditional pillars like content analysis and health communication maintain high influence but have waned in popularity; emerging potential topics, such as vaccination hesitancy and deep learning, are on the rise; and marginal incubation and dormant areas provide a window for observing future dynamic evolution.

To further explore the relationships between different thematic clusters, we measured the cross-cluster coupling strength between thematic clusters in the keyword co-occurrence network, defined as the sum of weights on cross-cluster edges connecting 2 distinct clusters, and identified bridging keywords contributing the most to coupling (Figure S10 in Multimedia Appendix 1 and Tables S5-S8 in Multimedia Appendix 6). The results indicated that cluster 4 occupied a central hub position in cross-cluster interactions, exhibiting strong coupling with multiple thematic clusters.

Among all cluster pairs, the highest coupling strength was observed between cluster 1 and cluster 4 (cross-cluster strength=10.649), followed by cluster 4 and cluster 6 (10.404), cluster 3 and cluster 4 (7.989), cluster 2 and cluster 4 (7.216), and cluster 2 and cluster 6 (6.967; Table S6 in Multimedia Appendix 6). Overall, these highly coupled clusters primarily revolve around cross-cluster co-occurrence links between cluster 4 and methodological clusters (cluster 2/6). It should be noted that cluster 1 consists of a heterogeneous set of peripheral keywords annotated by HDBSCAN. Therefore, its high coupling with core clusters more likely reflects conceptual convergence and enhanced cross-domain connections within specific event windows, such as the peak of pandemic-related research, rather than a single cohesive theme.

Furthermore, we analyzed the top 5 cross-cluster strength pairs by identifying their pair-specific bridging keywords (Table 4 and Table S7 in Multimedia Appendix 6). For the cluster 1-cluster 4 pair, the primary bridging keywords were COVID-19 (contribution=3.052), public health (2.104), Twitter (2.018), topic modeling (1.549), and coronavirus (1.429). For cluster 4‐cluster 6, the bridging keywords were Twitter (2.724), COVID-19 (2.540), deep learning (2.271), sentiment analysis (1.650), and topic modeling (1.646). For cluster 3-cluster 4, the bridging keywords were COVID-19 (3.013), pandemics (2.182), Twitter (1.973), attitude (1.532), and tweet (1.440). For cluster 2-cluster 4, the bridging keywords primarily included NLP (3.031), COVID-19 (2.081), information epidemiology (1.541), Twitter (1.208), and ML (1.135). For cluster 2-cluster 6, the bridging keywords included NLP (2.476), SMM (2.436), deep learning (1.360), sentiment analysis (1.359), and LDA (1.288). These findings reflect significant tool and algorithm sharing across methodological clusters.

**Table 4.** Top 5 intercluster pairs (ranked by cross-cluster strength) with their top 5 pair-specific bridging keywords.

Cluster pair and keyword	Source cluster/keyword cluster	Keyword pair contribution^a^	Cross-cluster strength^b^
Cluster 1^c^ (noise)-cluster 4^d^			10.649
COVID-19	Cluster 4	3.052	
Public health	Cluster 1 (noise)	2.104	
Twitter	Cluster 4	2.018	
Topic modeling	Cluster 4	1.549	
Coronavirus	Cluster 4	1.429	
Cluster 4-cluster 6^e^			10.404
Twitter	Cluster 4	2.724	
COVID-19	Cluster 4	2.540	
Deep learning	Cluster 6	2.271	
Sentiment analysis	Cluster 6	1.650	
Topic modeling	Cluster 4	1.646	
Cluster 3^f^-cluster 4			7.989
COVID-19	Cluster 4	3.013	
Pandemics	Cluster 3	2.182	
Twitter	Cluster 4	1.973	
Attitude	Cluster 3	1.532	
Tweet	Cluster 4	1.440	
Cluster 2^g^-cluster 6			7.216
Natural language processing	Cluster 2	2.476	
Social media mining	Cluster 2	2.436	
Deep learning	Cluster 6	1.360	
Sentiment analysis	Cluster 6	1.359	
Latent Dirichlet allocation	Cluster 6	1.288	
Cluster 2-cluster 4			6.967
Natural language processing	Cluster 2	3.031	
COVID-19	Cluster 4	2.081	
Infodemiology	Cluster 4	1.541	
Twitter	Cluster 4	1.208	
Machine learning	Cluster 2	1.135	

a Keyword pair contribution represents the contribution of the keyword to the cross-cluster coupling strength of the cluster, which is part of the cumulative contribution to cross-cluster edge weights.

b Cross-cluster strength is the sum of weights of cross-cluster edges connecting keywords from 2 distinct thematic clusters, which is used to quantify the degree of overlap between thematic clusters.

c Cluster 1: Candidate incubator pool of peripheral cross-cutting topics in health-related social media mining.

d Cluster 4: Infodemiology and the COVID-19 information ecosystem.

e Cluster 6: Social media analysis and network methods.

f Cluster 3: Public attitudes and sociopsychological determinants.

g Cluster 2: Computational methods in health informatics.

At the global level, keywords with the highest bridging strength clustered around event-driven themes and generic methodological/platform elements (Table S8 in Multimedia Appendix 6): COVID-19 (cross-cluster strength=12.520), NLP (10.642), Twitter (9.408), public health (7.669), content analysis (7.472), and pandemics (7.035). These bridging keywords jointly constructed a cross-cluster connection framework centered on event topics, platform data, and computational methods.

### Dynamic Temporal Analysis

We divided the study period into 3 slices using the explosive growth time stamp as the temporal boundary: 2015‐2019, 2020‐2023, and 2024-2025 (July 31, 2025). For each time slice, we scanned K∈[2.20]. The Silhouette and Calinski-Harabasz (CH) indices were noninformative in the spectral space (yielding values near zero or remaining constant), and thus, we prioritized modularity and used the Davies-Bouldin Index elbow as a safeguard against overfragmentation. The decision to fix the number of spectral clusters at K=3 (2015‐2019), K=8 (2020‐2023), and K=5 (2024‐2025) was guided by systematic evaluations across multiple criteria. While the Silhouette and CH indices were largely uninformative in the spectral embedding space, modularity consistently exhibited clear local maxima at these values, and Davies-Bouldin curves indicated elbows in the same vicinity. Selecting K at these points avoided both undersegmentation and overfragmentation while maximizing structural separation and interpretability. This choice ensured that each slice captured the dominant thematic structure of the period—technology-driven methodological consolidation before 2020, problem-driven thematic diversification from 2020 to 2023, and thematic convergence and strategic priorities after 2023. Figures S11-S14 in Multimedia Appendix 1 reveal the thematic changes across the 3 time slices.

#### Technology-Driven Methodological Consolidation (2015-2019)

During this phase, research themes emerged from the convergence of ML and NLP technological advancements with specific needs in pharmacovigilance and rare diseases. Though studies were relatively fragmented, they exhibited strong purposefulness, with technology driving applications. While this marked only the foundational stage of methodology and emerging applications, core methodological frameworks began to come into focus.

#### Problem-Driven Thematic Diversification (2020-2023)

The COVID-19 pandemic, as a black swan event, provided unprecedented data, attention, and application scenarios for the field. Consequently, COVID-19 became the absolute domain focus, leading to explosive growth in research scope and rapid diversification of research themes. This phase was problem-driven—specifically pandemic-driven—mobilizing all methodological approaches (ML, NLP, and sentiment analysis) and application directions (vaccines, information monitoring, and public attitudes) to meet its requirements. This process fostered a mature, complex, and highly differentiated research ecosystem.

#### Thematic Convergence and Strategic Priorities (2024-2025)

As the pandemic’s impact began to recede, research themes started to reconsolidate. Methods like ML, NLP, and content analysis remained central. This indicates that technologies validated and refined during the pandemic, such as sentiment analysis and deep learning, are maturing and being systematically applied to health research. Furthermore, the latest research no longer pursues isolated hot topics, and instead, it applies validated methodologies to prior, long-term public health challenges like “vaccines” and “mental health.”

#### Temporal Dynamics and Their Alignment With the Global Structure

Temporal spectral clusters aligned strongly with the global UMAP-HDBSCAN structure, with most spectral clusters mapping to a single global cluster at Jaccard scores above 0.3. The methodological cluster consistently matched the global “ML/NLP-driven social media analysis” domain. On the other hand, COVID-19 and mental health clusters overlapped with the global “Public health and pandemic response” and “Psychosocial health” domains, respectively.

Compared with the 3D strategic coordinate map, temporal slices provided a dynamic counterpart. The 2015‐2019 clusters coincided with the map’s high-maturity but low-recency quadrant, representing methodological foundations. The 2020‐2023 diversification overlapped with clusters in the high-influence and high-recency space, reflecting crisis-driven impact. The 2024‐2025 convergence mapped onto the high-maturity and moderate-recency zone, indicating thematic stabilization.

### Validation

We conducted sensitivity analyses on burst detection and clustering hyperparameters to validate the methods and findings at the macro-level.

In the parameter sensitivity analysis for burst detection, we selected the configuration with *γ*=0.5, a slice width of 3 years, and a minimum burst length of 1, which demonstrated strong overall performance. This setting achieved optimal values in coverage (1.0) and realism (1.8), ensuring that detected bursts were both comprehensive and plausible. Although its standalone stability scores (Jaccard=0.108; Spearman=0.259) appeared modest, cross-parameter comparisons (Table S9 in Multimedia Appendix 6) revealed that alternative configurations yielded Jaccard similarities ≥0.97 and Spearman correlations of approximately 0.70 with the baseline, confirming that the results are consistently reproducible across the parameter space. Thus, G0.5_W3_L1 was chosen as the primary setting.

The internal metrics for clustering evaluation are presented in Table S10 in Multimedia Appendix 6. As can be seen from the table, the balanced version with K=6 showed slightly better internal separation perception. In contrast, the fine version maintained “good” performance across major metrics while improving interpretability by an order of magnitude. Additionally, this study conducted 3 independent runs of fine_K6, all yielding nearly identical scores and key metrics. This indicates that the performance of fine_K6 is robust and insensitive to minor feature enhancements.

To assess the external validity of our clustering, we examined the relationship involving keyword network centrality and compared RCR distributions across clusters. As shown in Figure S15 in Multimedia Appendix 1, keyword strength was significantly positively correlated with mean RCR (Spearman ρ=0.49; *P*<.05), indicating that keywords occupying more central positions in the co-occurrence network tended to be associated with a higher citation impact (Figure S15 in Multimedia Appendix 1). Moreover, RCR distributions varied substantially across clusters (Figure S15 in Multimedia Appendix 1). Cluster 2 exhibited the highest citation impact (median RCR of approximately 1.5, with maximum values approaching 3), clearly exceeding other clusters. In contrast, clusters 1 and 4 showed lower mean RCR values (approximately 0.5‐0.7), while noise (cluster 1) had the lowest citation impact (mean RCR of approximately 0.45). Together, these findings demonstrate that the identified clusters not only reflect structural differences within the keyword network but also align with external citation impact, thereby validating the robustness and scientific relevance of our approach.

To enhance the interpretability of the clusters beyond quantitative indicators, we performed micro-level interpretive triangulation by systematically analyzing the introduction, contribution, limitations, and future directions reported in 28 representative studies (Multimedia Appendix 4). The content analysis suggested that the thematic boundaries identified by clustering correspond closely to coherent conceptual domains. First, studies assigned to cluster 2 (NLP/ML-driven social media analysis) consistently emphasized methodological innovation. This validates the cluster’s positioning as a methodological core in the strategic coordinate map where ML and NLP occupy high-influence and high-novelty regions [39,44]. Second, studies linked to clusters 1 and 2 highlighted empirical applications such as psychological health and suicide-related discussions [9,45]. Their reported contributions emphasized the utility of social media for monitoring population health behaviors, aligning with our dynamic slicing results where such content clusters emerged strongly during the COVID-19 period (2020‐2023) and then diversified into related subthemes. Third, the limitations articulated in these studies mirror the structural signals captured by clustering. Frequent reliance on Twitter and US-based data explains why Twitter appeared as a central hub in cluster 2 [7,46], while non-Twitter platforms (eg, Weibo, Facebook, and Google Maps reviews) were less frequently studied, corresponding to their marginal or emerging placement in the coordinate map. Similarly, methodological constraints in sentiment analysis, cross-lingual generalizability, and demographic representativeness corroborated the lower maturity scores of several peripheral clusters. Finally, the future research directions proposed by the authors include cross-platform data integration [7,46]. Deep learning methods, population stratification, and longitudinal prediction directly overlap with the potential breakthroughs located in the high-influence but low-maturity quadrants of our strategic map (eg, vaccine attitudes, digital health, and Weibo-based research). This convergence suggests that our framework not only captures the current structure of the field but also anticipates trajectories already envisioned by domain experts.

Collectively, this micro-level evidence mapping supports the interpretability of the thematic clusters and provides qualitative context that complements the quantitative indicators and external validation results, without being used as confirmatory evidence of model correctness. It also demonstrates that the clusters are semantically meaningful and consistent with the intellectual agenda of the field, thereby strengthening confidence in both the static and dynamic analyses.

## Discussion

### Principal Findings

This study used a hybrid methodology that combined ML-based clustering with qualitative evidence mapping to systematically draw a strategic research landscape of SMM. The findings clearly demonstrated that this field has evolved into a complex and coherent ecosystem. This ecosystem revolves around “infodemiology” as its absolute core, powered by a dual engine of “computational methods” and “analytical techniques,” extending its influence toward multiple critical application frontiers, including pharmacovigilance, public listening, and health management. Our findings not only depict the current state of the field but also reveal its underlying driving mechanisms and future developmental trajectory.

This study systematically analyzed health-related SMM literature collected in PubMed from 2015 to mid-2025, revealing significant evolutionary trends over the past decade. Overall, publication output showed steady growth, with explosive increases from 2019 to 2020, indicating SMM’s rising strategic importance as a tool for digital health and public health research. By integrating natural language model embeddings with network algorithm clustering, we identified 6 relatively independent yet interconnected thematic clusters. These spanned methodological anchors (NLP, ML, and social network analysis), socially driven topics (vaccine hesitancy and public listening), and priority themes reconverging in the postexplosive growth phase (health communication, mental health, and oncology). The distribution of these themes across a strategic 3D coordinate system (maturity, influence, and recency) further revealed the historical accumulation, current impact, and future potential of different research directions.

Time-slice analysis indicated that the knowledge structure of this field underwent three phased transitions: (1) the methodological consolidation from 2015 to 2019 laid the technical foundation; (2) the pandemic-driven surge from 2020 to 2023 accelerated thematic diversification, concentrating on various public health issues; and (3) by 2024‐2025, research refocused and converged on long-term strategic issues like cancer care and mental health. Through multiple validations, this study not only confirmed the robustness of clustering and strategic positioning but also demonstrated that these patterns align closely with the field’s actual development trajectory. Collectively, the study’s key findings provide a dynamic knowledge map for the digital health and health management field, spanning macro trends to micro evidence and offering a robust strategic reference for academic research, policy formulation, and practical applications.

### Comparison With Prior Work

Our finding that NLP and ML constitute the backbone of SMM aligns with recent reviews cataloging the rapid growth of computational methods in digital health [47-49]. However, some overviews have framed NLP as experimental in health care [50,51]. Our findings empirically demonstrate that, from a structural perspective, these technological pipelines have become firmly established as core architectures for social media–based health analytics (Table 3 and Figure 6). Additional empirical studies reinforce this interpretation. For instance, Ren et al [52] demonstrated the effectiveness of NLP and emotion-based deep learning (ML) for depression detection on Reddit, while Low et al [53] used NLP to identify vulnerable mental health support groups in online communities. Based on the above examples, these applications illustrate that methods are not only conceptually important but also practically operationalized in diverse health contexts. However, such research is often constrained by single-platform dependency and insufficient sample representativeness [52,54,55]. This is precisely why this strategic map positions this cluster as mature but still requiring methodological improvement and extended applications. Its strategic implications, from a sociological perspective, suggest that the dominance of a single-language Twitter, Facebook, Reddit, or Weibo corpus may structurally exclude speakers of other languages and marginalized voices, thereby exacerbating health inequalities [56-58]. From a health management perspective, mature NLP and ML pipelines are now deployable for real-time pharmacovigilance and attitude monitoring (eg, vaccine hesitancy dashboards), shifting management challenges from feasibility to governance [59-62]. Public health practice can benefit from earlier detection of emerging risks, enabling targeted, cost-effective preventive measures that reduce systemic burdens like disease incidence costs [63,64]. Collectively, the fundamental methods serve as strategic infrastructure in SMM.

Moreover, our analysis captured the explosion of crisis-driven research themes since 2020. Research during this period exhibited pronounced diversification and fragmentation, characterized by the rapid emergence of numerous exploratory subfields. Previous research accurately commented on this explosive growth but fragmented landscape in SMM [65]. However, to our knowledge, we provide data-driven evidence of an evolutionary trend in this field, shifting from fragmentation toward a focus on solving priority problems, based on our burst detection and time-slice analysis. Starting in 2024, as shown in our time-slice analysis, SMM research entered an era of integration characterized by a focus on addressing critical health issues. This signifies the field’s transition from an exploratory phase of data-driven research to a mission-driven phase oriented toward identifying problems to solve. While acknowledging the fragmented explosion as a necessary phase, we critically highlight the ongoing structural shift toward a more mature research paradigm.

### Methodological Highlights

Another major contribution of this study lies in methodological innovation. Unlike previous bibliometric research that relied primarily on co-occurrence–based keyword analysis, we proposed a fully automated, reproducible multilevel hybrid bibliometric methodology [17,18,24,29]. This approach of embedding-based mapping significantly enhances the interpretability and strategic value of the results while ensuring transparency and robustness [66].

First, at the semantic level, we used 2 natural language models—SPECTER2 and PubMedBERT—to vectorize titles and abstracts, aggregating them at the keyword level [66-68]. This approach overcomes the limitations of traditional methods that rely solely on manual synonym merging, enabling topic identification to capture latent semantic patterns and contextual associations [30]. In addition, at the structural level, we combined keyword co-occurrence networks with embedded similarity. Building upon the foundation, we constructed a hybrid similarity matrix that better aligns with actual disciplinary structures, achieving dual integration of semantics and structure.

Second, we used the nonparametric UMAP-HDBSCAN method to classify research themes. Systematic parameter grid searches and multiple random seed runs ensured the stability of clustering outcomes [69,70]. Unlike most studies relying solely on single clustering outputs, we further conducted internal (Jaccard, Silhouette, and Adjusted Rand Index) and external (RCR) validation. This was supplemented by micro-level evidence mapping and content analysis of representative papers. Such double validation could enhance clustering reliability.

Finally, this study proposes a 3D strategic coordinate map. By mapping clustering results onto the axes of maturity, influence, and recency, this framework transforms clustering outcomes into a knowledge map with strategic implications. This approach provides an intuitive tool for identifying hotspots, emerging frontiers, and declining fields.

### Future Research Trends

Based on the above analysis, we identified several key research gaps and propose the following future research directions:

- Research direction 1: While some studies have explored the use of SMM to understand cancer patients’ use of and perceptions toward traditional, complementary, and integrative medicine through micro-level content analysis, systematic evaluations of SMM research in chronic disease fields—represented by cancer—remain lacking [20,71,72]. The presence of “neoplasms” in cluster 1 of the static cluster analysis further corroborates this conclusion.
- Research direction 2: Most studies focus on correlational descriptions, with some literature exploring responses to public health initiatives among different populations. However, causal inference remains lacking, leaving unanswered questions such as “Can social media content truly demonstrate the impact of health promotion initiatives on changing health behaviors?” and “How do these behaviors change?” [73]. Future research could use mixed methods, integrating qualitative approaches to explore the underlying logic of health behavior change [61].
- Research direction 3: Future potential hotspots lie within cluster 1, such as SMM research on mental health and psychological disorders. This has been confirmed by the 3D coordinate map. Proactively leveraging new technologies like large language models to explore this domain using social media data will drive a shift from retrospective summarization to prospective prevention [74,75].
- Research direction 4: The 3D strategic coordinate map showed topics like adolescent and tobacco positioned in niche yet high-potential zones. This indicates that targeted health communication and health behavior–related issues, particularly those addressing specific populations, may evolve into core hotspots in future research [76].
- Research direction 5: Keywords like vaccination and attitude occupied either the high-impact or high-novelty quadrant in the 3D strategic coordinate map, marking a strategic frontier hotspot. Topics, such as vaccination hesitancy and public listening, experienced short-term bursts during the pandemic but landed closer to the peripheral emerging zone, indicating limited long-term stability [60,77]. Nevertheless, vaccine-related issues and public listening remain core academic focuses and will continue to evolve.

The outlined future directions can be consolidated into a transformable research framework. At the data level, comparative analyses involving cross-databases, multilingual datasets, and cross-platform sources should be encouraged to mitigate representational biases arising from single-platform or single-language sources while testing the transferability of thematic structures [78]. At the methodological level, semantic-structural integration can expand from static co-occurrence networks to dynamic networks to better capture thematic evolution and cross-cluster migration [79]. At the validation level, time slices and burst detection results can be compared against external reference data, such as policy timelines and significant events, while exploring event study methods to enhance interpretability and real-world applicability [73,75,80].

### Limitations

This study has several limitations. First, our analysis examined only English literature from PubMed, excluding other databases and other languages, which may not fully capture SMM applications across the entire field. In particular, this study relied on PubMed as its sole primary data source, resulting in a bias toward biomedical and clinical research. Consequently, representation from other fields was insufficient, potentially underestimating the scale of methodological innovations or interdisciplinary research. Second, due to the time lag in literature collection, recency may be underestimated. Third, the clustering analysis itself relied on keywords. While it considered the meaning of keywords within abstracts, it could not capture all the fine distinctions across studies. Moreover, we used keywords as analytical units in this study. Keywords themselves exhibit variations in naming and granularity, and have nonuniform usage. Hence, synonymous expressions may not be fully normalized. Although this study used PubMedBERT and SPECTER2 to learn structural and semantic similarity from title and abstract contexts, this approach only partially mitigates the fragmentation caused by relying on string matching. It cannot guarantee complete unification of all synonyms and polysemous terms, particularly when interdisciplinary terminology and task definitions are inconsistent. Beyond this, we did not conduct stratified external validation for each of the 3 time periods, such as comparing RCRs stage by stage. Such analyses may be statistically unstable with smaller samples and when reference metrics require time accumulation, potentially introducing systematic bias for the most recent stage, particularly the short and incomplete window of 2024‐2025 (July 2025). Future studies may explore stage-by-stage external validation with longer follow-up intervals. Additionally, SMM research can be influenced by structural biases associated with social media platforms and their user demographics [81]. For example, different platforms have different user demographics, and regional disparities and the digital divide may also affect the visibility of research topics. Our included SMM literature often relied on data from platforms like Twitter, Reddit, and Weibo, which differ in user profiles, languages, and geographic distributions. Consequently, which topics receive more research attention largely depends on the ease of data accessibility and user activity on these platforms. Therefore, the research findings presented here should primarily be understood as reflecting the knowledge structure at the level of academic publications, rather than being directly representative of the complete picture in the real world. Finally, as a bibliometric analysis, this study aimed to reveal correlations and trends rather than validate strict causal relationships.

### Conclusion

This study systematically revealed the knowledge structure, strategic positioning, and evolutionary trajectory of health-related SMM from 2015 to 2025 by developing a fully automated, reproducible hybrid bibliometric methodology. The innovation of this study lies in constructing a semantic-structural hybrid similarity matrix while enhancing method robustness through dual-level validation to reduce synonym fragmentation and parameter sensitivity. Unlike traditional bibliometric reviews that primarily rely on co-occurrence statistics or single-pass clustering, this study offers a decision-oriented, comprehensive analysis integrating interpretable thematic clustering, 3D strategic positioning, intercluster relationships, and time-slice analysis. Substantively, we found that the research evolved through 3 distinct periods: an initial phase of methodological consolidation, a middle phase where themes were rapidly diversified and fragmented, and a later phase of long-term priority problem-solving, such as mental health and cancer care. The 3D strategic coordinate map further indicates that differences in maturity, influence, and recency among various themes determine their potential and challenges for future development.

Our findings hold practical implications for public health, health research, and health care. For public health, the findings confirm the necessity of infodemiology and provide methodological support for developing precise, audience-segmented public health communication strategies. For health research, the knowledge map offered by this study can be used to strategically set research priorities and optimize the allocation of research resources. For health care, technologies in this field can be applied to support real-world health surveillance systems and data-driven decision-making. In particular, mature analytic pipelines in SMM can support not only the development but also the validation of real-world surveillance and pharmacovigilance workflows and a better understanding of the patient journey through analysis of social media datasets, thereby supporting evidence-informed decision-making. Although this study provides an organized framework, further efforts are needed to support real-world decision-making. Regarding future work, the hybrid analytical framework proposed in this study will undergo continuous iteration across 3 dimensions. At the data level, we will conduct cross-database, multilingual, and cross-platform comparative analyses. Methodologically, we will extend semantic-structural integration from static co-occurrence networks to dynamic networks. For validation, we will combine temporal slicing analysis and burst detection with event analysis to enhance explainability and practical utility.

## Supplementary material

10.2196/86200 Multimedia Appendix 1 Supplementary figures to support the study.

10.2196/86200 Multimedia Appendix 2 Keyword burst detection (Kleinberg bursts) and parameter robustness assessment.

10.2196/86200 Multimedia Appendix 3 Mathematical derivation and computational process documentation for the hybrid semantic-structural bibliometric analysis pipeline.

10.2196/86200 Multimedia Appendix 4 Micro-level interpretive triangulation with selected articles (evidence mapping).

10.2196/86200 Multimedia Appendix 5 List of included publications.

10.2196/86200 Multimedia Appendix 6 Supplementary tables to support the study.

10.2196/86200 Multimedia Appendix 7 Interpretation and rationale for retaining the Hierarchical Density-Based Spatial Clustering of Applications with Noise unassigned noise set (cluster 1: candidate incubator pool of peripheral cross-cutting topics in health-related social media mining).

10.2196/86200 Checklist 1 BIBLIO checklist.

10.2196/86200 Checklist 2 PRISMA-S checklist.

## Abbreviations

API: application programming interface
ARIMA: autoregressive integrated moving average
BIBLIO: Preliminary Guideline for Reporting Bibliometric Reviews of the Biomedical Literature
CH: Calinski-Harabasz
HDBSCAN: Hierarchical Density-Based Spatial Clustering of Applications with Noise
IF: impact factor
LDA: latent Dirichlet allocation
MeSH: Medical Subject Headings
ML: machine learning
NLP: natural language processing
PRISMA: Preferred Reporting Items for Systematic Reviews and Meta-Analyses
RCR: relative citation ratio
SMM: social media mining
UMAP: Uniform Manifold Approximation and Projection
