# Supplementary material for: Thematic Mapping and Evolution of Social Media Mining in Health Research: Hybrid Bibliometric Synthesis
Source: J Med Internet Res. 2026 May 8;28:e86200. doi: 10.2196/86200 (PMC13160668; doi:10.2196/86200)
Supplement: Multimedia Appendix 1 [file jmir-v28-e86200-s001.pdf]

Content of Multimedia Appendix

Multimedia Appendix 1. Supplementary figures to support the study 2

Figure S1. Resarch Workflow 2

Figure S2. End-to-end workflow for PubMed-based literature mining and strategic mapping 3

Figure S3. Author Collaboration Network in Social Media Mining Publications 4

Figure S4. Top 20 Articles Ranked by Relative Citation Ratio (RCR) and Associated Keywords 5

Figure S5. Most Cited Articles in the Citation Network of Social Media Mining Publications 6

Figure S6. Annual top 20 keywords in social media mining publications (2015–2025) 7

Figure S7. X–Y Projection of the Thematic Map of Research in Social Media Mining 8

Figure S8. X–Z Projection of the Thematic Map of Research in Social Media Mining 9

Figure S9. Y–Z Projection of the Thematic Map of Research in Social Media Mining 10

Figure S10. Inter-Cluster Coupling Heatmap 11

Figure S11. Spectral Clustering of Keywords in the 2015–2019 Time Slice (K=3) 12

Figure S12. Spectral Clustering of Keywords in the 2020–2023 Time Slice (K=8) 13

Figure S13. Spectral Clustering of Keywords in the 2024–2025 Time Slice (K=5) 14

Figure S14. Sankey Diagram of Keyword Cluster Evolution across Time Slices (2015–2025) 15

Figure S15. Relationship between keyword centrality and RCR, and distribution of RCR across clusters 15

References 16

Multimedia Appendix 1. Supplementary figures to support the study

Figure S1. Resarch Workflow

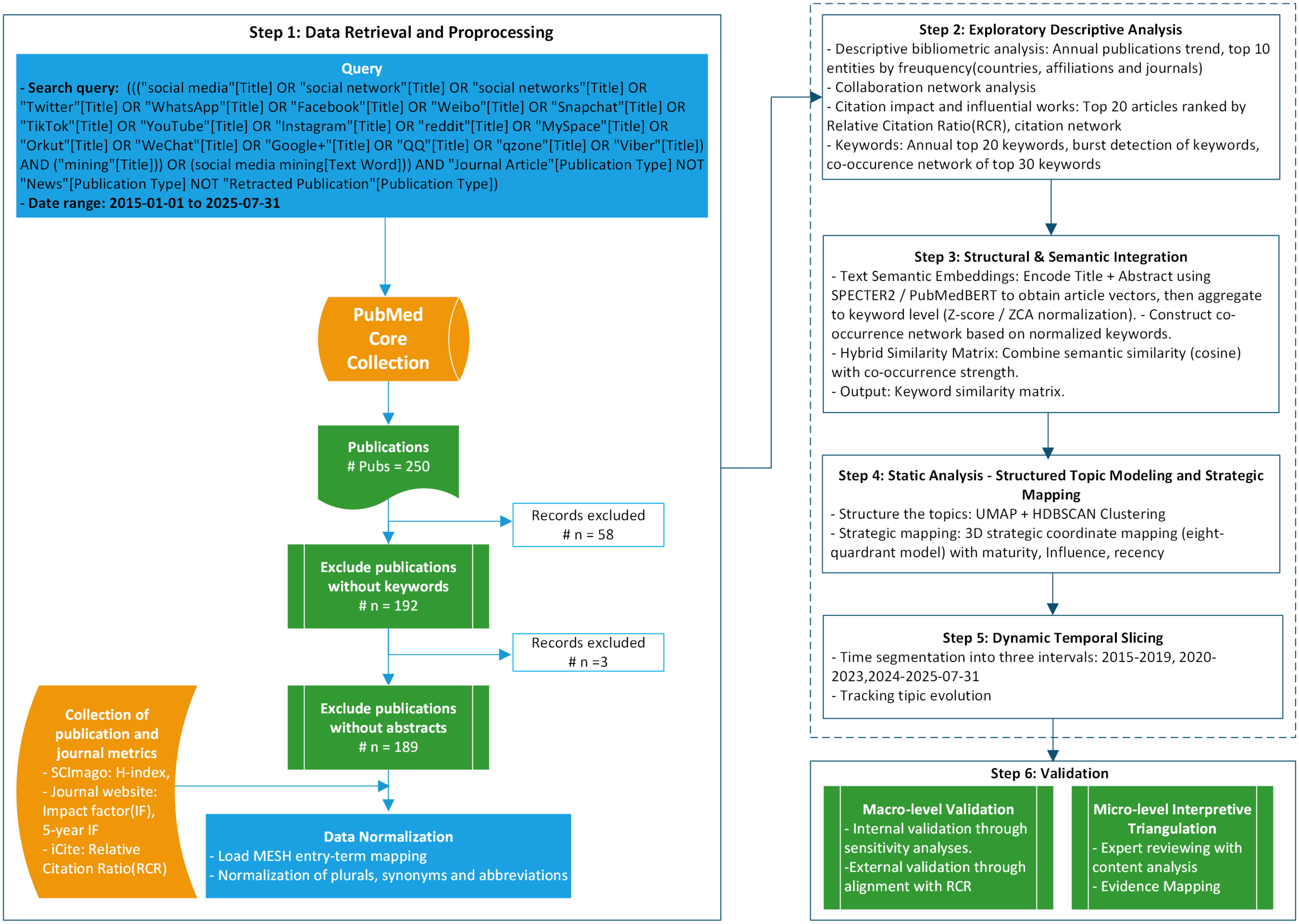

Figure S2. End-to-end workflow for PubMed-based literature mining and strategic mapping

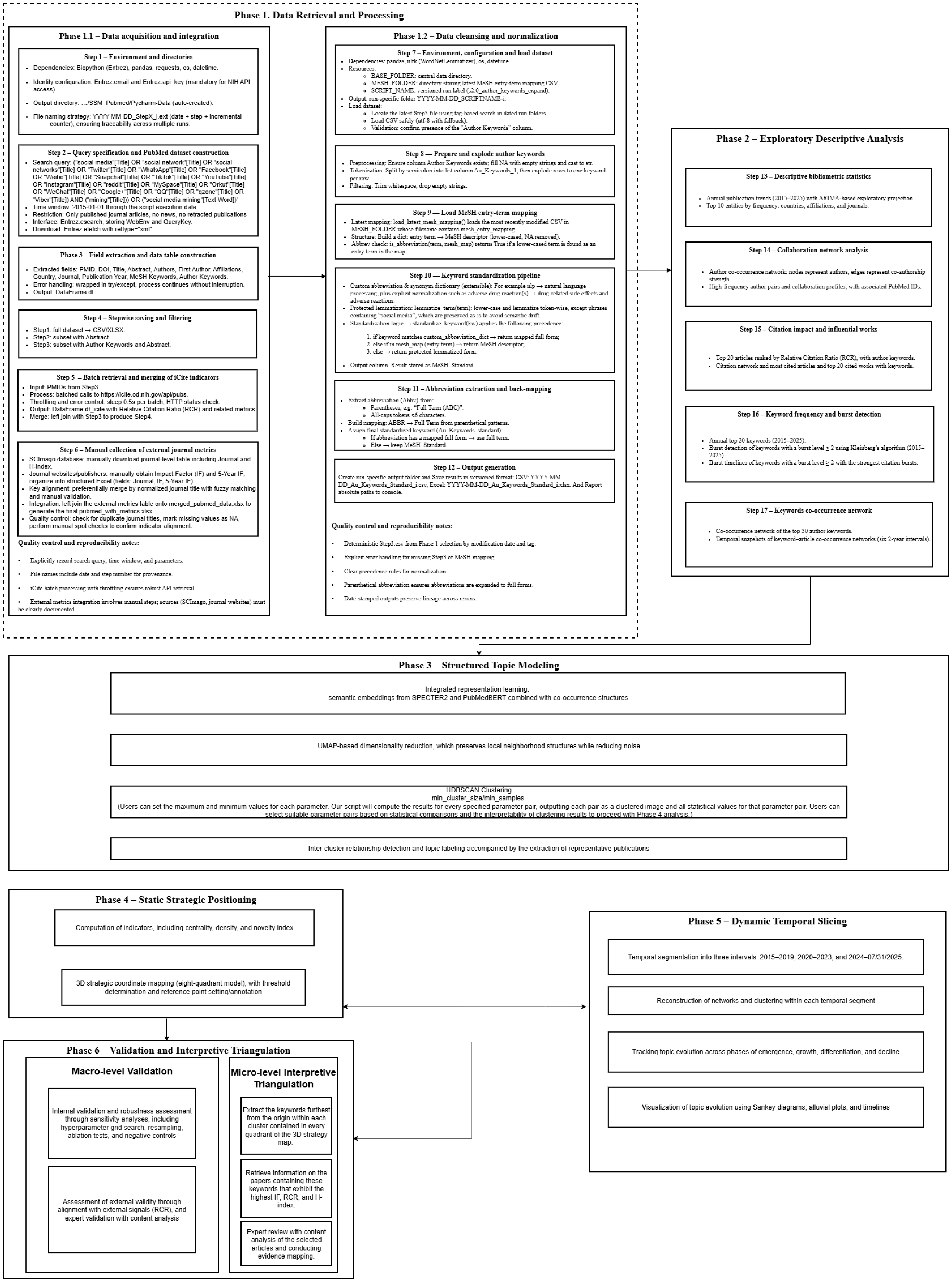

The study design integrates multi-phase procedures, from data acquisition and normalization to topic modeling, validation, and reproducibility. Phase 1–2 involve PubMed data acquisition, metadata integration, keyword standardization, and bibliometric indicator collection. Phase 3 performs exploratory descriptive analyses, including bibliometric statistics, collaboration networks, keyword bursts, and co-occurrence structures. Phase 3 further incorporates structured topic modeling, where semantic embeddings (SPECTER2/PubMedBERT) are fused with co-occurrence representations (Node2Vec), followed by UMAP dimensionality reduction, HDBSCAN clustering, and topic labeling. Phases 4–5 position the identified topics within strategic coordinate maps (2D/3D, four- or eight-quadrant models) and trace their temporal evolution across three intervals (2015–2019, 2020–2023, 2024–2025.07). Phase 6 conducts validation and robustness assessment through sensitivity analyses, hyperparameter grids, ablation, negative controls, and external benchmarking with RCR. Phase 7 delivers reproducible outputs, including experimental scripts, logs, tables, figures, and appendices.

Figure S3. Author Collaboration Network in Social Media Mining Publications

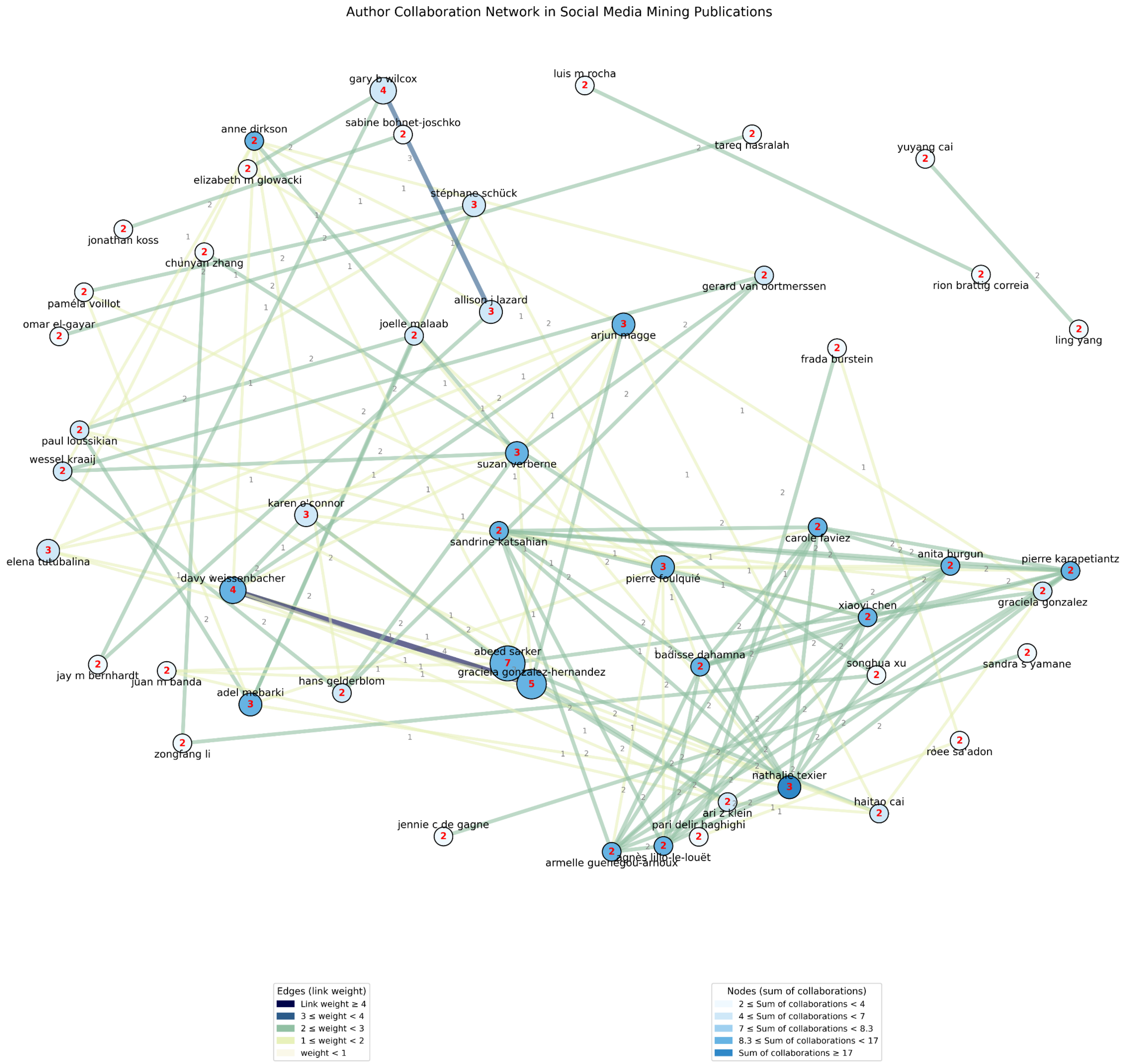

Author co-occurrence network. Nodes represent authors, with node size proportional to the number of publications by the author. Node color indicates the total number of collaborations with other authors (darker colors denote more collaborations). Edges represent co-authorship relations, with edge color and thickness reflecting the collaboration strength (number of co-occurrences), and the numeric label on each edge showing the exact co-occurrence count.

Figure S4. Top 20 Articles Ranked by Relative Citation Ratio (RCR) and Associated Keywords

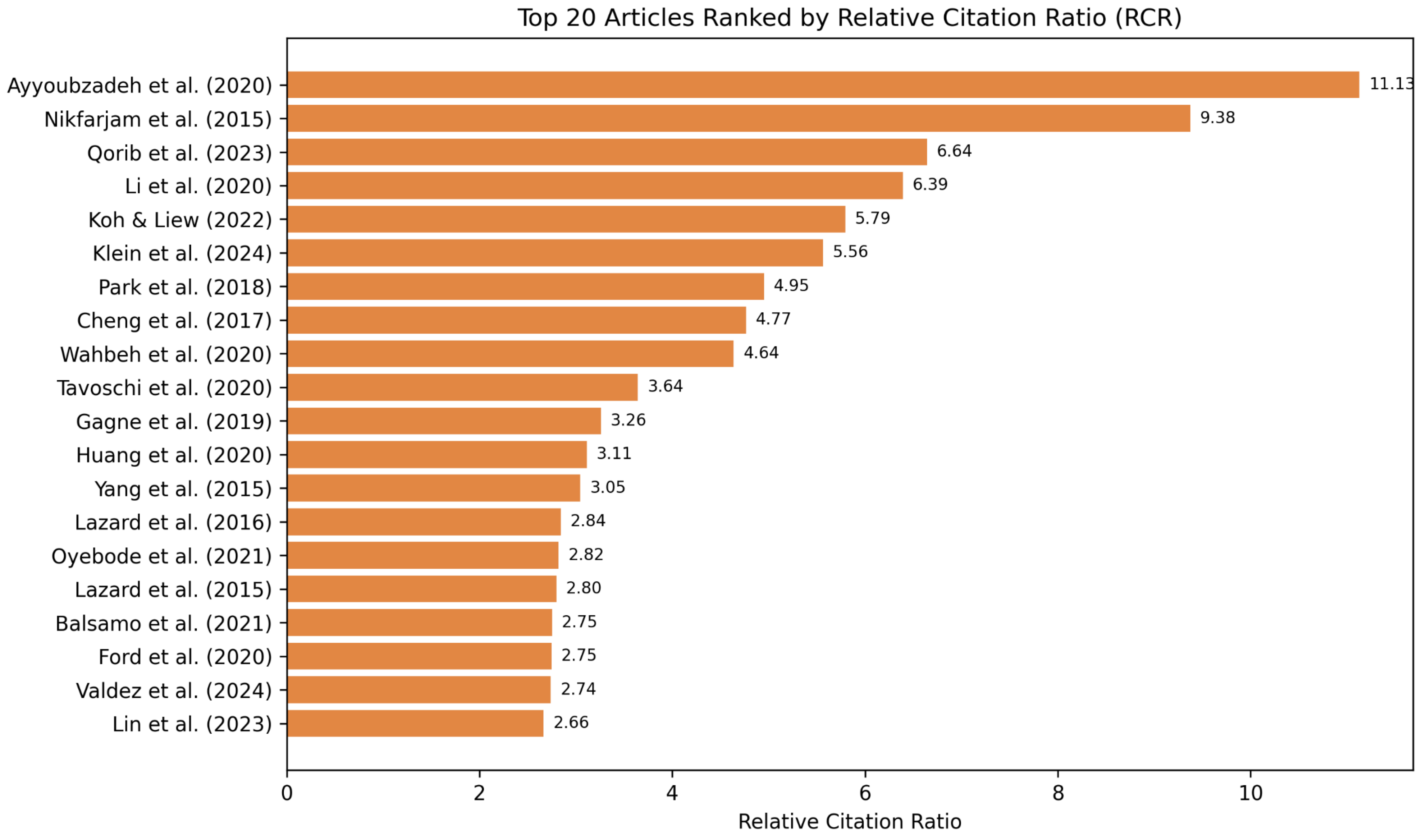

Rank	Article	Author Keywords
1	Ayyoubzadeh et al. (2020) [1]	COVID-19; Google Trends; LSTM; coronavirus; incidence; linear regression; outbreak; pandemic; prediction; public health
2	Nikfarjam et al. (2015) [2]	ADR; adverse drug reaction; deep learning word embeddings; machine learning; natural language processing; pharmacovigilance; social media mining
3	Qorib et al. (2023) [3]	Covid-19; Machine Learning; Sentiment Analysis; Twitter; Vaccine Hesitancy
4	Li et al. (2020) [4]	COVID-19; coronavirus; infectious disease; infodemiology; infoveillance; social media, surveillance
5	Koh & Liew (2022) [5]	COVID-19; Loneliness; Mental health; Natural Language Processing; Social media; Topic modeling; Twitter
6	Klein et al. (2024) [6]	data mining; machine learning; natural language processing; social media
7	Park et al. (2018) [7]	Anxiety Disorders; Consumer Health Information; Depression; Post-Traumatic; Stress Disorders; Unsupervised Machine Learning
8	Cheng et al. (2017) [8]	Chinese; machine learning; natural language; psychological stress; social media; suicide
9	Wahbeh et al. (2020) [9]	COVID-19; coronavirus; infodemiology; infoveillance; medical professionals; opinion analysis; pandemic; social media
10	Tavoschi et al. (2020) [10]	Opinion mining; Twitter; sentiment analysis; social media; vaccination; vaccine hesitancy
11	Gagne et al. (2019) [11]	Civility; Cyberincivility; Education; Incivility; Nurses; Nursing; Nursing students; Social media; Social networking sites; Twitter
12	Huang et al. (2020) [12]	COVID-19; SARS-CoV-2; Sina Weibo; coronavirus disease; help; social media
13	Yang et al. (2015) [13]	Adverse drug reactions; Latent Dirichlet Allocation (LDA); Partially supervised classification; Social media filtering; Social media mining
14	Lazard et al. (2016) [14]	Internet; e-cigarettes; social media; tweet
15	Oyebode et al. (2021) [15]	COVID-19; coronavirus; health issues; infodemiology; infoveillance; interventions; natural language processing; psychosocial issues; social issues; social media; text mining; thematic analysis
16	Lazard et al. (2015) [16]	Crisis communication; Internet; Social media; Tweet
17	Balsamo et al. (2021) [17]	Reddit; buprenorphine; drug tampering; fentanyl; heroin; opioid; oxycodone; routes of administration; social media; word embedding
18	Ford et al. (2020) [18]	ethics; health research; natural language processing; social media; text-mining
19	Valdez et al. (2024) [19]	NLP; Reddit; abortion; natural language processing; neural networks; social media
20	Lin et al. (2023) [20]	Facebook; content analysis; engagement; natural language processing; public health; smoking; social media; social media campaign; tobacco; tobacco control; topic modeling; use; youth

The bar chart presents the 20 highest-impact articles in the domain of social media mining, ranked by Relative Citation Ratio (RCR). The accompanying table lists the formatted citations alongside their author keywords and MeSH keywords, offering insight into common research topics among highly cited works.

Figure S5. Most Cited Articles in the Citation Network of Social Media Mining Publications

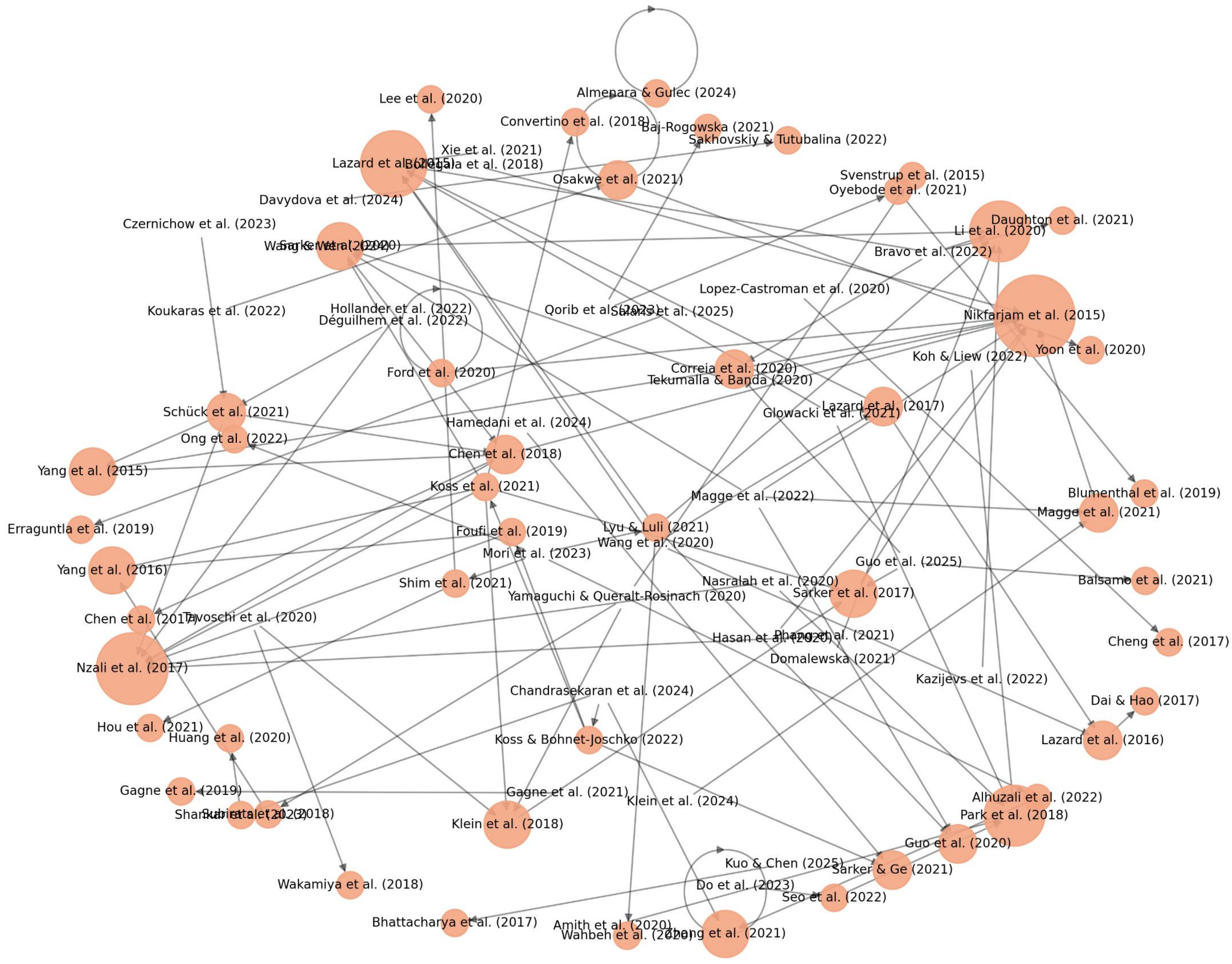

Rank	Formatted Citation	Citation Count	Author Keywords
1	Nikfarjam et al. (2015) [2]	9	ADR; adverse drug reaction; deep learning word embeddings; machine learning; natural language processing; pharmacovigilance; social media mining
2	Nzali et al. (2017) [21]	7	breast cancer; social media; text mining; unsupervised learning
3	Lazard et al. (2015) [16]	6	Crisis communication; Internet; Social media; Tweet
4	Li et al. (2020) [4]	5	COVID-19; coronavirus; infectious disease; infodemiology; infoveillance; social media, surveillance
5	Park et al. (2018) [7]	5	Anxiety Disorders; Consumer Health Information; Depression; Post-Traumatic; Stress Disorders; Unsupervised Machine Learning
6	Zhang et al. (2021) [22]	3	COVID-19; Twitter mining; concerns; disparities; infodemiology; infoveillance; pandemic; population groups; sentiments
7	Sarker et al. (2020) [23]	3	natural language processing; prescription drug misuse; social media; substance abuse detection; text mining
8	Klein et al. (2018) [24]	3	Birth defects; Cohort discovery; Epidemiology; Natural language processing; Patient-reported pregnancy outcomes; Social media mining
9	Sarker et al. (2017) [25]	3	cohort studies; data analysis; machine learning; natural language processing; pregnancy; social media; text mining
10	Yang et al. (2016) [26]	3	Health social media; Latent Dirichlet Allocation; Sentiment analysis
11	Yang et al. (2015) [13]	3	Adverse drug reactions; Latent Dirichlet Allocation (LDA); Partially supervised classification; Social media filtering; Social media mining
12	Sarker & Ge (2021) [27]	2	COVID-19; natural language processing; post-acute COVID-19 syndrome; social media; virus diseases
13	Magge et al. (2021) [28]	2	drug safety; information extraction; natural language processing; pharmacovigilance; social media mining
14	Schück et al. (2021) [29]	2	analgesic use; data mining; infodemiology; paracetamol; patient perception; pharmacovigilance; social media
15	Osakwe et al. (2021) [30]	2	COVID-19; Twitter; coronavirus; pandemic; social media
16	Guo et al. (2020) [31]	2	COVID-19; epidemiology; social media; symptoms
17	Correia et al. (2020) [32]	2	biomedicine; healthcare; pharmacovigilance; sentiment analysis; social media
18	Chen et al. (2018) [33]	2	data mining; drug misuse; drug-related side effects and adverse reactions; methylphenidate; natural language processing; pharmacovigilance; social media
19	Lazard et al. (2017) [34]	2	Electronic nicotine delivery devices; Media; Public opinion

20	Lazard et al. (2016) [14]	2	Internet; e-cigarettes; social media; tweet

Directed citation network among articles related to social media mining, showing the most interconnected subset. Each node represents a publication; node size is proportional to the number of times the article is cited by other articles within the network. The accompanying table lists the 20 most cited articles within the network, including their formatted citation, citation count, and author keywords. This highlights influential contributions and recurring research themes.

Figure S6. Annual top 20 keywords in social media mining publications (2015–2025)

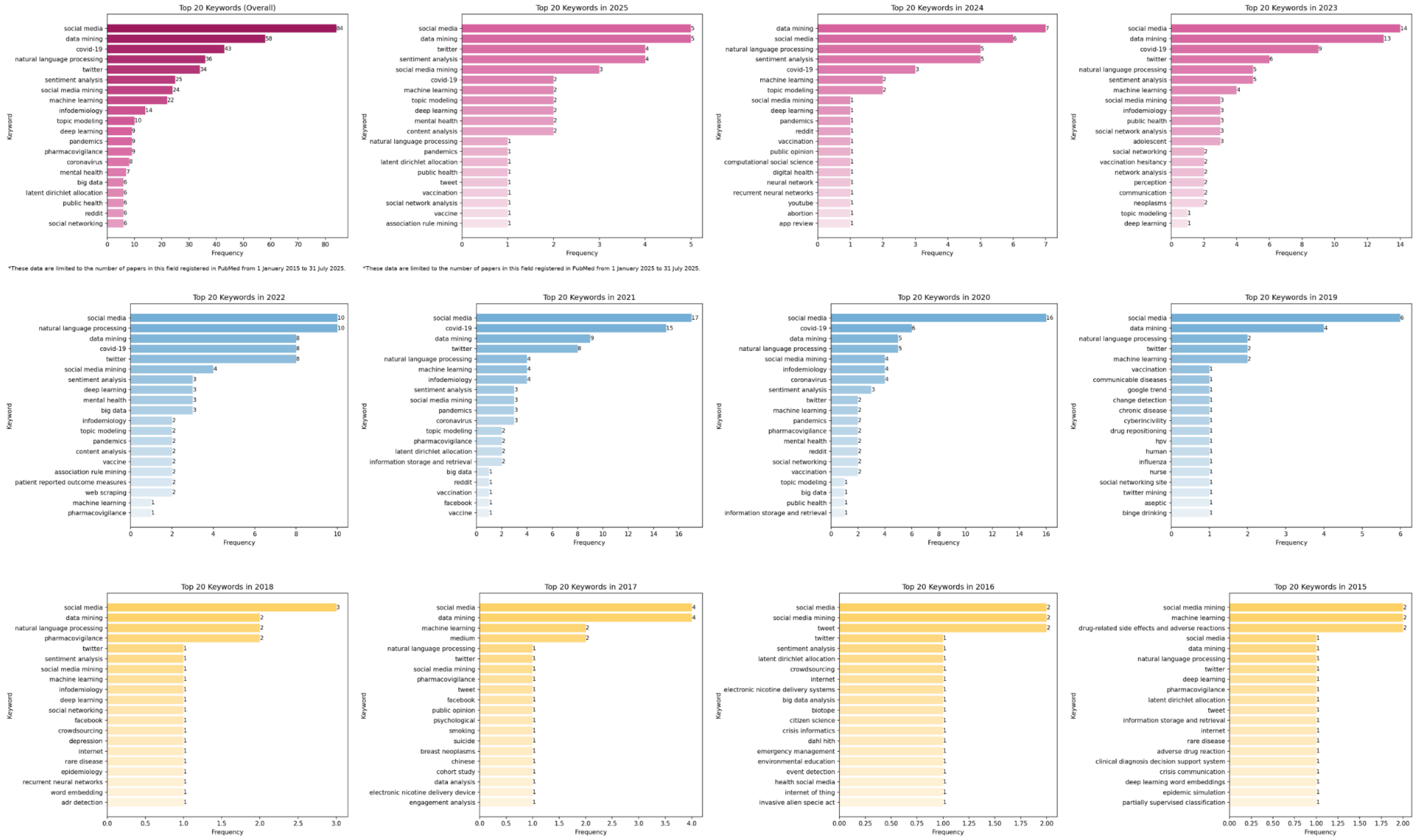

Figure S7. X–Y Projection of the Thematic Map of Research in Social Media Mining

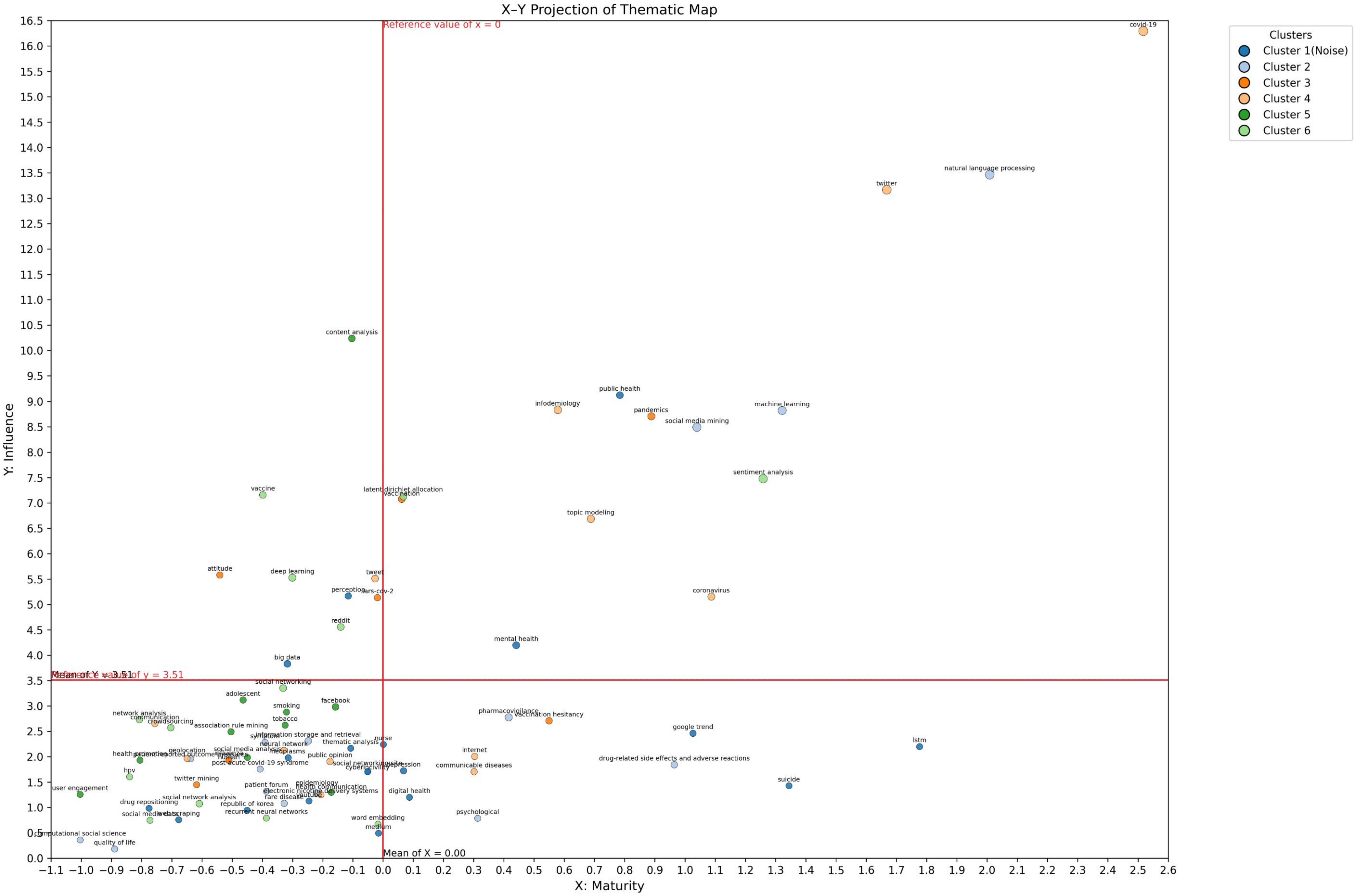

The X-axis represents Maturity, while the Y-axis represents Influence. Each node corresponds to a keyword, with node size proportional to its frequency and node color indicating cluster membership. Dashed lines indicate the mean values of X and Y, while red lines show the reference values (x = 0, y = 3.51).

Figure S8. X–Z Projection of the Thematic Map of Research in Social Media Mining

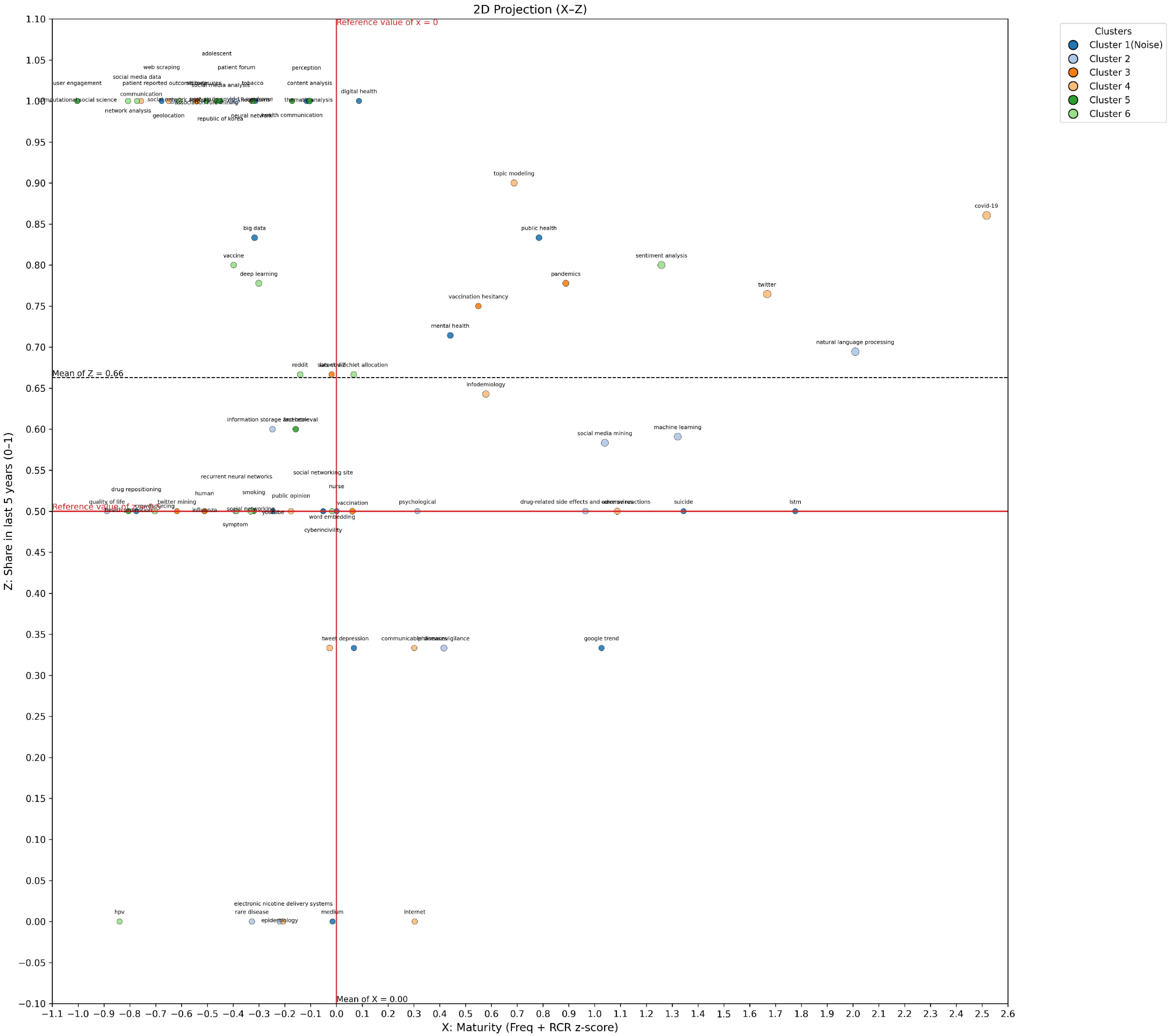

The X-axis represents Maturity, while the Z-axis represents Recency. Each node corresponds to a keyword, with node size proportional to its frequency and node color indicating cluster membership. Dashed lines indicate the mean values of X and Z, while red lines show the reference values (x = 0, z = 0.5).

Figure S9. Y–Z Projection of the Thematic Map of Research in Social Media Mining

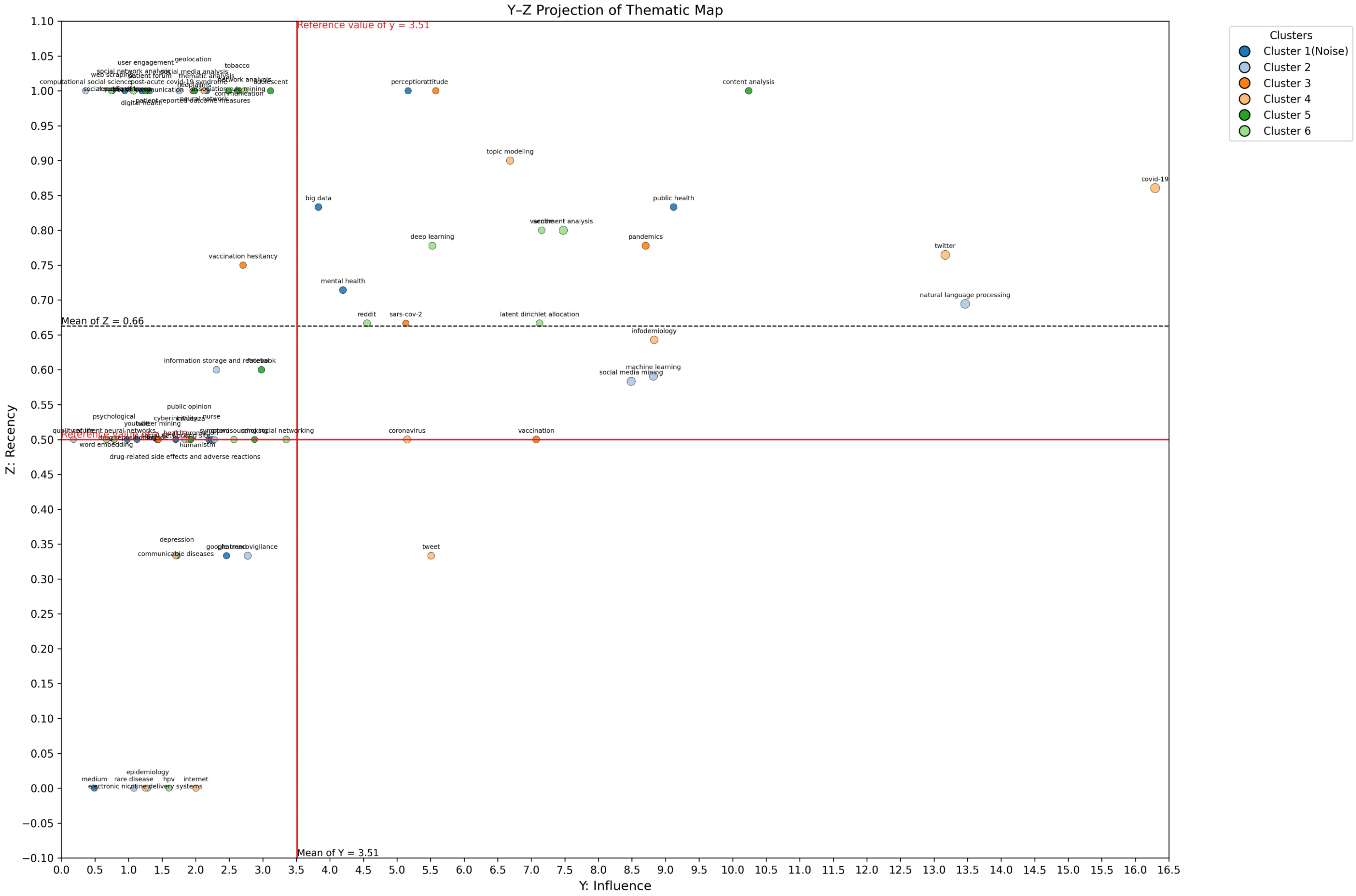

The Y-axis represents Influence, while the Z-axis represents Recency. Each node corresponds to a keyword, with node size proportional to its frequency and node color indicating cluster membership. Dashed lines indicate the mean values of Y and Z, while red lines show the reference values (y = 3.51, z = 0.5).

Figure S10. Inter-Cluster Coupling Heatmap

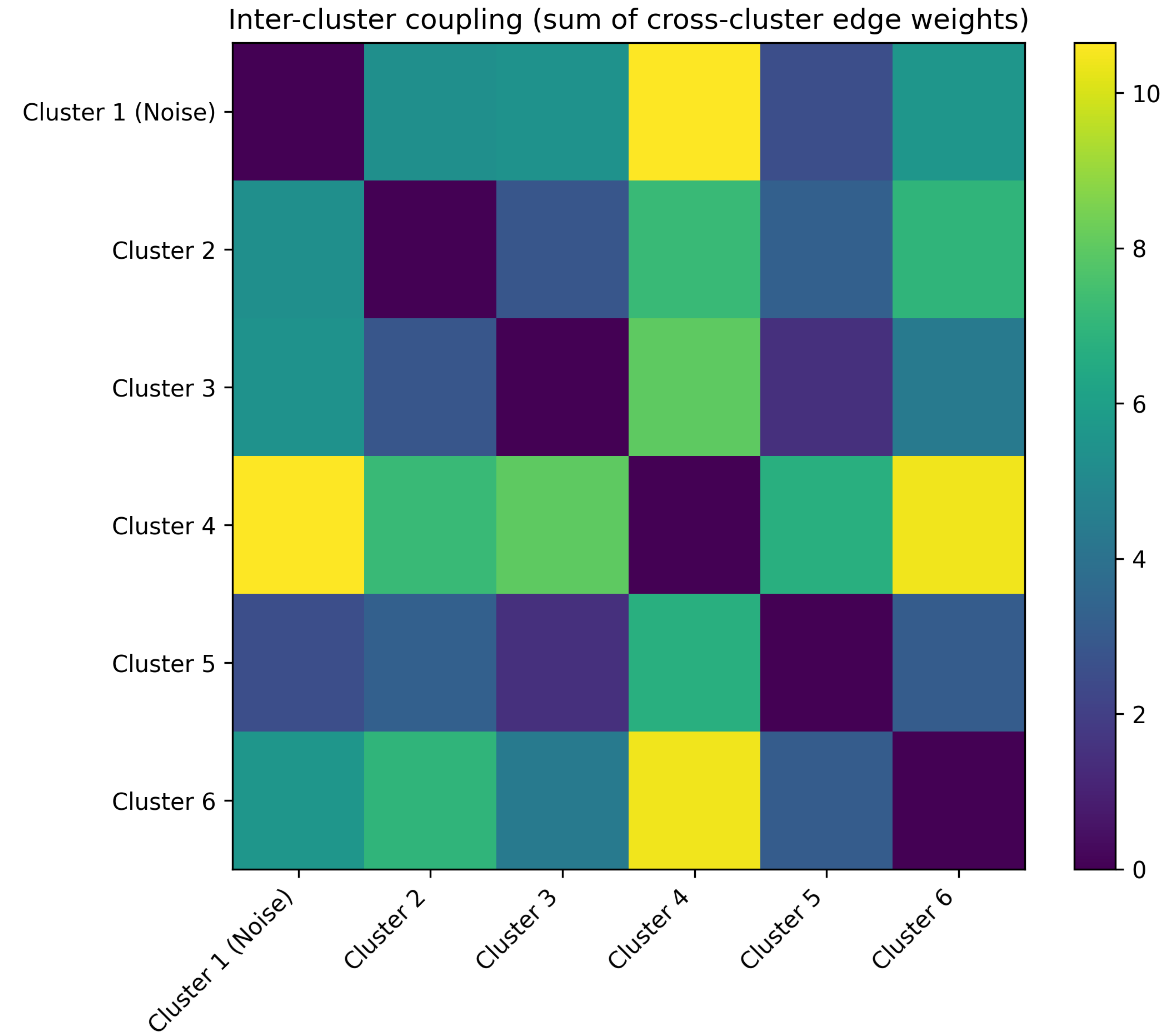

This figure illustrates the cross-cluster coupling strength between each pair of thematic clusters, represented as the sum of cross-cluster edge weights. Brighter colors indicate stronger inter-cluster overlap. The diagonal values are 0. Detailed numerical values are shown in Supplementary Table 4 in Multimedia Appendix 6.

Figure S11. Spectral Clustering of Keywords in the 2015–2019 Time Slice (K=3)

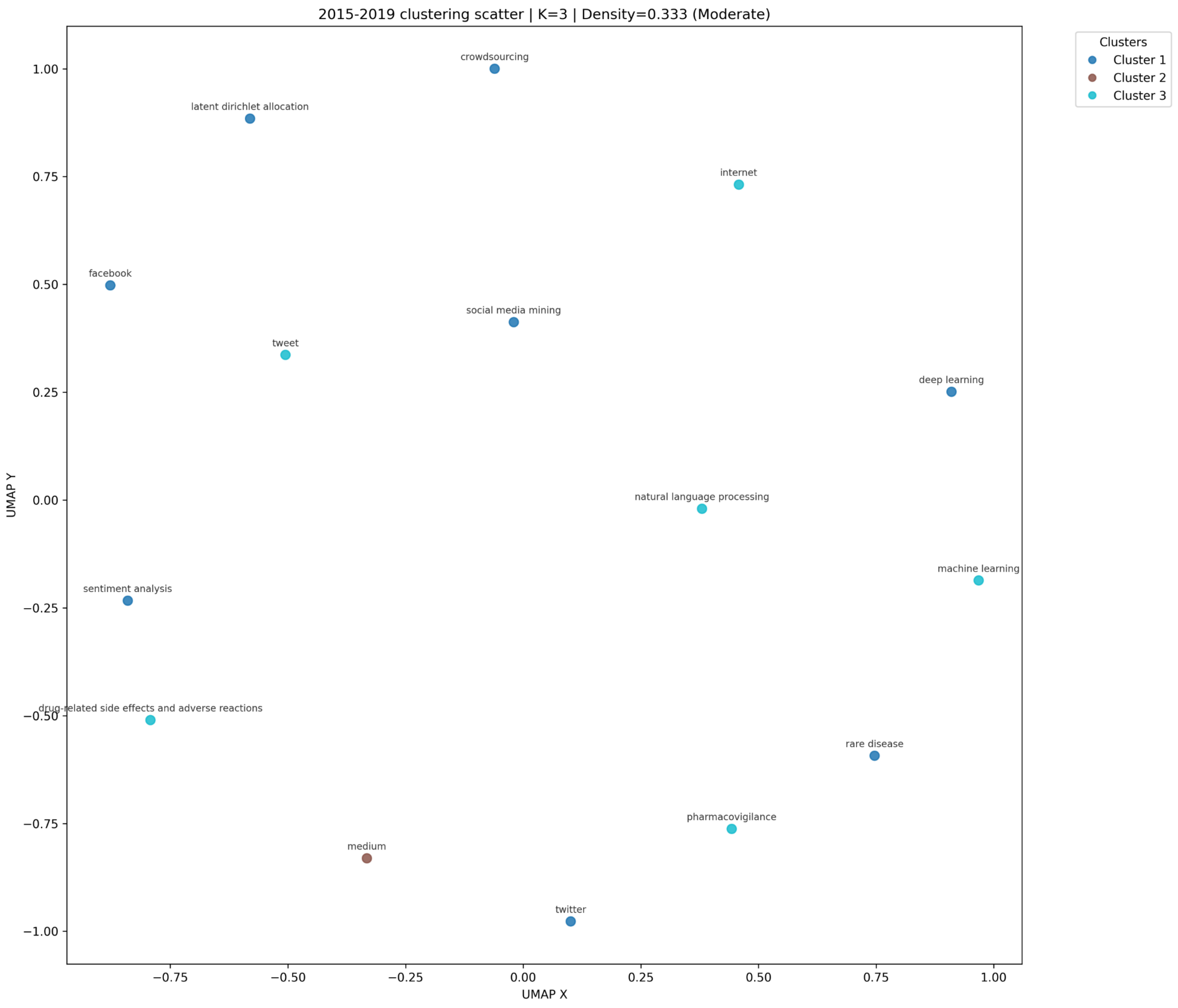

This figure presents the results of spectral clustering applied to the 2015–2019 time slice of the corpus. Keywords are embedded into a two-dimensional UMAP space and grouped into three clusters based on their co-occurrence structure. Each point represents a keyword, with proximity indicating higher similarity in usage context. Colors denote spectral cluster assignments, showing three thematic areas: methodological approaches (e.g., deep learning, latent Dirichlet allocation), social media platforms (Twitter, Facebook, Medium), and applied domains such as pharmacovigilance and rare disease. The clustering density of 0.333 indicates a moderate degree of separation between clusters.

Figure S12. Spectral Clustering of Keywords in the 2020–2023 Time Slice (K=8)

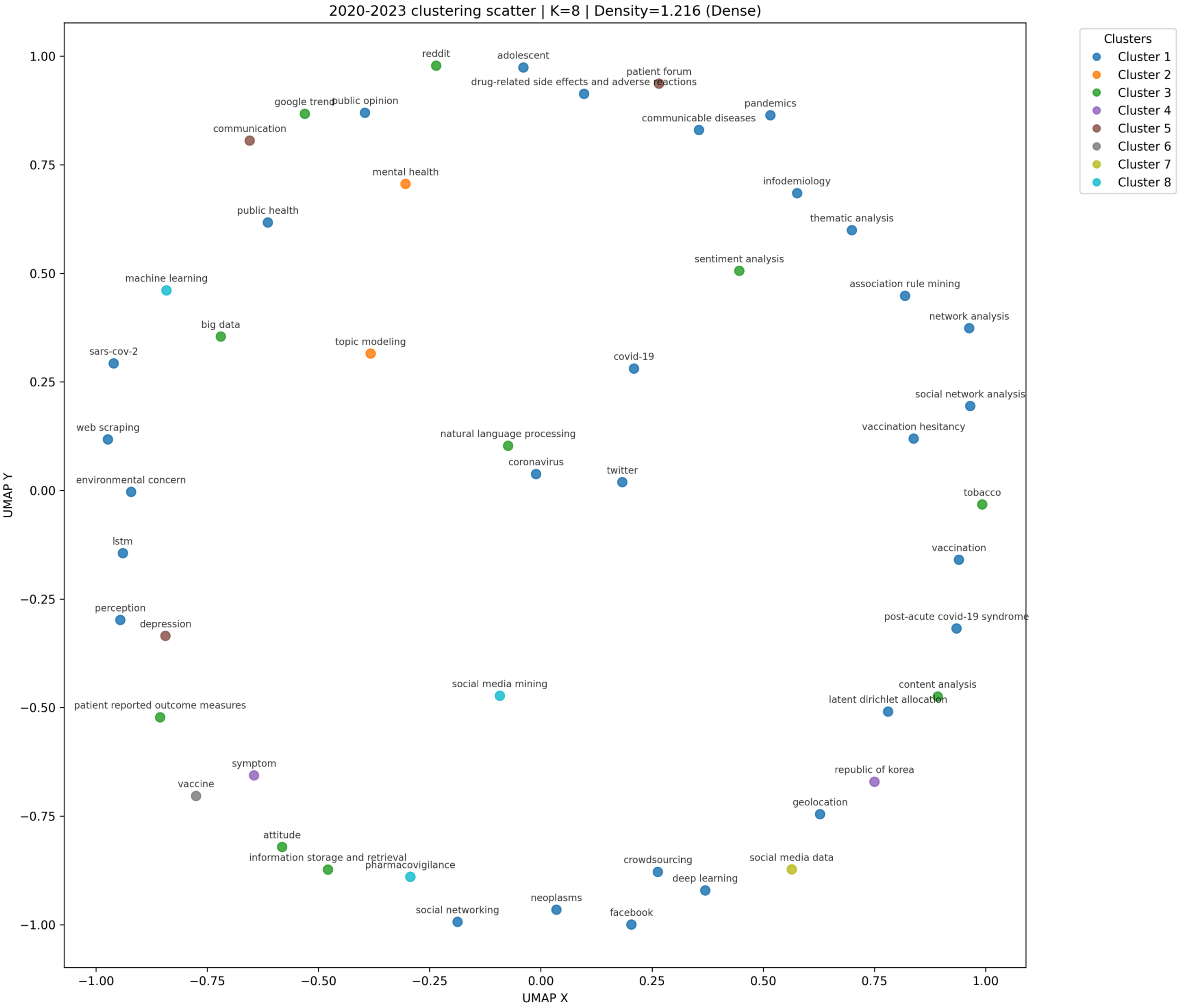

This figure illustrates the spectral clustering results for the 2020–2023 time slice of the corpus. Keywords are embedded into a two-dimensional UMAP space and grouped into eight clusters based on co-occurrence patterns. Each point represents a keyword, with spatial proximity indicating similarity in research usage, and colors denoting distinct clusters. The clusters capture several thematic areas, including pandemic-related terms (COVID-19, SARS-CoV-2, vaccination, vaccination hesitancy), methodological approaches (deep learning, latent Dirichlet allocation, topic modeling), social media platforms (Twitter, Facebook, Reddit), and applied domains such as pharmacovigilance and patient-reported outcome measures. The clustering density (1.216) indicates a relatively dense solution, reflecting the strong interconnections among topics during the COVID-19 period.

Figure S13. Spectral Clustering of Keywords in the 2024–2025 Time Slice (K=5)

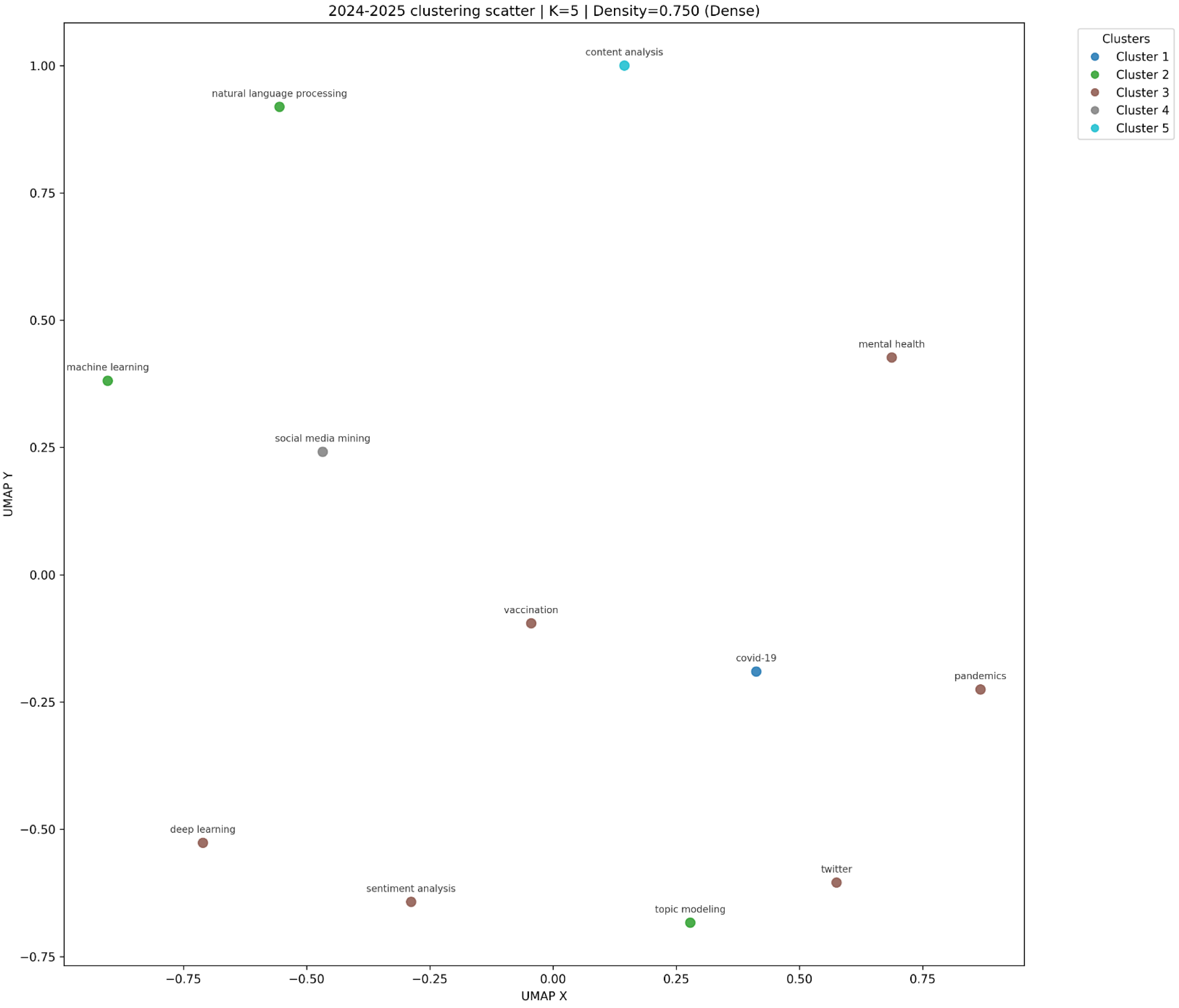

This figure displays the spectral clustering results for the 2024–2025 time slice of the corpus. Keywords are projected into a two-dimensional UMAP space and grouped into five clusters. Each dot represents a keyword, with spatial proximity reflecting similarity in usage and colors indicating cluster membership. The clusters capture key themes of the immediate post-pandemic period: methodological terms (machine learning, deep learning, topic modeling), public health and pandemic-related concepts (COVID-19, vaccination, pandemics), mental health research (mental health, sentiment analysis), social media platforms (Twitter, social media mining), and information/communication methods (content analysis, natural language processing). The clustering density (0.750) suggests a moderately dense configuration, reflecting the consolidation of research foci after the peak of the COVID-19 literature surge.

Figure S14. Sankey Diagram of Keyword Cluster Evolution across Time Slices (2015–2025)

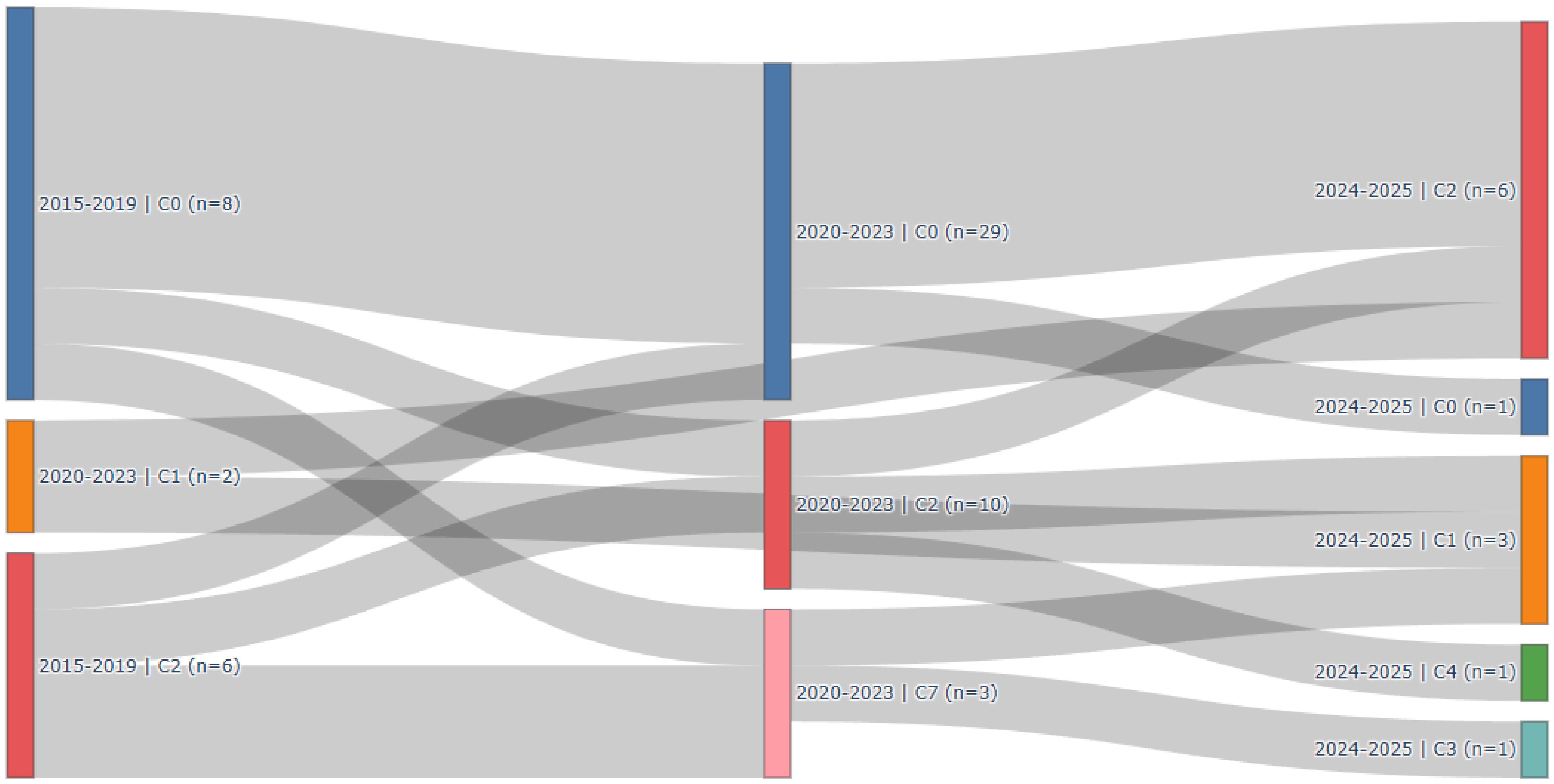

This Sankey diagram visualizes the evolution of keyword clusters across three time slices (2015–2019, 2020–2023, and 2024–2025). Each node represents a cluster identified in a specific time period, with its size proportional to the number of keywords (n). The flows (links) indicate how clusters evolve over time, capturing the continuity, divergence, and merging of research themes. For example, the major cluster from 2015–2019 (C0) expands substantially in 2020–2023 (C0, n=29) and continues into 2024–2025 (C2, n=6), while other clusters such as C2 (2015–2019) split into multiple branches in the subsequent periods. The diagram highlights both the persistence of dominant themes and the reorganization of emerging topics over time.

Figure S15. Relationship between keyword centrality and RCR, and distribution of RCR across clusters

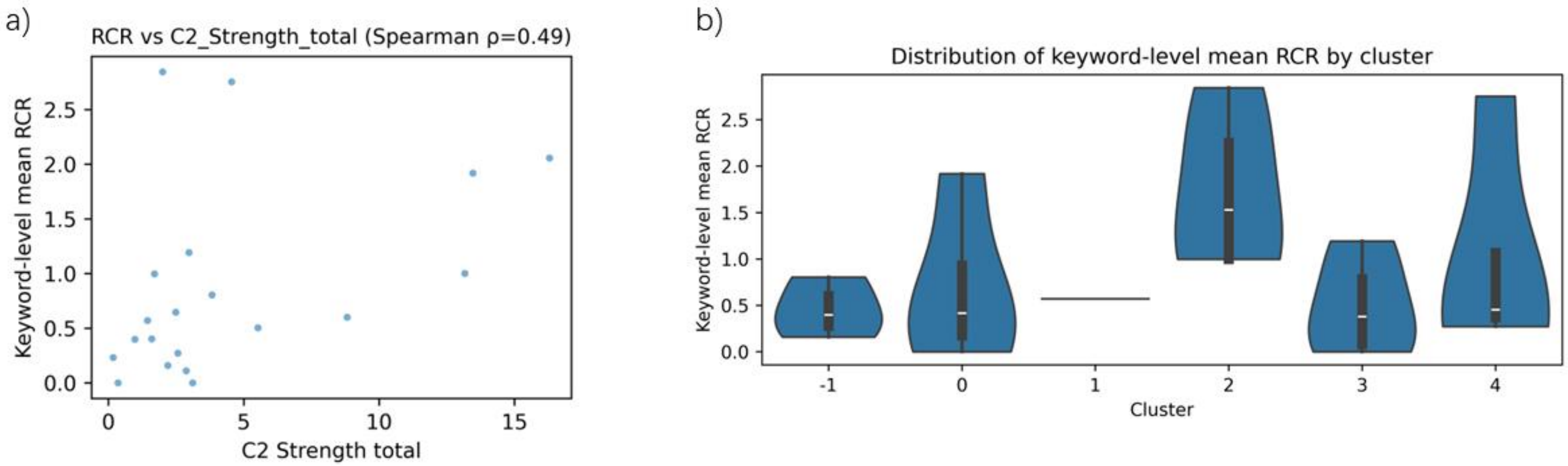

(a) Relationship between keyword strength (Strength_total) and mean RCR (Spearman ρ=0.49, p<0.05), showing that more central keywords in the network are associated with higher citation impact.

(b) Distribution of mean RCR across clusters (violin plots). Cluster 2 exhibited the highest citation impact (median ≈ 1.5, maximum approaching 3), Clusters 0 and 3 showed lower impact (~0.5–0.7), and noise points (Cluster -1) had the lowest mean RCR (~0.45). These findings indicate that the clustering not only reveals internal structural differences but is also validated by external citation metrics.

References

1. Ayyoubzadeh SM, Ayyoubzadeh SM, Zahedi H, Ahmadi M, R Niakan Kalhori S. Predicting COVID-19 Incidence Through Analysis of Google Trends Data in Iran: Data Mining and Deep Learning Pilot Study. JMIR Public Health Surveill 2020;6(2):e18828. PMID:32234709
2. Nikfarjam A, Sarker A, O'Connor K, Ginn R, Gonzalez G. Pharmacovigilance from social media: mining adverse drug reaction mentions using sequence labeling with word embedding cluster features. J Am Med Inform Assoc 2015;22(3):671-681. PMID:25755127
3. Qorib M, Oladunni T, Denis M, Ososanya E, Cotae P. Covid-19 vaccine hesitancy: Text mining, sentiment analysis and machine learning on COVID-19 vaccination Twitter dataset. Expert Syst Appl 2023;212:118715. PMID:36092862
4. Li J, Xu Q, Cuomo R, Purushothaman V, Mackey T. Data Mining and Content Analysis of the Chinese Social Media Platform Weibo During the Early COVID-19 Outbreak: Retrospective Observational Infoveillance Study. JMIR Public Health Surveill 2020;6(2):e18700. PMID:32293582
5. Koh JX, Liew TM. How loneliness is talked about in social media during COVID-19 pandemic: Text mining of 4,492 Twitter feeds. J Psychiatr Res 2022;145:317-324. PMID:33190839
6. Klein AZ, Banda JM, Guo Y, Schmidt AL, Xu D, Flores Amaro I, Rodriguez-Esteban R, Sarker A, Gonzalez-Hernandez G. Overview of the 8th Social Media Mining for Health Applications (#SMM4H) shared tasks at the AMIA 2023 Annual Symposium. J Am Med Inform Assoc 2024;31(4):991-996. PMID:38218723
7. Park A, Conway M, Chen AT. Examining Thematic Similarity, Difference, and Membership in Three Online Mental Health Communities from Reddit: A Text Mining and Visualization Approach. Comput Human Behav 2018;78:98-112. PMID:29456286
8. Cheng Q, Li TM, Kwok C-L, Zhu T, Yip PS. Assessing Suicide Risk and Emotional Distress in Chinese Social Media: A Text Mining and Machine Learning Study. J Med Internet Res 2017;19(7):e243. PMID:28694239
9. Wahbeh A, Nasralah T, Al-Ramahi M, El-Gayar O. Mining Physicians' Opinions on Social Media to Obtain Insights Into COVID-19: Mixed Methods Analysis. JMIR Public Health Surveill 2020;6(2):e19276. PMID:32421686
10. Tavoschi L, Quattrone F, D'Andrea E, Ducange P, Vabanesi M, Marcelloni F, Lopalco PL. Twitter as a sentinel tool to monitor public opinion on vaccination: an opinion mining analysis from September 2016 to August 2017 in Italy. Hum Vaccin Immunother 2020;16(5):1062-1069. PMID:32118519
11. Gagne JC de, Cho E, Yamane SS, Jin H, Nam JD, Jung D. Analysis of Cyberincivility in Posts by Health Professions Students: Descriptive Twitter Data Mining Study. JMIR Med Educ 2021;7(2):e28805. PMID:33983129
12. Huang C, Xu X, Cai Y, Ge Q, Zeng G, Li X, Zhang W, Ji C, Yang L. Mining the Characteristics of COVID-19 Patients in China: Analysis of Social Media Posts. J Med Internet Res 2020;22(5):e19087. PMID:32401210
13. Yang M, Kiang M, Shang W. Filtering big data from social media--Building an early warning system for adverse drug reactions. J Biomed Inform 2015;54:230-240. PMID:25688695
14. Lazard AJ, Saffer AJ, Wilcox GB, Chung AD, Mackert MS, Bernhardt JM. E-Cigarette Social Media Messages: A Text Mining Analysis of Marketing and Consumer Conversations on Twitter. JMIR Public Health Surveill 2016;2(2):e171. PMID:27956376
15. Oyebode O, Ndulue C, Adib A, Mulchandani D, Suruliraj B, Orji FA, Chambers CT, Meier S, Orji R. Health, Psychosocial, and Social Issues Emanating From the COVID-19 Pandemic Based on Social Media Comments: Text Mining and Thematic Analysis Approach. JMIR Med Inform 2021;9(4):e22734. PMID:33684052
16. Lazard AJ, Scheinfeld E, Bernhardt JM, Wilcox GB, Suran M. Detecting themes of public concern: a text mining analysis of the Centers for Disease Control and Prevention's Ebola live Twitter chat. Am J Infect Control 2015;43(10):1109-1111. PMID:26138998
17. Balsamo D, Bajardi P, Salomone A, Schifanella R. Patterns of Routes of Administration and Drug Tampering for Nonmedical Opioid Consumption: Data Mining and Content Analysis of Reddit Discussions. J Med Internet Res 2021;23(1):e21212. PMID:33393910
18. Ford E, Shepherd S, Jones K, Hassan L. Toward an Ethical Framework for the Text Mining of Social Media for Health Research: A Systematic Review. Front Digit Health 2020;2:592237. PMID:34713062
19. Valdez D, Mena-Meléndez L, Crawford BL, Jozkowski KN. Analyzing Reddit Forums Specific to Abortion That Yield Diverse Dialogues Pertaining to Medical Information Seeking and Personal Worldviews: Data Mining and Natural Language Processing Comparative Study. J Med Internet Res 2024;26:e47408. PMID:38354044
20. Lin S-Y, Cheng X, Zhang J, Yannam JS, Barnes AJ, Koch JR, Hayes R, Gimm G, Zhao X, Purohit H, Xue H. Social Media Data Mining of Antitobacco Campaign Messages: Machine Learning Analysis of Facebook Posts. J Med Internet Res 2023;25:e42863. PMID:36780224
21. Tapi Nzali MD, Bringay S, Lavergne C, Mollevi C, Opitz T. What Patients Can Tell Us: Topic Analysis for Social Media on Breast Cancer. JMIR Med Inform 2017;5(3):e23. PMID:28760725
22. Zhang C, Xu S, Li Z, Hu S. Understanding Concerns, Sentiments, and Disparities Among Population Groups During the COVID-19 Pandemic Via Twitter Data Mining: Large-scale Cross-sectional Study. J Med Internet Res 2021;23(3):e26482. PMID:33617460
23. Sarker A, DeRoos A, Perrone J. Mining social media for prescription medication abuse monitoring: a review and proposal for a data-centric framework. J Am Med Inform Assoc 2020;27(2):315-329. PMID:31584645
24. Klein AZ, Sarker A, Cai H, Weissenbacher D, Gonzalez-Hernandez G. Social media mining for birth defects research: A rule-based, bootstrapping approach to collecting data for rare health-related events on Twitter. J Biomed Inform 2018;87:68-78. PMID:30292855
25. Sarker A, Chandrashekar P, Magge A, Cai H, Klein A, Gonzalez G. Discovering Cohorts of Pregnant Women From Social Media for Safety Surveillance and Analysis. J Med Internet Res 2017;19(10):e361. PMID:29084707
26. Yang FC, Lee AJ, Kuo SC. Mining Health Social Media with Sentiment Analysis. Journal of medical systems 2016;40(11). PMID:27663246
27. Sarker A, Ge Y. Mining long-COVID symptoms from Reddit: characterizing post-COVID syndrome from patient reports. JAMIA Open 2021;4(3):ooab075. PMID:34485849
28. Magge A, Tutubalina E, Miftahutdinov Z, Alimova I, Dirkson A, Verberne S, Weissenbacher D, Gonzalez-Hernandez G. DeepADEMiner: a deep learning pharmacovigilance pipeline for extraction and normalization of adverse drug event mentions on Twitter. J Am Med Inform Assoc 2021;28(10):2184-2192. PMID:34270701
29. Schück S, Roustamal A, Gedik A, Voillot P, Foulquié P, Penfornis C, Job B. Assessing Patient Perceptions and Experiences of Paracetamol in France: Infodemiology Study Using Social Media Data Mining. J Med Internet Res 2021;23(7):e25049. PMID:34255645
30. Osakwe ZT, Ikhapoh I, Arora BK, Bubu OM. Identifying public concerns and reactions during the COVID-19 pandemic on Twitter: A text-mining analysis. Public Health Nurs 2021;38(2):145-151. PMID:33258149
31. Guo J-W, Radloff CL, Wawrzynski SE, Cloyes KG. Mining twitter to explore the emergence of COVID-19 symptoms. Public Health Nurs 2020;37(6):934-940. PMID:32937679
32. Correia RB, Wood IB, Bollen J, Rocha LM. Mining Social Media Data for Biomedical Signals and Health-Related Behavior. Annu Rev Biomed Data Sci 2020;3:433-458. PMID:32550337
33. Chen X, Faviez C, Schuck S, Lillo-Le-Louët A, Texier N, Dahamna B, Huot C, Foulquié P, Pereira S, Leroux V, Karapetiantz P, Guenegou-Arnoux A, Katsahian S, Bousquet C, Burgun A. Mining Patients' Narratives in Social Media for Pharmacovigilance: Adverse Effects and Misuse of Methylphenidate. Front Pharmacol 2018;9:541. PMID:29881351
34. Lazard AJ, Wilcox GB, Tuttle HM, Glowacki EM, Pikowski J. Public reactions to e-cigarette regulations on Twitter: a text mining analysis. Tob Control 2017;26(e2):e112-e116. PMID:28341768
