# Supplementary material for: Thematic Mapping and Evolution of Social Media Mining in Health Research: Hybrid Bibliometric Synthesis
Source: J Med Internet Res. 2026 May 8;28:e86200. doi: 10.2196/86200 (PMC13160668; doi:10.2196/86200)
Supplement: Multimedia Appendix 2 [file jmir-v28-e86200-s002.pdf]

Multimedia Appendix 2. Keyword burst detection (Kleinberg bursts) and parameter robustness assessment

To systematically identify bursts of attention towards research topics over time, we applied Kleinberg's burst detection algorithm to a long-format table of standardized keywords and publication years after completing keyword cleaning and standardization. The implementation was carried out in R, primarily utilizing packages such as *bursts, dplyr, tidyr, stringr, openxlsx, and data.table*. The raw input was an Excel file aggregated from PubMed and manually/rule-based standardized, containing at least two columns: keywords_standard and Publication Year. The script automatically performed reading, preprocessing, batch parameter grid search, result export for each parameter set, robustness and realism metrics calculation, comprehensive scoring and ranking, as well as graphical visualization.

1) Data restructuring and time-slice construction

The literature-level keyword-year records were transformed into keyword-level time-count sequences. The specific procedure was as follows: First, the standardized keywords for each article were split by semicolons, and null values and whitespace were removed (using separate_rows and str_trim). Then, using a predefined time slice width ω (in years) , natural years were mapped to consecutive slice indices using the formula: $slice = \lfloor (year - start\_year)/\omega \rfloor + 1$. For each keyword, the occurrence count $N_{k,s}$, within each slice was calculated. All missing slices were explicitly filled with zero counts to generate a complete keyword-time slice count matrix and its long-format representation. This step ensured that all keyword time series were of consistent length, making them comparable and suitable for modeling.

2) Event offsets and Kleinberg algorithm

The Kleinberg model treats observed "events" as a point process arriving over time. In the discrete count scenario, we converted the integer count $N_{k,s}$ of each time slice into several "arrival times." These times were positioned at the index of the respective slice with minimal jitter added, forming a monotonically increasing array of time offsets $\{t_i\}$. This array was then passed to the kleinberg(offsets, s, gamma) function, where s > 1 is the state magnification base (fixed at *s = 2* in the script) and γ > 0 is the "penalty coefficient" for state transitions (a larger value indicates less frequent switching to high-intensity states). The algorithm outputs the start and end times of each burst interval along with its corresponding level. We retained intervals with level ⩾ 2 as significant bursts (the motivation for setting a Level threshold is to exclude the weakest random fluctuations) and mapped them back to natural years using the time slice width ω, obtaining the start/end slices, duration in slices (duration_slices), duration in years (duration_years), absolute start/end years

(start_year_abs/end_year_abs), etc., for each burst. To avoid fragmented noise, each burst was required to have a minimum length of at least min_len_slices (parameter L).

3) Parameter Grid and "Burst Strength Index"

Considering the sensitivity of the Kleinberg model to γ, time slice width ω, and minimum burst length L, we executed batch runs over a three-dimensional grid: γ ∈ {0.5,1,2,5}, w ∈ {1,2,3} years, L ∈ {1,2,3} slices, resulting in 36 parameter combinations (each identified as G{γ}_W{ω}_L{L}, e.g., "G0.5_W3_L1"). For each burst b of each keyword k, we defined the Burst Strength Index as: $Strength\_index_{k,b} = level_{k,b} \times duration\_years_{k,b}$. This index comprehensively reflects the combined contribution of burst level and duration. Aggregates such as sum_strength and mean_level at the keyword level (obtained by summation or averaging) facilitate comparisons across parameter sets and keywords.

4) Keyword-level explanatory, discriminability, and realism metrics

Beyond burst strength, we evaluated three types of properties at the keyword level for parameter selection and result interpretation:

Explainability / Coverage: For a keyword k, let C_k be the union of all time slices covered by its burst intervals. The coverage is defined as coverage: $coverage_k = \sum_{s \in C_k} N_{k,s} \,/\, \sum_s N_{k,s}$. This ratio describes the proportion of total occurrences explained by the "burst periods." A higher value indicates that the burst intervals account for a larger share of the observed frequency.

Discriminability: For a given parameter set, the probability distribution of the sum_strength across all keywords is calculated: $p_k = \frac{sum\_strength_k}{sum\_strength_,}$. The entropy: $H = -\sum_k p_k \log p_k$ is then computed and normalized to$1 - H \,/\, \log K$ (where K is the number of keywords with bursts). A higher value indicates that burst strength is more concentrated in fewer keywords, implying greater discriminability; conversely, a lower value indicates more dispersion.

Realism: We provided two definitions and summarized them using the median. The default scheme (median_ratio) calculates the ratio of "mean within-burst intensity" to "mean pre-burst window intensity" for each burst interval *b* = *[a, b]*, and then takes the median of these ratios at the keyword level. The alternative scheme (lift_overall) compares the "mean within-burst intensity" to the "overall mean intensity." The pre-burst window length was set to pre_window_slices = 2 (i.e., 2 slices) in the script to mitigate the impact of short-term seasonal fluctuations.

5) Stability Metrics and Sensitivity Analysis

Stability measures the consistency of the set of bursting keywords across different parameter sets. We used two types of metrics, selecting one for the subsequent comprehensive scoring (consensus Jaccard by default):

Consensus Set Jaccard: If a keyword was detected as bursting in at least half of the parameter sets (threshold: 50% of the number of sets, rounded up), it was included in the "consensus set." For any

parameter set ρ, the Jaccard coefficient between its set of bursting keywords and the consensus set was calculated as its stability score. A higher value indicates greater consistency with the cross-set consensus.

Agreement with Global Strength Ranking (Spearman's ρ): The overall mean strength_index across all parameter sets was first calculated for each keyword to establish a "global strength" ranking. Each parameter set's internal keyword strength ranking was then compared to this global ranking using Spearman's correlation. The correlation coefficient was linearly mapped to [0, 1] to serve as the stability score.

Additionally, we performed a sensitivity analysis: a "baseline parameter set" (e.g., "G0.5_W3_L1") was specified. For any other parameter set, the Jaccard similarity (set level) of keyword sets and the Spearman correlation (order level) of keyword strength rankings with the baseline set were calculated. The average of these two values was used as the sensitivity indicator of that parameter set relative to the baseline. This table was exported separately in the results, allowing reviewers to quickly assess the consistency of burst sets and rankings under parameter perturbations.

6) Indicator Normalization, Weight Configuration, and Comprehensive Scoring

To enable comparable comprehensive ranking across multidimensional metrics, we normalized the scores of four key indicators for each parameter set: Explainability (mean coverage), Discriminability, Stability score, and Realism (median lift ratio at the keyword level). Optional Winsorization (default: clipping at the 1st and 99th percentiles) could be applied before normalization to mitigate the influence of extreme values. Normalization was performed by scaling to [0, 1]. Configurable weights were then introduced (default: equal weight for all four indicators). Defensive re-standardization was performed if all values were missing or zero. Finally, a comprehensive score (overall_score) was calculated using a "weighted average / effective item weighted average." The output included three types of tables for each parameter set: raw summaries, normalized values, and comprehensive scores, ensuring transparent comparison and reproducibility.

7) "Recent Bursts" and Temporal Recency Annotation

To highlight temporal recency, we annotated whether the start year of each burst interval fell within a window of the "most recent three years" (recent_years = 3) (Boolean variable is_recent_start). This annotation can be used to prioritize the display of recently emerging burst themes, distinguishing them from historical ones.

8) "Recent Bursts" and Temporal Recency Annotation

To highlight temporal recency, we annotated whether the start year of each burst interval fell within a window of the "most recent three years" (recent_years = 3) (Boolean variable is_recent_start). This annotation can be used to prioritize the display of recently emerging burst themes, distinguishing them from historical ones.

9) Result Export and Structured Outputs

The script wrote three core types of outputs to disk:

- Detailed Burst Table per Parameter Set: Contains parameter ID, keyword, burst level, strength_index, start/end slices and years, duration length, keyword coverage and realism ratios, and recent burst flag.
- Summary Table per Parameter Set: Contains the number of bursts, number of keywords involved, mean/median/max of level and strength, longest duration in years, number of recent new bursts, average coverage, median realism, and discriminability for each set.
- Comparative Master Table (with Scores): Integrates stability metrics (consensus Jaccard or Spearman-to-global), the four Winsorized and normalized scores, and their weighted comprehensive score. Two additional worksheets, "Consensus Keyword Set" and "Scoring Configuration Snapshot," were included to fix the scoring criteria and parameters for this experiment, ensuring traceability and reproducibility.

10) Visualization

To enhance the interpretability of the results, we provided two types of visualizations:

- Burst Heatmap: For a selected parameter set (e.g., baseline "G0.5_W3_L1"), the burst level of each keyword in each year (natural year mapped from slices) was plotted as tiles. A stratified color scheme highlighted levels ⩾ 2, with asterisks marking cells meeting the threshold. A density bar showing the annual count of bursting words was superimposed above the heatmap to depict yearly burst density horizontally. A bar chart on the right displayed the burst occurrence count for each keyword, serving as supplementary evidence for keyword ordering.
- Burst Timeline: For the top 36 representative keywords from the heatmap 排序 (sorting), a blue background line spanning 2015–2025 represented the "observable timeline." Red segments superimposed on this line indicated burst intervals (level ⩾ 2), with red dots marking the start years. If an interval extended beyond 2025, it was shown with a dashed line. Both graphics collectively present key information: "when it happened, how long it lasted, how strong it was, and if it was concentrated in specific years," corroborating the measures of strength_index, coverage, and realism in the tables.

11) Rationale for Method Selection and Comparison with Alternatives

The advantages of Kleinberg burst detection are: Firstly, its multi-scale detection based on a state machine inherently suppresses false positives caused by random noise. Secondly, the trade-off between "sensitivity-specificity" can be controlled via the grid of γ, ω, and minimum length L, complemented by stability and realism metrics to achieve a triple balance between "model-evidence-reality." Thirdly, the combined index strength_index (level × duration) intuitively captures "intensity × persistence," facilitating ranking and management. Furthermore, unlike simply identifying the "most frequent" keywords, burst detection is more sensitive to "growth rate/explosiveness," revealing recent, short-lived yet important thematic trends. Compared to methods relying solely on "trend regression," Kleinberg is particularly effective for non-stationary bursts. We use sensitivity analysis and stability metrics as guardrails to prevent conclusions based on chance due to a single parameter choice.

12) Reproducibility and Hyperparameter Recording

All key hyperparameters (γ, ω, L, s_base, recent_years, pre_window_slices, Winsorization upper/lower quantiles, the four weights, stability metric choice) were explicitly configured at the top of the script, and a "configuration snapshot" was exported with the results. Output paths were automatically derived based on the input file location, and all intermediate and final result files (CSV and Excel workbooks) were managed within the same directory. This design ensures that any researcher can reproduce identical burst results and score rankings given the same input and parameter conditions.
