# Supplementary material for: Thematic Mapping and Evolution of Social Media Mining in Health Research: Hybrid Bibliometric Synthesis
Source: J Med Internet Res. 2026 May 8;28:e86200. doi: 10.2196/86200 (PMC13160668; doi:10.2196/86200)
Supplement: Multimedia Appendix 3 [file jmir-v28-e86200-s003.pdf]

Multimedia Appendix 3. Mathematical derivation and computational process documentation for the hybrid semantic-structural bibliometric analysis pipeline

Note:

This document, based on the provided Python scripts, elaborates in detail the mathematical logic and computational procedures of a hybrid semantic–structural bibliometric analysis pipeline. The pipeline is applied to PubMed-indexed literature on SMM for thematic clustering and strategic mapping. Core steps include data cleaning, keyword co-occurrence network construction, structural strength calculation, semantic embedding and similarity computation, strength integration, UMAP–HDBSCAN clustering, as well as temporal dynamics analysis. Each step is accompanied by mathematical derivations, formula explanations, and implementation details to ensure reproducibility and transparency. The pipeline emphasizes the principles of open science, enabling an automated transition from static mapping to a predictive compass for research guidance.

Overview of the Pipeline

The goal of the pipeline is to identify thematic clusters within the SMM literature and to strategically position them along three dimensions: maturity, influence, and recency. The input data consist of PubMed article metadata (PMID, year, title, abstract, keywords, etc.). The mathematical foundation integrates graph theory (co-occurrence networks), embedding models (SPECTER2 and PubMedBERT), similarity measures (Jaccard, Cosine, Association Strength), and dimensionality reduction with clustering (UMAP + HDBSCAN).

The pipeline is structured into the following stages:

1) Data Preparation: cleaning and filtering.
2) Structural Analysis: co-occurrence–based strength computation.
3) Semantic Analysis: embedding-based similarity calculation.
4) Fusion: integration of structural and semantic strength matrices.
5) Clustering: thematic discovery.
6) Temporal Dynamics: sliced analysis over time.

Formally, let the keyword set be $K = \{k_1, k_2, \dots, k_n\}$, the article set be $A = \{a_1, a_2, \dots, a_n\}$, and the frequency f_i denotes the number of articles in which keyword k_i appears. $K_a \subseteq K$ means the subset of keywords assigned to article *a*. $A_i = \{a \in A | k_i \in K_a\}$ is the set of articles containing keyword k_i, *i* and *j* are indices for keywords, *a* is the index for article and *d* is index for vector dimensions.

1) Data preparation

1.1) Data reading and column mapping

The script (load_clean_01_final_XX.py) reads the Excel file and maps the column names (e.g., PMID, Year, Title, Abstract, Keywords). Mathematically, this can be formulated as a set mapping: for a candidate column name set $C = \{c_1, \dots, c_p\}$, we identify the we identify the corresponding matches in the dataframe columns *D* using normalized comparison (case- and whitespace-insensitive):

$find(D, C) = \arg \min_{d \in D} dist(d, c)$, where where dist is a string distance (e.g., Levenshtein).

This ensures robustness of the pipeline by preventing failures due to variations in column naming.

1.2) Data cleaning

For a title–abstract string *s*, the cleaning function is defined as:

$s' = lower(s)$, $s'' = replace\_punct\_to\_space(s')$, $s''' = collapse\_spaces(s'')$.

If digits are not preserved, then $s'''' = remove\_digits(s''')$.

Mathematically, this corresponds to a sequence of regular expression transformations, ensuring standardized input for subsequent embeddings.

1.3) Keyword Splitting and Deduplication

The keyword string *s* is split using a set of delimiters

$$S = \{;,,,|,\ldots\} :$$

$$split(s) = \bigcup_{sep \in S} str.split(s, sep) \ .$$

The resulting tokens are then uniquified: $uniq = \{t | t \in split(s), t \notin seen\}$, where the set *seen* prevents duplicates.

This produces a unique keyword list for each article, which is subsequently used for co-occurrence counting. The frequency of keyword k_i which means the number of articles in which it appears is computed from the article–keyword (AK) matrix:

$f_i = \sum_{a \in A} \mathbb{I}(k_i \in a)$, where $\mathbb{I}$ denotes the indicator function that equal to 1 if the condition is true and 0 otherwise.

1.4) Filtering: Frequency Threshold and Removal of Top-2 Terms

A whitelist is constructed as $K' = \{k_i | f_i \geq 2, k_i \notin Top2\}$, where *Top2* refers to the two most frequent keywords.

Generic terms (e.g., "social media") are removed in order to focus on more specific topics. The output consists of the filtered AK matrix and frequency table.

2) Structural analysis: Construction of the co-occurrence network and structural strength

2.1) Co-occurrence Counting (build_graphs_03_xx.py)

Using the whitelist K' , for each article we take its keyword subset $K_a \subseteq K'$ and count all keyword pairs:

$$c_{ij} = \sum_{a \in A} \mathbb{I}(k_i \in K_a \wedge k_j \in K_a) \ , i < j \ .$$

Here, c_{ij} denotes the co-occurrence frequency of keywords k_i and k_j which means the number of articles in which both appear. Only pairs with $c_{ij} \geq MIN\_COOCC(default = 1)$ are retained.

This procedure generates the co-occurrence counts for keyword pairs, forming the basis of the network edges.

2.2) Structural Strength Calculation (strength_struct_from_cooccurrence_05b_xx.py)

The structural strength matric S_{struct} is constructed by co-occurrence. Based on c_{ij} , f_i and f_j , three structural strength measures are computed:

Jaccard Similarity between keyword k_i and keyword k_j:

$$J_{ij} = \frac{c_{ij}}{f_i + f_j - c_{ij}} \ , \ if \ f_i + f_j - c_{ij} > 0; \quad else \ 0 \ .$$

Jaccard measures the ratio of intersection over union, making it suitable for sparse co-occurrence data. A higher Jaccard value indicates stronger association between keywords.

Cosine Similarity: $cos_{ij} = \frac{c_{ij}}{\sqrt{f_i \cdot f_j}}$ $, if\ f_i f_j > 0\ \ ;\quad else\ 0$.

Cosine normalizes co-occurrence by the geometric mean of individual frequencies, highlighting proportional co-occurrence strength.

Association Strength (AS): $AS_{ij} = \frac{c_{ij}}{f_i \cdot f_j}$ $, if\ f_i f_j > 0\ \ ;\quad else\ 0$.

Association Strength emphasizes the links of low-frequency keywords, giving more weight to rare but informative co-occurrences.

Output: Both a long-format table (*i, j, strength*) and a symmetric matrix (with zeros for missing pairs) are generated.

The structural strength matric S_{struct} is set by default to Association Strength (AS) as the structural metric during fusion. In our study and attached script, Jaccard and Cosine are also calculated for validation and as a fallback. Our study defaults to AS during the following fusion stage because AS assigns higher weights to low-frequency words, facilitating the discovery of rare but information-rich co-occurrence relationships. Since this paper provides the complete script, other researchers may freely choose to use any one of the three indicators as the structural strength metric.

Thus, $S_{struct} \equiv AS\ \in\ \mathbb{R}_{\geq 0}^{n_s \times n_s}$.

3) Semantic Analysis: embedding-based similarity calculation

3.1) Article Embeddings (embeddings_semantic_04_xxx.py)

Titles and abstracts are embedded using SPECTER2 and PubMedBERT:

$$e_a^{(\cdot)} = \begin{cases} e_a^{spec} = SPECTER\ (t_a + [SEP] + ab_a) \\ e_a^{bert} = PubMedBERT\ (t_a + [SEP] + ab_a) \end{cases}$$

With batch size = 8 and maximum sequence length = 512.

$e_a^{(\cdot)}$ is semantic embedding vector of article a based on either title or abstract,

t_a denotes title of article a,

ab_a denotes abstract of article a,

$[SEP]$ denotes special separator token used in transformer models.

Pre-trained models capture semantic meaning. SPECTER2 is optimized for scholarly document representation, while PubMedBERT specializes in biomedical texts.

3.2) Keyword Vector Aggregation

Keyword embeddings are obtained via mean aggregation:

$$v_i^{(\cdot)} = \frac{1}{|A_i|} \sum_{a \in A_i} e_a^{(\cdot)}\ \ ,\quad A_i = \{a | k_i \in K_a\}$$

Where $v_a^{(\cdot)}$ denotes aggregated embedding vector of keyword k_i, computed as the mean of embeddings of all articles containing keyword k_i.

The semantic representation of a keyword is assumed to be the average of embeddings of the articles in which it appears.

3.3) Normalization

We perform row-wise normalization on the vectors $v_a^{(\cdot)}$, using Z-score normalization by default for each dimension of every keyword vector:

$v'_{id} = \frac{v_{id}-\mu_i}{\sigma_i+\epsilon}$ $, row\_wise\ (per\ keyword)$,

where:

v_{id} denotes the the value of keyword k_i on dimension d,

μ_i denotes the mean of all dimensions of keyword k_i,

σ_i denotes the standard deviation of all dimensions of keyword k_i,

ϵ denotes a small constant (e.g. 10^{-6}) to avoid division by zero,

v'_{id} denotes the standardized result and v'_i denotes the standardized vectors.

Each keyword vector is normalized by subtracting its mean and dividing by its standard deviation, so that the distribution has mean 0 and variance 1.

3.4) Similarity Matrix

Cosine similarity between normalized keyword vectors v_i and v_j is computed as:

$$S_{spec}(i,j) = \frac{v_i'^{spec} \cdot v_j'^{spec}}{\|v_i'^{spec}\| \|v_j'^{spec}\|}$$

$$S_{bert}(i,j) = \frac{v_i'^{bert} \cdot v_j'^{bert}}{\|v_i'^{bert}\| \|v_j'^{bert}\|}$$

To integrate results, the mean of SPECTER2- and PubMedBERT-based similarities is used:

$$S_{sem}(i,j) = \frac{S_{spec}(i,j) + S_{bert}(i,j)}{2}$$

where:

$S_{spec}(i,j)$ denotes cosine similarity derived from SPECTER2 embeddings,

$S_{bert}(i,j)$ denotes cosine similarity derived from PubMedBERT embeddings,

$S_{sem}(i,j)$ denotes integrated similarity, computed as the average of SPECTER2- and PubMedBERT-based similarities.

Batched computation is employed to prevent memory overflow. Outputs are stored both as a long-format table and as a symmetric matrix.

4) Fusion: integration of structural and semantic strength matrices （strength_fusion_06_fixed-250818.py）

This study employs a strategy combining strict intersection and Min–Max normalization followed by linear weighting, using default parameter α=0.5 to eliminate nodes with all zeros and derive the hybrid matrix.

4.1) Alignment of nodes and edges (Intersection)

We first extract the set of keywords representing all terms appearing in the structural matrix S_{struct} : V_{struct} and the set of keywords representing all terms appearing in the semantic matrix S_{sem} : V_{sem}.

The set of nodes: $V^* = V_{struct} \cap V_{sem} = \{k_i, \dots\dots\}$, the nodes are essentially keywords that appear in both matrices.

The edges represent co-occurrence relationships between keywords, with this study representing the semantic association between two keywords. The edge set:

$E^* = E_{struct} \cap E_{sem} = \{(k_i, k_j), \dots\dots\}$, where E_{struct} is the set of all keyword pairs with co-occurrence frequency ⩾ 1 in structural matric and E_{sem} is the set of all keyword pairs with co-occurrence frequency ⩾ 1 in semantic matric.

4.2) Unify the dimensions of the two strength matrices

We normalize the values of both the "structural strength matrix" and the "semantic similarity matrix" to the range [0,1] to prevent any single view from dominating the fusion due to differing scales.

Both are Min-Max normalized to [0, 1]:

$$\tilde{S} = \begin{cases} \tilde{S}_{struct,ij} = \dfrac{S_{struct,ij} - \min(S_{struct})}{\max(S_{struct}) - \min(S_{struct})} \\ \tilde{S}_{sem,ij} = \dfrac{S_{sem,ij} - \min(S_{sem})}{\max(S_{sem}) - \min(S_{sem})} \end{cases}$$

Where min(·) denotes the minimum value of all non-empty elements in this matrix and max(·) denotes the maximum value of all non-empty elements in this matrix.

Next, Using linear weighting (default α=0.5) the merged strength "M" after fusion is obtained:

$$M_{ij} = \alpha\tilde{S}_{struct,ij} + (1-\alpha)\tilde{S}_{sem,ij}, \begin{cases} \boldsymbol{\alpha = 0.5, structure\ and\ semantics\ are\ equally\ weighted} \\ as\ \alpha\ increases: more\ emphasis\ is\ on\ structure\ co-occurrence \\ as\ \alpha\ decreases: more\ emphasis\ is\ on\ semantic\ cooccurrence. \end{cases}$$

Thus, M_{ij} represents the blending strength between keyword k_i and keyword k_j. After removing all zero nodes, all non-zero M_{ij} are fused together to obtain the symmetric matrix of the merged weights M.

4.3) Fusion

In graph theory, we merge distinct graphs into a single graph: $G_{fushion} = (V^*, E^*, M)$.

5) Clustering: UMAP + HDBSCAN

5.1) Prepare the distance matrix

To adapt metric learning and density-based clustering, the merged similarity matrix *M* is converted into a distance matrix *D*:

$$D_{ij} = 1 - M_{ij}$$

5.2) Dimension reduction by UMAP

UMAP minimizes the cross-entropy objective on the neighborhood graph defined by *D*, mapping high-dimensional structures to *Z*, and outputs results ranging from one to 20 dimensions along with clustering labels. Because the default dimension is 20 in our research and scripts.

5.3) Density-based clustering by HDBSCAN and hierarchical selection

We run Hierarchical Density-Based Spatial Clustering of Applications with Noise (HDBSCAN) based on *Z*.

First, we defined three Profile categories: robust, balanced, and fine. For each Profile, we sequentially set the corresponding minimum neighbor count min(sample): 20(Robust and low sensitivity imply greater resistance to noise and an emphasis on macro themes), 12(Moderate balance means achieving both stability and fine-grained control) and 8(Fine-grained, high-resolution means identifying small but semantically cohesive clusters). This value serves as a threshold parameter defining density. It means that for any

point/keyword, if there are at least min(sample) points within a certain radius, that point is considered a core point. A larger min(sample) value requires denser clusters. Conversely, a smaller value facilitates the formation of finer, more fragmented clusters. In Addition, this paper and the provided script set three automatically attempted target cluster numbers (cl): 4, 5, and 6. Therefore, nine cluster results will ultimately be generated. Authors and other researchers citing this work can select the final scientifically sound cluster results based on parameter calculation outcomes and the interpretability of the results. The results will be generated by Python as a table and visualizations for "Target vs Actual Cluster Count (target_cl_profile_got_cl)" for your selection.

First, compute the distance from keyword *k* to all other points of keywords, sort them in ascending order, and take the min(sample)th distance to obtain the core distance:

$$d_{core}(k) = distance\ from\ k\ to\ its\ \min(\text{sample})^{th}\ neareast\ keyword.$$

Then we calculate mutual reachability distance, which means that it takes into account not only the physical distance between two points, but also the density of the areas they are located in :

$$d_{mr}(k_i, k_j) = max\{d_{core}(k_i), d_{core}(k_j), d(k_i, k_j)\},$$

where $d(k_i, k_j)$ denotes the physical distance between the two points. This approach ensures that high-frequency keywords are not artificially grouped together due to their frequent occurrence. It also guarantees that low-frequency keywords with consistent semantic meaning are recognized as belonging to the same cluster.

Based on the mutual reachability distance, generate the minimum spanning tree (MST) which connects all data points with the minimal cumulative edge weight while preserving the underlying density structure to form condensed cluster tree. Then, based on cluster stability as the criterion, the clusters are pruned to obtain the final clusters, and the probability of each keyword appearing in each cluster is provided.

This process will yield the following results:

- Cluster label: $c_i \in \{1, \ldots, cl_{got}\} \cup \{-1\}$, where -1 is the noise cluster,
- Membership probability of each points/keywords: $p_i \in [0,1]$,

and the following metrics that evaluate clustering quality from different perspectives:

- Silhouette coefficient:
  This is an indicator measuring the compactness and separation of each sample within its respective cluster.
  For each sample/keyword k, a(k) is the average distance from sample k to other samples within the same cluster that means the intra-cluster compactness; b(k) is the average distance from sample k to the nearest neighboring cluster which is the inter-cluster separation.
  Calculate the contour coefficients: $s(k) = \frac{\text{b(k)} - a(\text{k})}{max\{\text{a(k)}, \text{b(k)}\}} \in [-1,1]$ and the overall Silhouette: $S = \frac{1}{n}\sum_{k=1}^{N} s(k)$, where k is the number of got clusters and N is the total number of samples.
  If S approaches 1, it indicates that the sample is very close to its own cluster and distant from other clusters, suggesting good clustering performance. If S approaches 0, it indicates that the sample is located near a boundary, representing an unclear separation. If S < 0, it indicates that the sample has been misclassified into the wrong cluster.
- Calinski–Harabasz(CH) index:
  Calculate the mean of the entire sample: $\bar{x} = \frac{1}{n}\sum_{k=1}^{N} x(k)$, where x(k) denotes 20-dimensional UMAP embedding vectors for each keyword;
  the sample set of clustered thematic clusters: C_c;
  the mean of the cth cluster: $\bar{x}_c = \frac{1}{|C_c|}\sum_{x(k) \in C_c} x(k)$;

the within-cluster dispersion: $W = \sum_{c=1}^{C} \sum_{x(k) \in C_c} \|x(k) - \bar{x}_c\|^2$;
the between-cluster dispersion: $B = \sum_{c=1}^{C} | C_c| \cdot \|x(k) - \bar{x}_c\|^2$;
CH index could be calculated: $CH = \frac{B/(C-1)}{W/(N-C)}$.
The higher the CH value, the better, indicating greater cohesion within each thematic cluster and stronger differentiation between themes.

- Davies-Bouldin (DB) index:
  The intra-cluster dispersion of each clusters which means the average distance of points to their cluster centroid: $S_c = \frac{1}{| C_c|} \sum_{x(k) \in C_c} \|x(k) - \bar{x}_c\|^2$;
  The inter-cluster separation between two clusters is defined as the distance between their centroids: $d_{ij} = \left\|\bar{x}_{C|i} - \bar{x}_{C|j}\right\|$;
  for each cluster compute its maximum similarity to any other cluster: $R_{C|i} = \max_{j \neq i} \left\{\frac{s_{C|i} - s_{C|j}}{d_{ij}}\right\}$;
  thus, the DB index is the mean of R_i: $DB = \sum_{C|i=1}^{C} R_{C|i}$.
  The closer the DB value is to 0, the better, indicating good inter-cluster separation and minimal overlap.
- Coverage:
  Coverage is the proportion of non-noise points: $Coverage = \frac{N_{non-noise}}{N_{total}}$, this value should not be too small.
- Stability:
  First convert the mutual reach distance into a density metric: $\lambda_{ij} = \frac{1}{d_{mr}(k_i, k_j)}$,
  when the algorithm begins at the densest points (λ → ∞), each point initially forms an independent small cluster. As λ decreases and the density threshold is relaxed, some small clusters begin to merge, and new clusters appear at certain λ values. As density continues to decrease and λ becomes smaller, a cluster is gradually absorbed into a larger one until it disappears. At this point, the cluster disappears. Therefore, each cluster C_c has a pair of definitions:

$$\begin{cases} \lambda_{appear}\big(x(k)\big) = \lambda \text{ at which } C_c \text{ first appears} \\ \lambda_{appear}\big(x(k)\big) = \lambda \text{ at which } C_c \text{ merges into another cluster} \end{cases}$$

  the stability index is:

$$Stability(C_c) = \sum_{x(k) \in C_c} (\lambda_{appear}\big(x(k)\big) - \lambda_{disappear}(x(k))$$

  The higher this stability value, the better, indicating greater cluster stability and a wider range of hierarchical levels, which signifies its reality and reliability.

One can pick the most reasonable clustering results based on these parameter calculations and graphical outputs for their own research.

6) Temporal dynamics: sliced analysis over time

This study divided the decade into three time periods based on the annual publication volume of papers and performed the clustering analysis described in the previous step for each period.

We attempt to evaluate the continuity of topics with edge jaccard overlap test:

$Overlap(t, t+1) = \frac{|E_t \cap E_{t+1}|}{|E_t \cup E_{t+1}|}$, where E_t denotes the set of co-occurrence edges for keywords in time slice t. This measure measures the continuity of topic structure across adjacent time slices. A higher value indicates a more stable and continuous core topic relationship. Furthermore, this article incorporates the timeline of burst keywords to help explain these changes.
