# Supplementary material for: Thematic Mapping and Evolution of Social Media Mining in Health Research: Hybrid Bibliometric Synthesis
Source: J Med Internet Res. 2026 May 8;28:e86200. doi: 10.2196/86200 (PMC13160668; doi:10.2196/86200)
Supplement: Multimedia Appendix 4 [file jmir-v28-e86200-s004.pdf]

Multimedia Appendix 4. Micro-level interpretive triangulation with selected articles (evidence mapping)

No.	Quadrant	Keyword	Cluster	Metric	PMID	IF	H_index	RCR	Country	Plattform	Keywords	Info	Title	Journal	Year	Article description	Contributions of the study	Limitations	Future research directions
**1**	Peripheral Mature Theme (X⩾ 0, Y<3.51, Z<0.5)	depression	Cluster 1(Noise)	IF_max，Hindex_max，RCR_max	29456286	8.9	275	4.950371722	USA	Reddit	Anxiety Disorders; Consumer Health Information; Depression; Post-Traumatic; Stress Disorders; Unsupervised Machine Learning.	Park et. al.(2018) [1]	Examining Thematic Similarity, Difference, and Membership in Three Online Mental Health Communities from Reddit: A Text Mining and Visualization Approach.	Computers in human behavior	2018	From three Reddit communities, we collected online discussions related to three mental health conditions—anxiety, depression, and post-traumatic stress disorder (PTSD). First, we applied the Unsupervised Machine Learning - k-means algorithm to cluster and identify the most frequently discussed topics. Then, through qualitative analysis, we explored these topics in greater depth. Finally, by using visualization techniques, we gained a deeper understanding of the relationships among these topics.	This research provides a more fine-grained examination of how online communities with similar symptoms and comorbidities differ in the nature of their discussions. The subtle differences revealed through these analyses can inform the design of online mental health communities and patient education programs for these conditions.	Data from social networking platforms reflect the biases of their user populations and cannot fully represent the needs of all individuals with these conditions.<br>Online discussion topics often evolve over time, and many lengthy threads involve multiple themes, which cannot be adequately captured by methods that only identify a single theme.<br>Social media discussions frequently contain spelling errors, abbreviations, omissions, and community-specific jargon, which are difficult to accurately recognize.<br>For better visualization effects, not all words were included in the analysis.	Employ machine learning algorithms capable of generating multiple topics (e.g., Latent Dirichlet Allocation, LDA).<br>Explore how to design online health communities that can simultaneously address two distinct needs: immediate problem-solving or emotional venting, and long-term solution seeking.<br>Investigate the differences between informational and emotional support provided in social media discussions and those delivered by internet-based interventions, as this has important research value.<br>They recommend that researchers adopt multiple methodological approaches to comprehensively understand the subtle differences in similar discussions within online health communities.
**2**	Peripheral Mature Theme (X⩾ 0, Y<3.51, Z<0.5)	communicable diseases	Cluster 4	RCR_max	32293582	3.9	64	6.39386164	USA	Weibo	COVID-19; coronavirus; infectious disease; infodemiology; infoveillance; social media, surveillance	Li et. al.(2020) [2]	Data Mining and Content Analysis of the Chinese Social Media Platform Weibo During the Early COVID-19 Outbreak: Retrospective Observational Infoveillance Study.	JMIR public health and surveillance	2020	This study aims to utilize social media-based "information monitoring" methods to explore the public's cognition, attitudes, and behaviors during the pandemic. The paper collected posts from the Chinese social media platform Weibo originating from Wuhan between December 23, 2019, and January 30, 2020. The research team then employed a quantitative linear regression model to predict the relationship between COVID-19-related posts and the number of confirmed cases. Subsequently, qualitative content analysis and inductive manual coding were applied to identify parent categories of news and user-generated themes related to COVID-19. Finally, the team integrated related themes, removed duplicates, and assessed thematic consistency by independently coding a sample of posts.	Based on a quantitative and qualitative analysis of Chinese social media data from the early stage of the outbreak in Wuhan, this study provides preliminary insights into the origins of the COVID-19 pandemic.	Since the data were drawn from a specific time period and a single social media platform, the findings may not be generalizable to a broader or more comprehensive discussion of the issue.<br>During the study period, as there was no official name for the disease yet, social media users may have used other keywords to refer to COVID-19, resulting in those data not being captured.<br>The use of a simple linear regression to examine the relationship between social media discussion volume and case numbers without controlling for other potential confounding factors limits the transferability of the conclusions.	Future studies should continue to explore the value of social media data in predicting COVID-19 severity, measuring public responses and behaviors, and evaluating the effectiveness of pandemic communication.<br>Future research should expand data collection to include conversations from multiple platforms and incorporate natural language processing and machine learning methods to facilitate the classification of larger-scale dialogues.
**3**	Peripheral Mature Theme (X⩾ 0, Y<3.51, Z<0.5)	communicable diseases	Cluster 4	IF_max，Hindex_max	33882015	6	214	0.631341386	USA	Twitter	COVID-19; COVID-19 intervention; Twitter; coronavirus; human behavior; infectious disease; infodemiology; infoveillance; mobility; shelter-in-place; social distancing; social media	Daughton et. al.(2021) [3]	Mining and Validating Social Media Data for COVID-19-Related Human Behaviors Between January and July 2020: Infodemiology Study.	Journal of medical Internet research	2021	This study aims to monitor public cognition in real-time through discussions on social media, thereby informing public health communication. By analyzing conversations on Twitter, it seeks to identify behaviors related to the spread of COVID-19 and the impact of the pandemic on individual perceptions.<br><br>The research team collected 7,278 English tweets posted between January and May 2020. The authors developed a coding scheme consisting of 6 categories and 11 subcategories, along with a supervised learning classifier. This classifier was used to assess temporal and spatial trends, identifying region-specific patterns and temporal variations	The methodology proposed in this study can more effectively capture the dynamic evolution of information on social media.	Social media data involve multiple biases, which is a known limitation of using internet-based data.<br>The data and results cannot reveal the causes of observed differences or the influencing factors behind them.<br>ystematic disparities exist in social media data.<br>Known biases arise from classification inaccuracies and classifiers tailored for specific research purposes.	In practical applications, it is essential to integrate internet data with traditional data streams to present a comprehensive and evolving picture. Future research should focus on targeted studies to better understand underlying biases.

																synchronized with real-world events. Finally, the signal strength was evaluated by comparing the results with other valuable data sources used to measure human behavior during the COVID-19 pandemic.			
**4**	Peripheral Obsolete Theme (X < 0, Y < 3.51, Z < 0.5)	rare disease	Cluster 2	RCR_max	26442199[4]			2.345324623	Denmark		clinical diagnosis decision support systems; data mining; information retrieval; machine learning; rare diseases; search engines	Svenstrup et. al.(2015) [4]	Rare disease diagnosis: A review of web search, social media and large-scale data-mining approaches.	Rare diseases (Austin, Tex.)	2015	This review centers on "rare disease diagnosis," integrating specialized search tools, Watson-style data mining, and social media/collective intelligence within a unified framework. The article focuses on three technological pathways for rare disease diagnosis: web and literature search, social media, and large-scale data mining. It begins by exploring the causes of errors in rare disease diagnosis, then evaluates the retrieval accuracy of Google (google.com), PubMed (pubmed.gov), OMIM (omim.org), and the research team's self-developed search tool, findzebra.com. Subsequently, the study introduces the Watson framework and discusses the potential and limitations of social media. Special emphasis is placed on the processing of unstructured data.<br><br>The study does not provide quantitative empirical analysis of "social media mining" but rather highlights directions and opportunities. First, the paper positions "social media" as one of the three pathways for rare disease diagnosis. The authors' approach is closer to a review of "platforms and mechanisms"—for example, mentioning physician-oriented social platforms and commercial attempts at "collective intelligence diagnosis"—but does not report new findings from specific "social media data mining experiments." They explicitly state that "quantification remains challenging at this stage."<br><br>The study provides a motivational evidence chain (e.g., diagnostic delays, the potential of collective intelligence) and application scenarios for the use of social media mining in medical diagnosis. It also introduces evaluation metrics (e.g., recall@10/20) that could be transferred to social media data mining assessment. However, its main contributions lie in the comparison of search engines and Watson, while the discussion on "direct mining of social media text" remains prospective rather than empirically substantiated.	The study systematically compares search and data mining methods, highlighting the value of specialized search tools. It presents Watson as a case study, bridging traditional search and intelligent question-answering systems. Finally, it outlines the potential of social media in diagnostic applications.	The effectiveness of social media is difficult to quantify, and there is a lack of systematic evidence. The experimental data are limited, with only 56 rare disease cases included.	Technologically, developing more powerful and robust machine learning and natural language processing systems.<br>Methodologically, creating integrated systems that combine free-text and structured knowledge in a systematic manner.<br>Practically, leveraging the potential of social media by quantitatively evaluating the real-world impact of social media-based diagnostic platforms and addressing risks associated with non-expert user engagement.
**5**	Peripheral Obsolete Theme (X < 0, Y < 3.51, Z < 0.5)	medium(Media)	Cluster 1(Noise)	IF_max	26980151	4.7	147	1.362643079	USA	Twitter	Media; Social marketing; Socioeconomic status	Dai & Hao (2017) [5]	Mining social media data for opinion polarities about electronic cigarettes.	Tobacco control	2017	This study collected 757,167 tweets about e-cigarettes from Twitter between July 23 and October 14, 2015, to assess public attitudes toward e-cigarettes. Using a Naïve Bayes model, the collected tweets were classified. The	Research on opinions about e-cigarettes from social media can further support educational campaigns and help evaluate their effectiveness.	The sample was drawn solely from Twitter, whose user base exhibits distinct biases. The study also did not explore the impact of local e-cigarette regulations.	Future studies should assess how regulations influence public attitudes toward e-cigarette use.

																analysis further examined the prevalence of e-cigarette-related tweets, geographic variations, and the influence of socioeconomic factors on public perceptions.			
**6**	Peripheral Obsolete Theme (X < 0, Y < 3.51, Z < 0.5)	electronic nicotine delivery systems	Cluster 4	RCR_max	27956376	3.9	64	2.843636994	USA	Twitter	Internet; e-cigarettes; social media; tweet	Lazard et. al.(2016) [6]	E-Cigarette Social Media Messages: A Text Mining Analysis of Marketing and Consumer Conversations on Twitter.	JMIR public health and surveillance	2016	This study analyzes the attitudes of commercial and consumer users toward e-cigarettes by examining discussions on Twitter. A total of 872,544 tweets and retweets were collected. Topics within these tweets were categorized and quantified using Expectation Maximization clustering in SAS Text Miner. The results were interpreted and validated through the Topic Viewer tool and manual analysis.	This study is the first to mine trending themes and topics related to e-cigarettes from large-scale public discussions on social media. Furthermore, it reveals proactive consumer dialogues. Beyond brand and distributor promotions, the study identifies substantial consumer-initiated conversations on Twitter, including policy debates, personal usage experiences, and distinctions between e-cigarettes and traditional tobacco. Finally, the research holds significant implications for public health, suggesting that public health advocates should actively engage in social media discourse to counterbalance commercial messaging.	The sample may not be fully representative, and the findings may not generalize to all Twitter users. The study can only reveal the content and themes shared by users and cannot assess how this content influences audience perceptions and attitudes. Inadequate noise reduction in the data represents another limitation, as the study relied on manual methods to filter irrelevant information rather than applying automated denoising techniques prior to analysis.	Methodological improvements: Future studies should incorporate automated noise reduction techniques to enhance the efficiency and accuracy of data analysis.<br>Impact evaluation: Further research should explore how these social media conversations influence public attitudes and usage behaviors.
**6**	Immature but Declining Theme (X<0, Y⩾3.51, Z<0.5)	tweet	Cluster 4	RCR_max	27956376	3.9	64	2.843636994	USA	Twitter	Internet; e-cigarettes; social media; tweet	Lazard et. al.(2016) [6]	E-Cigarette Social Media Messages: A Text Mining Analysis of Marketing and Consumer Conversations on Twitter.	JMIR public health and surveillance	2016	This study analyzes the attitudes of commercial and consumer users toward e-cigarettes by examining discussions on Twitter. A total of 872,544 tweets and retweets were collected. Topics within these tweets were categorized and quantified using Expectation Maximization clustering in SAS Text Miner. The results were interpreted and validated through the Topic Viewer tool and manual analysis.	This study is the first to mine trending themes and topics related to e-cigarettes from large-scale public discussions on social media. Furthermore, it reveals proactive consumer dialogues. Beyond brand and distributor promotions, the study identifies substantial consumer-initiated conversations on Twitter, including policy debates, personal usage experiences, and distinctions between e-cigarettes and traditional tobacco. Finally, the research holds significant implications for public health, suggesting that public health advocates should actively engage in social media discourse to counterbalance commercial messaging.	The sample may not be fully representative, and the findings may not generalize to all Twitter users. The study can only reveal the content and themes shared by users and cannot assess how this content influences audience perceptions and attitudes. Inadequate noise reduction in the data represents another limitation, as the study relied on manual methods to filter irrelevant information rather than applying automated denoising techniques prior to analysis.	Methodological improvements: Future studies should incorporate automated noise reduction techniques to enhance the efficiency and accuracy of data analysis.<br>Impact evaluation: Further research should explore how these social media conversations influence public attitudes and usage behaviors.
**7**	Peripheral Obsolete Theme (X < 0, Y < 3.51, Z < 0.5)	medium	Cluster 1(Noise)	RCR_max，Hindex_max	28341768	4.7	147	2.649292649	USA	Twitter	Electronic nicotine delivery devices; Media; Public opinion	Lazard et. al.(2017) [7]	Public reactions to e-cigarette regulations on Twitter: a text mining analysis.	Tobacco control	2017	In May 2016, the FDA included e-cigarettes under tobacco product regulations. This study analyzes discussions on Twitter to extract major topics from tweets, with the aim of understanding the public's initial reaction to this policy change and exploring the role of social media in shaping public attitudes. Using the NUVI software, Twitter data from May 1 to 17, 2016, were collected and analyzed through text mining methods to identify central themes in public discourse.	This research is the first to reveal the immediate social media response to the FDA's regulation of e-cigarettes. It finds that public reactions were largely negative or mixed, providing empirical evidence for understanding the communication effectiveness of policy rollout.	The short time frame and exclusive reliance on Twitter data limit the generalizability of the findings.	Examine long-term public opinion dynamics.<br>Compare responses across different social media platforms.
**8**	Peripheral Obsolete Theme (X < 0, Y < 3.51, Z < 0.5)	rare disease	Cluster 2	IF_max，Hindex_max	30200209	2.4	229	0.910399367	Spain	Facebook	Facebook; data mining; rare diseases; social media	Subirats et. al.(2018) [8]	Mining Facebook Data of People with Rare Diseases: A Content-Based and Temporal Analysis.	International journal of environmental research and public health	2018	This study explores how to handle information related to rare diseases by analyzing data from Spain on Facebook. The aim of this research is to help users interested in rare diseases maximize the	This study characterized rare disease data on social media, systematically analyzed Facebook interaction data, and revealed the dissemination characteristics of rare disease topics	Only Spanish posts were analyzed, and the results are not universally applicable across languages.<br>The data does not cover other countries or organizations.<br>The method is insufficient, as no sentiment analysis	Expand the data scope to include patients from other languages and countries.<br>Analyze more Facebook groups to increase sample diversity.<br>Improve the methodology by

																engagement of their posts and assist rare disease organizations in adjusting their priorities based on interests expressed on social media.<br>The research team first used Netvizz to download data from 3,917 Facebook posts. Text mining and text comparison were then conducted in R. Emotion analysis was performed using the TextBlob library in Python. Finally, time analysis was employed to explore temporal differences.	on social media from the perspectives of content and time. The results of the study provide practical recommendations for improving interaction rates.	was performed on the image content.	introducing image sentiment analysis to combine textual and visual information and enhance the depth of analysis.<br>Introduce machine learning applications, using supervised learning to predict interaction rates and unsupervised learning to characterize user profiles and behavioral patterns.
**9**	Peripheral Obsolete Theme (X < 0, Y < 3.51, Z < 0.5)	electronic nicotine delivery systems	Cluster 4	IF_max，Hindex_max	33052127	6	214	0.394533169	Macao, China	Zhihu	China; e-cigarettes; infodemiology; infoveillance; public opinion; regulation; social media	Wang et. al.(2020) [9]	Public Opinion About E-Cigarettes on Chinese Social Media: A Combined Study of Text Mining Analysis and Correspondence Analysis.	Journal of medical Internet research	2020	This study aims to track changes in public perception of e-cigarettes through social media discussions and to evaluate the impact of policy implementation on public attitudes and thematic focus, thereby providing insights for public health communication and policy enforcement. The research team collected 1,160 question-and-answer posts related to e-cigarettes from the Chinese Q&A platform Zhihu between January 2017 and December 2019. Text mining and correspondence analysis were employed to examine the evolution of public concerns by comparing high-frequency words and thematic distributions across different periods.	This is the first study to integrate text mining and correspondence analysis in systematically examining public discussions about e-cigarettes on Chinese social media, establishing a novel methodological framework for investigating the interaction between policy impact and public opinion.<br>Based on 1,160 Zhihu posts, it offers a longitudinal comparison of public discussions before and after two major e-cigarette policy interventions between 2017 and 2019, revealing how policy introduction influenced shifts in public discourse.<br>It demonstrates that social media can serve as a valuable source for understanding public concerns and tracking the effects of policy changes.	Discussions on Zhihu are initiated only by individuals willing to express their opinions online; thus, the views represented may not fully reflect broader public sentiment.<br>The study focused on extracting and quantifying high-frequency words and did not measure emotions, attitudes, or subjective viewpoints	To fully understand public reactions to e-cigarette regulation, future studies should explore the emotional and attitudinal dimensions within public discourse, which remains a significant and meaningful direction.
**10**	Peripheral Emerging Theme (X⩾0, Y<3.51, Z⩾0.5)	suicide	Cluster 1(Noise)	IF_max，Hindex_max，RCR_max	28694239	6	214	4.765509094	Hong Kong, China	Weibo	Chinese; machine learning; natural language; psychological stress; social media; suicide	Cheng et. al.(2017) [10]	Assessing Suicide Risk and Emotional Distress in Chinese Social Media: A Text Mining and Machine Learning Study.	Journal of medical Internet research	2017	This study applied computational linguistic analysis methods to assess suicide risk and emotional distress among Chinese social media users. A total of 974 Weibo users consented to participate, and their posts were collected. These data were then parsed and categorized into Simplified Chinese–Linguistic Inquiry and Word Count (SC-LIWC) categories. Subsequently, logistic regression was used to examine the associations between SC-LIWC features and five suicide risk factors. Finally, a Support Vector Machine (SVM) model was trained on the linguistic features to automatically classify whether Weibo users exhibited any of the five risk factors.	The study demonstrates that SC-LIWC can be employed to analyze linguistic markers of suicide risk and emotional distress in non-English social media, identifying features that differ from those previously reported in English-language research. It further shows that natural language on social media can serve as a marker to distinguish high-risk individuals from the general population, and that these linguistic markers are culturally sensitive. Some findings provide new hypotheses for future validation.	The machine classifiers developed in this study require further optimization, especially the classifiers for depression and stress.<br>The data-driven approach adopted in this study led to relatively loose results, with some findings being difficult to interpret.	Machine classifiers based on SC-LIWC features need further replication studies to validate the transferability of these findings.<br>Further optimization is required for SC-LIWC–based classifiers to be applicable in real-world scenarios.<br>Longitudinal studies should be conducted.<br>Machine-generated results should be verified, reviewed, and followed up by professionals.<br>Expert feedback and follow-up outcomes should be fed back to model developers to improve the algorithms..
**10**	Peripheral Emerging Theme (X⩾0, Y<3.51, Z⩾0.5)	psychological	Cluster 2	IF_max，Hindex_max，RCR_max	28694239	6	214	4.765509094	Hong Kong, China	Weibo	Chinese; machine learning; natural language; psychological stress; social media; suicide	Cheng et. al.(2017) [10]	Assessing Suicide Risk and Emotional Distress in Chinese Social Media: A Text Mining and Machine Learning Study.	Journal of medical Internet research	2017	This study applied computational linguistic analysis methods to assess suicide risk and emotional distress among Chinese social media users. A total of 974 Weibo users consented to participate, and their posts were collected. These data were then parsed and categorized into Simplified Chinese–Linguistic Inquiry and Word Count (SC-LIWC) categories. Subsequently, logistic regression was used to examine the associations between SC-LIWC features and five suicide risk factors. Finally, a Support Vector Machine (SVM)	The study demonstrates that SC-LIWC can be employed to analyze linguistic markers of suicide risk and emotional distress in non-English social media, identifying features that differ from those previously reported in English-language research. It further shows that natural language on social media can serve as a marker to distinguish high-risk individuals from the general population, and that these linguistic markers are culturally sensitive. Some findings provide new	The machine classifiers developed in this study require further optimization, especially the classifiers for depression and stress.<br>The data-driven approach adopted in this study led to relatively loose results, with some findings being difficult to interpret.	Machine classifiers based on SC-LIWC features need further replication studies to validate the transferability of these findings.<br>Further optimization is required for SC-LIWC–based classifiers to be applicable in real-world scenarios.<br>Longitudinal studies should be conducted.<br>Machine-generated results should be verified, reviewed, and followed up by professionals.<br>Expert feedback and follow-up outcomes should be fed back to model developers to improve the algorithms..

																model was trained on the linguistic features to automatically classify whether Weibo users exhibited any of the five risk factors.	hypotheses for future validation.		
**11**	Peripheral Emerging Theme (X⩾ 0, Y<3.51, Z ⩾0.5)	digital health	Cluster 1(Noise)	IF_max, Hindex_max, RCR_max	37083752	6	214	2.213968291	Hong Kong, China	Facebook, Twitter, Reddit, and 16 health forums	TCIM; cancer; cancer care; complementary; forums, digital health; integrative; machine learning; perceptions; social media; traditional; traditional, complementary, and integrative medicine	Lam et. al.(2023) [11]	The Use of Traditional, Complementary, and Integrative Medicine in Cancer: Data-Mining Study of 1 Million Web-Based Posts From Health Forums and Social Media Platforms.	Journal of medical Internet research	2023	This study investigated cancer patients' discussions of traditional, complementary, and integrative medicine (TCIM) across Facebook, Twitter, Reddit, and 16 health forums, using a mixed approach of manual evaluation and natural language processing (NLP) to explore patient usage, interest, and perceptions of TCIM in cancer care.<br><br>A total of 1,620,755 publicly available posts were analyzed. The research team first collected forum and social media data using Python web-scraping up to February 7, 2022. The data were then processed using NLP and categorized into three main types of TCIM to establish a database. Descriptive analysis was conducted to examine the most frequently discussed TCIM modalities for different cancers and symptoms. Sentiment analysis was performed with TextBlob to generate polarity scores, and researchers summarized positive and negative themes. Exploratory analysis employed Latent Dirichlet Allocation (LDA, implemented in Gensim), combining unsupervised and semi-supervised methods to reveal thematic patterns of patient discussions about specific TCIM modalities. Finally, keyword weighting and word clouds were used for visualization and manual review.	This study represents the first large-scale collection of TCIM-related cancer discussions across multiple health forums and social media platforms, analyzed with a combination of machine learning and NLP techniques. The article provides multidimensional insights: not only mapping TCIM usage patterns across cancer types and common symptoms, but also capturing guideline-endorsed therapies and emerging yet under-researched modalities. Drawing from patient-generated data, it delivers a more comprehensive perspective. Ultimately, the findings shed light on patient and caregiver interests, common practices, facilitators, and barriers regarding TCIM, offering evidence for clinical communication and underscoring the need for physicians to engage more openly in TCIM discussions.	Limited representativeness, as the data primarily came from online cancer communities, potentially underrepresenting patients who do not use forums or social media (especially older populations).<br>Language limitation, as only English-language posts were included, overlooking cross-cultural differences in TCIM use.<br>Methodological constraints: sentiment analysis is prone to misclassification (e.g., sarcasm detection, polarity bias across topics), although manual correction was applied to reduce errors.<br>The findings reflect popularity and interest, which cannot be directly equated with clinical effectiveness.	Future Directions:<br>Future studies should include posts in more languages and from diverse countries to examine cultural variations in TCIM usage and perceptions.<br>Comparative analyses across different online platforms could help identify differences in usage patterns and concerns.<br>Methodological refinement is needed to improve the ability of sentiment analysis and NLP models to capture complex semantics and reduce misclassification.<br>Extend this methodological framework to other chronic diseases beyond cancer.<br>Patient-generated data highlighting high-interest therapies could inform future clinical research in integrative oncology and other medical domains.
**12**	Core Emerging Hotspot (X⩾ 0, Y⩾3.51, Z ⩾0.5)	natural language processing	Cluster 2	RCR_max	25755127	4.6	184	9.376485361	USA	Twitter & DailyStrength	ADR; adverse drug reaction; deep learning word embeddings; machine learning; natural language processing; pharmacovigilance; social media mining	Nikfarjam et. al.(2015) [12]	Pharmacovigilance from social media: mining adverse drug reaction mentions using sequence labeling with word embedding cluster features.	Journal of the American Medical Informatics Association	2015	This article aims to explore how adverse drug reactions (ADRs) can be identified from informal social media texts to support public health surveillance and pharmacovigilance. The authors propose a machine learning–based concept extraction system, ADRMine, which employs Conditional Random Fields (CRFs) as its core framework. The model integrates multiple features, with particular emphasis on unsupervised clustering of word embeddings generated by deep learning to capture semantic similarity. Finally, the performance of ADRMine in the ADR extraction task was evaluated, confirming the effectiveness of word-cluster features.	The method enables efficient extraction of complex medical concepts from user-generated content without requiring large amounts of manual annotation. It demonstrates good scalability and practical applicability, offering a novel approach for social media mining and ADR surveillance.	The current approach still relies on task-specific feature engineering (e.g., contextual features, word-embedding clusters), which imposes some manual design constraints.<br>The study focused only on ADR extraction, without addressing the issue of normalization—that is, mapping extracted terms to standard medical ontologies.<br>The model's generalizability across different corpora remains limited and requires improvement.	Incorporate deep learning models by replacing CRFs with neural networks (NNs), enabling end-to-end learning of both features and labels, thereby reducing reliance on handcrafted features.<br>Advance research on normalization: future work should not only extract ADRs but also map them to standardized medical vocabularies to enhance their utility for pharmacovigilance.<br>Extend the proposed features and methods to other medical concepts (e.g., symptoms, diseases, treatments) for broader social media information extraction tasks.<br>Improve cross-corpus performance by further optimizing the approach to enhance generalizability across platforms and data types.
**13**	Core Emerging Hotspot (X⩾ 0, Y⩾3.51, Z ⩾0.5)	natural language processing	Cluster 2	Hindex_max	30721764	11.2	350	0.776323034	USA	Facebook, Twitter, and Reddit	Informatics; NLP; allergen immunotherapy; aseptic; compounding; infection; influenza; posts; preparation; safety; shot; vaccination	Blumenthal et. al.(2019) [13]	Mining social media data to assess the risk of skin and soft tissue infections from allergen immunotherapy.	The Journal of allergy and clinical immunology	2019	This study aimed to explore the potential risk of skin and soft tissue infections (SSTIs) associated with allergen immunotherapy (AIT) using social media data, and to compare these risks with those related to influenza vaccination. The authors collected and analyzed 428,832 text	This study contributes by demonstrating, through social media big data and NLP methods, that the risk of infection associated with AIT is extremely low, thereby supplementing existing clinical evidence.	Limited representativeness: adverse events on social media may be underestimated due to the exclusion of private posts, and the sample population is biased toward younger, internet-savvy users.<br>Methodological constraints: NLP may have missed relevant	Methodological optimization: improving NLP algorithms and search strategies to enhance recall and precision, supplemented with manual verification when feasible.<br>Expanding data sources: including a wider range of platforms and more diverse populations to

																posts published between 2012 and 2016 across more than 10 U.S.-based social media platforms, including Facebook, Twitter, and Reddit. Natural language processing (NLP) was first used to identify posts relevant to AIT or influenza vaccination, followed by manual review to screen for potential SSTI cases. Finally, the frequency of infection-related risks associated with the two interventions was compared.		posts, preventing accurate recall measurement.<br>Lack of clinical information to validate reported cases.	increase representativeness.<br>Cross-disciplinary integration: combining electronic health records (EHRs), pharmacovigilance databases, and social media data to develop a more comprehensive adverse event surveillance framework.<br>Future research should focus more on predicting clinical events during treatment, to support prevention and management.
**14**	Core Emerging Hotspot (X⩾ 0, Y⩾3.51, Z ⩾0.5)	covid-19	Cluster 4	RCR_max	32234709	3.9	64	11.12793947	Iran	Google Trends website	COVID-19; Google Trends; LSTM; coronavirus; incidence; linear regression; outbreak; pandemic; prediction; public health	Ayyoubzadeh et. al.(2020) [14]	Predicting COVID-19 Incidence Through Analysis of Google Trends Data in Iran: Data Mining and Deep Learning Pilot Study.	JMIR public health and surveillance	2020	This study focuses on using search engine data to predict the incidence trend of COVID-19 in Iran. The research team obtained relevant search data from the Google Trends platform and used two methods, linear regression models and long short-term memory neural networks (LSTM), to predict the number of confirmed cases. All models were evaluated using 10-fold cross-validation, and the prediction performance was measured by the root mean square error (RMSE).	This is the first study to use Google Trends data to predict COVID-19 incidence trends in Iran, providing data-driven evidence for early warning of the epidemic. A multi-model comparison was conducted, combining linear regression and LSTM deep learning models to explore the advantages and limitations of different modeling methods, demonstrating the potential of Google search data in epidemic prediction. The results provide a reference for public health communication and resource planning.	Google Trends only provides "interest scores" rather than absolute search volumes, limiting model accuracy.<br>Keywords are not comprehensive, potentially omitting other valuable search terms.<br>Insufficient data and limited training data led to overfitting of LSTM and large fluctuations in cross-validation results.<br>Insufficient representativeness: The model is based only on Iranian data, and the prediction results may not be directly applicable to other countries or regions.	Integrate data and combine more data sources to improve the comprehensiveness and accuracy of predictions.<br>Optimize keywords to systematically identify and update the most relevant search terms, thereby improving the efficiency of Google Trends data utilization.<br>Improve the model by increasing the training data volume and further optimizing deep learning models (e.g., LSTM) to reduce overfitting and enhance generalization capabilities.<br>Extend the research methodology to other countries or even a global scale.<br>Explore how to integrate the prediction model into public health decision-making processes to assist policymakers in efficiently allocating medical resources.
**15**	Core Emerging Hotspot (X⩾ 0, Y⩾3.51, Z ⩾0.5)	twitter	Cluster 4	IF_max	33983129	12.5	36	0.605619262	USA	Twitter	Twitter; cyberincivility; digital professionalism; health professions students; social media; social networking sites	De Gagne et. al.(2021) [15]	Analysis of Cyberincivility in Posts by Health Professions Students: Descriptive Twitter Data Mining Study.	JMIR medical education	2021	This study focuses on the behavior of health-related professional students on social media, particularly cyberincivility on Twitter. The purpose of this study is to describe the characteristics and examples of uncivil behavior posted by Twitter users who self-identify as health professionals, and to analyze the proportion and pattern differences of tweets deemed inappropriate or potentially offensive. The research method employs cross-sectional descriptive data mining, collecting tweet data from August 2019 to February 2020, with the sample including students from medical, nursing, dental, pharmacy, physician assistant, and physical therapy programs. Data analysis combined SAS 9.4 (for descriptive and inferential statistics, including logistic regression) and NVivo 12 (for text pattern analysis) to comprehensively present the manifestation of online uncivil behavior among health professional students.	This study contributes by revealing the phenomenon of online incivility among health professional students on Twitter and emphasizing the importance of digital professional literacy education.	Sample and data limitations: The study was designed as a retrospective observational study, analyzing only the most recent 20 tweets from each account, with some potential samples excluded. Additionally, student identities were not verified.<br>Insufficient sample representativeness: The sample primarily consists of nursing and medical students, with insufficient representation from other disciplines, resulting in potential bias.<br>Data collection was completed by February 2020, failing to account for the impact of subsequent major social events, such as the global spread of the COVID-19 pandemic.<br>Researcher bias: Cyberincivility is a highly emotional social issue, and interpretations and classifications may be influenced by the researchers' cultural backgrounds.	Expand the sample size and platforms to include more diverse groups and incorporate multiple social media platforms to enhance representativeness and comprehensiveness.<br>Increase interdisciplinary research, combining education, sociology, and legal studies to explore the boundaries between online incivility and professional conduct norms, addressing the challenges of professional ethics in the digital age.<br>Conduct dynamic tracking studies and longitudinal research to monitor the impact of major social and cultural events on students' online behavior and awareness of professional norms.
**16**	Core Emerging Hotspot (X⩾ 0, Y⩾3.51, Z ⩾0.5)	covid-19	Cluster 4	Hindex_max	36092862	7.5	290	6.644677314	USA	Twitter	Covid-19; Machine Learning; Sentiment Analysis; Twitter; Vaccine Hesitancy	Qorib et. al.(2023) [16]	Covid-19 vaccine hesitancy: Text mining, sentiment analysis and machine learning on COVID-19 vaccination Twitter dataset.	Expert systems with applications	2023	This article aims to analyze changes in public attitudes toward COVID-19 vaccine hesitancy through social media data analysis. The study utilizes real-time public data from Twitter and employs three sentiment analysis methods (Azure Machine Learning, VADER,	This study improved the accuracy of COVID-19 vaccine hesitancy sentiment analysis through large-scale model comparisons and method innovations, revealing a trend toward increasingly positive public attitudes.	Data limitations: The study relies on a limited Twitter dataset, which may affect model training due to sample size constraints.<br>The combination of vectorization methods increases model complexity, leading to a decline in classification performance,	Expand the dataset scale by continuously collecting and accumulating more Twitter data to enhance model stability and generalizability.<br>Introduce deep learning models and test and compare neural network methods (such as CNN, LSTM, BERT)

																and TextBlob) along with five machine learning algorithms (Random Forest, Logistic Regression, Decision Tree, LinearSVC, and Naive Bayes) to conduct comparative experiments with three text vectorization methods (Doc2Vec, CountVectorizer, and TF-IDF). The study also designed three word normalization strategies (stem extraction, stem normalization, and a combination of both) and developed and evaluated 42 models for each strategy.		suggesting that further optimization is needed in feature engineering.<br>The scope of sentiment classification is limited, with the study primarily focusing on three categories of sentiment (positive, neutral, and negative), without delving into more granular sentiments or thematic semantics.	in the future to improve classification performance and understanding of complex semantics.<br>Cross-model optimization: Further explore the optimal configuration of different vectorization and model combinations to find a balance between accuracy and computational complexity.<br>Apply the improved model to public health research to provide real-time public opinion monitoring tools for policymakers and public health communication.
**16**	Core Emerging Hotspot (X⩾0, Y⩾3.51, Z⩾0.5)	twitter	Cluster 4	Hindex_max，RCR_max	36092862	7.5	290	6.644677314	USA	Twitter	Covid-19; Machine Learning; Sentiment Analysis; Twitter; Vaccine Hesitancy	Qorib et. al.(2023) [16]	Covid-19 vaccine hesitancy: Text mining, sentiment analysis and machine learning on COVID-19 vaccination Twitter dataset.	Expert systems with applications	2023	This article aims to analyze changes in public attitudes toward COVID-19 vaccine hesitancy through social media data analysis. The study utilizes real-time public data from Twitter and employs three sentiment analysis methods (Azure Machine Learning, VADER, and TextBlob) along with five machine learning algorithms (Random Forest, Logistic Regression, Decision Tree, LinearSVC, and Naive Bayes) to conduct comparative experiments with three text vectorization methods (Doc2Vec, CountVectorizer, and TF-IDF). The study also designed three word normalization strategies (stem extraction, stem normalization, and a combination of both) and developed and evaluated 42 models for each strategy.	This study improved the accuracy of COVID-19 vaccine hesitancy sentiment analysis through large-scale model comparisons and method innovations, revealing a trend toward increasingly positive public attitudes.	Data limitations: The study relies on a limited Twitter dataset, which may affect model training due to sample size constraints.<br>The combination of vectorization methods increases model complexity, leading to a decline in classification performance, suggesting that further optimization is needed in feature engineering.<br>The scope of sentiment classification is limited, with the study primarily focusing on three categories of sentiment (positive, neutral, and negative), without delving into more granular sentiments or thematic semantics.	Expand the dataset scale by continuously collecting and accumulating more Twitter data to enhance model stability and generalizability.<br>Introduce deep learning models and test and compare neural network methods (such as CNN, LSTM, BERT) in the future to improve classification performance and understanding of complex semantics.<br>Cross-model optimization: Further explore the optimal configuration of different vectorization and model combinations to find a balance between accuracy and computational complexity.<br>Apply the improved model to public health research to provide real-time public opinion monitoring tools for policymakers and public health communication.
**17**	Exploratory Emerging Theme (X<0, Y<3.51, Z⩾0.5)	quality of life	Cluster 2	IF_max，Hindex_max，RCR_max	32837290	2.5	120	0.231672782	Saudi Arabia		Quality of life; Smart cities; Smart villages; Social media mining; Soft computing; Well-being	Lytras et. al.(2020) [17]	Social media mining for smart cities and smart villages research.	Soft computing	2020	This is an editorial article. The article emphasizes how social media can be used to improve citizens' well-being and quality of life in the development of smart cities and smart villages. The editorial introduces the application of social media data combined with soft computing methods (fuzzy logic, neural networks, evolutionary computing, etc.) in smart city governance, including citizen participation, infrastructure monitoring, policy making, sustainability, and social trust building. This article summarizes and showcases the results of the papers included in this special issue, including network violence detection, cultural identity quantification, public opinion and stock market prediction, and recommendation system construction, illustrating the broad potential of social media mining.	The contribution of this editorial lies in proposing an interdisciplinary agenda for integrating social media mining with smart city/rural studies, and demonstrating the potential and application cases of soft computing methods.	The limitations include the early stage of the research and the inadequacy of data and methods.	Deepening method integration by further combining artificial intelligence, deep learning, graph neural networks, and social media mining for more complex pattern recognition and prediction.<br>Combine cross-platform and multimodal data, integrating IoT, sensor data, and image/text/voice data to build a multi-source data-driven research framework.
**18**	Exploratory Emerging Theme (X<0, Y<3.51, Z⩾0.5)	social media data	Cluster 6	IF_max，Hindex_max，RCR_max	34751239	12	156	0.939062946	USA	Google Maps reviews	COVID-19 policy; Human-building interations; Human-centric urban infrastructures; Public perception; Social media data; Text mining	Park et. al.(2022) [18]	Toward human-centric urban infrastructure: Text mining for social media data to identify the public perception of COVID-19 policy in transportation hubs.	Sustainable cities and society	2022	This paper examines the impact of the COVID-19 pandemic on public health safety at transportation hubs, with a particular focus on airports as high-risk locations. The research team employed text mining methods to collect and analyze 103,428 Google Maps reviews from 64 major U.S. hub airports, identifying core issues of concern to travelers at airports and conducting	This study contributes by proposing a text mining-based social media analysis framework, revealing the critical role of airport spaces and services in compliance with pandemic policies.	Insufficient data representativeness: The study only used Google Maps reviews and did not include other platforms (such as Twitter and Instagram), and excluded individuals who rarely express opinions on social media, which may affect the generalizability of the results.<br>Limitations of spatial analysis methods: The Space Syntax method used is based on random	Data fusion across multiple platforms: Combine data from additional social media platforms such as Twitter and Instagram to enhance the representativeness and robustness of research findings.<br>Conduct cross-population studies to incorporate the opinions of more diverse groups (e.g., those who rarely voice their opinions on social media) to reduce sample bias.

																further in-depth analysis and discussion based on the review content.		movement patterns, making it difficult to accurately simulate passenger behavior in the directed, dynamic environment of an airport, thereby limiting its ability to predict spatial configuration responses.<br>□ Limited generalizability of results: Despite the large dataset, the single source of samples limits the generalizability of the findings, which requires further validation.	□ Optimize spatial analysis by developing or adopting more suitable simulation methods for dynamic spaces to more accurately capture the impact of events or policies.<br>□ Further explore how to directly integrate text mining results into facility management and policy evaluation processes to establish a data-driven mechanism for improving health and safety.
**19**	Potential Breakthrough Theme (X<0, Y⩾3.51, Z⩾ 0.5)	attitude	Cluster 3	RCR_max	33617460	6	214	0.995224213	China	Twitter	COVID-19; Twitter mining; concerns; disparities; infodemiology; infoveillance; pandemic; population groups; sentiments	Zhang et. al.(2021) [19]	Understanding Concerns, Sentiments, and Disparities Among Population Groups During the COVID-19 Pandemic Via Twitter Data Mining: Large-scale Cross-sectional Study.	Journal of medical Internet research	2021	This study focuses on the differences in concerns and emotions among different groups during the COVID-19 pandemic. The authors used large-scale Twitter data mining to conduct information monitoring (infoveillance) and completed the study in three steps: first, they collected and preprocessed 1,015,655 original English tweets from August 7 to 12, 2020. Second, they constructed an analytical framework simulating a cross-sectional questionnaire through Twitter data mining. The core of this framework consists of four intelligent modules: the first is a demographic feature extractor, which uses the M3 multimodal deep neural network model to infer user types based on user avatars, usernames, and profiles. The second is a concern classifier, which categorizes tweets into five major categories—economic, political, health, education, and entertainment—using a custom dictionary and regular expression matching. The third is a sentiment analyzer, which uses the VADER sentiment analysis model to classify tweets into negative, neutral, and positive. The fourth is an emotion detector, which integrates three emotion theory frameworks: the Ekman six-basic emotion model, Plutchik's eight-primary emotion wheel, and the POMS six-emotion state scale, based on a character-level RNN-trained Twitter emotion recognition model. Finally, the study conducted a cross-sectional analysis and validation, using the odds ratio (OR) to compare the differences in concerns and emotions across different populations under multiple variables, and the chi-square test to assess the significance level of these differences.	This study provides a fast, efficient, and cost-effective method for analyzing population differences in public health events. Additionally, the research team has released the open-source code on GitHub, facilitating reuse or expansion by academia and practice sectors for pandemic evolution tracking or other public health emergency research, enabling further utilization and expansion by academia and public health departments.	□ Limited extraction of demographic characteristics: The algorithm can only identify three attributes—user type, gender, and age—limiting more complex, multidimensional analyses of population differences.<br>□ Age categorization is not sufficiently refined, making it difficult to reveal differences within the elderly population.<br>□ Compared to traditional questionnaires, there are shortcomings in terms of multivariate control and subgroup analysis.	□ Optimize the demographic feature extraction algorithm to include additional attributes (e.g., occupation, region, education level) and refine age group classifications to enhance the granularity of analysis.<br>□ Expand to cross-scenario applications by applying this method and open-source tools to other public health emergencies to test their applicability in different contexts.<br>□ Conduct long-term tracking studies to use this tool for dynamic monitoring of the pandemic's subsequent evolution, analyze changes in population needs and emotions over time, and provide continuous data support for public health decision-making.
**20**	Potential Breakthrough Theme (X<0, Y⩾3.51, Z⩾ 0.5)	vaccine	Cluster 6	RCR_max	34665761	3.9	64	1.242361667	USA	Twitter	COVID-19; LDA; Twitter; share; social media; topic modeling; vaccine; valence; viral	Zhang et. al.(2021) [20]	Factors Driving the Popularity and Virality of COVID-19 Vaccine Discourse on Twitter: Text Mining and Data Visualization Study.	JMIR public health and surveillance	2021	This study focuses on the dissemination mechanisms of COVID-19 vaccine-related tweets, exploring which factors drive the popularity and virality of information on social media. The authors collected 501,531 publicly available English-language tweets posted in the United	This study not only reveals the key factors influencing the spread of COVID-19 vaccine-related public opinion but also provides new insights into the diffusion patterns of public health information on social media. The contributions of this paper lie in	□ Limited identification of factors, relying on automated text mining, and lacking in-depth analysis of complex factors such as political polarization and cognitive motives (e.g., self-presentation, identity expression).<br>□ Data scope limitations: The	□ Conduct research on political polarization and cognitive mechanisms to further explore the influence of psychological and social factors.<br>□ Conduct cross-platform comparisons to examine differences in driving factors across different platform characteristics.

																States between January 2020 and April 2021. They used a latent Dirichlet allocation (LDA) model to perform topic modeling and sentiment analysis to extract underlying topics and emotional tendencies.<br>Combining media content, linguistic features, and account verification information, they employed regression models to predict the likelihood of tweets being liked and retweeted.<br>Additionally, the study conducted network analysis and visualization of the 2,500 most liked and most retweeted tweets to identify distinct thematic communities and their interconnections.	identifying the thematic and informational drivers of vaccine-related tweet propagation and offering practical implications for public health communication.	analysis was confined to publicly available Twitter posts within the United States, limiting generalizability to other countries and social media platforms.<br>Potential confounders were not included; social media bots were not identified or excluded, which may have introduced bias into the results.	Analyze social bots, introduce bot detection methods, and study their actual impact on vaccine-related public opinion dissemination and information diffusion.<br>Conduct cross-national studies to expand to other countries and language environments.
**21**	Potential Breakthrough Theme (X<0, Y⩾3.51, Z⩾ 0.5)	vaccine	Cluster 6	IF_max	35085399	6.4	77	0.950185696	France	HolidayCheck.de, TripAdvisor.de, Lonelyplanet.de, Stefan-Loose.de, GeoForum.de & ReiseFrage.net	Big Data; health-seeking behaviours; social media listening; traveller personas; vaccines; visiting friends and relatives; web scraping	Bravo et. al.(2022) [21]	Using social media listening and data mining to understand travellers' perspectives on travel disease risks and vaccine-related attitudes and behaviours.	Journal of travel medicine	2022	This study aims to explore German travelers' attitudes and behaviors toward vaccines and their methods of obtaining travel-related health information. This study combines web scraping and online questionnaires to analyze German travelers' attitudes and behaviors toward vaccines and health information. The research team crawled text data from major German travel websites and used natural language processing technology to convert unstructured comments into structured features. Subsequently, through online questionnaires conducted on Facebook and Twitter, traveler profiles were defined based on K-means clustering and mapped to the crawled data. Finally, IBM Watson Tone Analyzer was used to perform sentiment analysis on texts related to infectious diseases to assess the emotional responses of different traveler groups.	This study innovatively employs social media data mining to investigate travelers' health perceptions and vaccine attitudes, providing insights for personalized travel medicine consultations and public health interventions.	The sample lacks representativeness, as the survey only covers individuals with internet access and active social media use, with limited samples from certain age groups, thereby limiting the generalizability of the results.<br>Data collected through web scraping may contain missing values and biases.<br>External factors were not included, and other potential influences from contextual and environmental factors were not observed.	Conduct longitudinal studies, such as post-pandemic research, by repeating similar studies after the COVID-19 pandemic to compare the health concerns, anxiety levels, and behavioral differences of travelers before and after the pandemic.<br>Expand the scope of data to include multi-dimensional information, such as mobile technology and spatial data, to accurately assess health risks.<br>Include more diverse groups to enhance representativeness and improve the comprehensiveness of the study.<br>Explore practical applications, such as integrating social media analysis results with travel medicine consultations as a complementary rather than substitute approach to enhance the targeting and effectiveness of health risk communication.<br>Research the application of social media in public health, and further investigate the role of social media in monitoring misinformation and disseminating health guidance during health crises.
**22**	Potential Breakthrough Theme (X<0, Y⩾3.51, Z⩾ 0.5)	content analysis	Cluster 5	RCR_max，Hindex_max	36780224	6	214	2.664714409	USA	Facebook	Facebook; content analysis; engagement; natural language processing; public health; smoking; social media; social media campaign; tobacco; tobacco control; topic modeling; use; youth	Lin et. al.(2023) [22]	Social Media Data Mining of Antitobacco Campaign Messages: Machine Learning Analysis of Facebook Posts.	Journal of medical Internet research	2023	This study focuses on the effectiveness of anti-tobacco campaigns on social media, particularly exploring how different information frameworks influence public interaction and engagement. The research team collected 3,515 posts and 28,125 comments from seven large-scale national and local anti-tobacco Facebook campaigns between 2018 and 2021.<br>Methodologically, the study first used Facebook Scraper and manual methods to obtain posts and comments, extracting metadata such as text, emojis, posting times, and video information. Second, the VADER algorithm was used to conduct sentiment analysis on posts and comments, categorizing text polarity into positive, neutral, and negative, with manual verification conducted on a portion of the data. Third, the study used a logistic regression model to	By integrating machine learning, natural language processing, and traditional content analysis, this study provides empirical evidence for understanding the effectiveness and optimization strategies of digital anti-tobacco campaigns.	Cross-sectional design cannot track individual behavior changes over time.<br>Platform and sample limitations: The analysis was limited to a portion of large-scale educational campaigns and focused solely on Facebook, potentially limiting the generalizability of the results to other platforms or populations.<br>Algorithmic limitations: Sentiment analysis tools struggle to identify ironic contexts, potentially leading to biases.<br>The study ignores the inherent correlations within the data, although clustering methods were used to partially control for this, potential influences may still exist.	Conduct longitudinal research designs to examine behavioral changes.<br>Conduct cross-platform comparisons by integrating data from multiple platforms such as Twitter, Instagram, and YouTube to expand the sample size and compare user response differences across platforms.<br>Apply advanced NLP models, adopting more cutting-edge context-aware NLP models (e.g., BERT) to enhance the accuracy of sentiment and topic recognition, particularly in special and complex semantic detection tasks such as sarcasm detection.

																examine the relationship between posts with different emotional frames (positive, negative, neutral) and user responses (comment sentiment, number of likes, number of shares, number of comments), while controlling for variables such as time and video content. Finally, in terms of topic mining, the study selected posts with the highest interaction volume and employed traditional content analysis (conducted independently by two coders) and LDA topic modeling to identify the most representative anti-tobacco topics from the text.			
**23**	Potential Breakthrough Theme (X<0, Y⩾3.51, Z⩾ 0.5)	content analysis	Cluster 5	Hindex_max	39847418	6	214		Republik China (Taiwan)	Facebook	content analysis; fact-checking; fuzzy-trace theory; health communication; health misinformation; large language models; misinformation correction; social media; text mining; user engagement	Kuo & Chen (2025) [23]	Predicting User Engagement in Health Misinformation Correction on Social Media Platforms in Taiwan: Content Analysis and Text Mining Study.	Journal of medical Internet research	2025	This study examines the dissemination and user engagement patterns of health misinformation correction posts on social media, using Facebook data from the three years of the COVID-19 pandemic. The research objectives are to identify the content characteristics of different types of correction posts, analyze their relationship with user interactions (shares, reactions, comments), and explore how the cognitive and emotional information in comments further influence user engagement. Methodologically, the study first collected 1,424 correction posts and 67,378 comments from the Facebook pages of the Taiwan Fact-Checking Center (TFC) and the Ministry of Health and Welfare (MOHW) between January 2020 and December 2022. The study employed a combination of manual screening and keyword filtering to ensure the sample exclusively contained content related to health misinformation. Second, the posts were manually annotated based on fuzzy-trace theory, categorizing them into "core information" (risk, knowledge enhancement, value orientation) and "literal information" (numbers, authority, facts), while simultaneously extracting control variables such as post length, readability, tone, and whether images were included. Subsequently, the comment section was labeled using a combination of GPT-3.5 automatic labeling and manual sampling verification, categorizing comments into cognitive engagement (knowledge-based, critical, biased) and emotional engagement (positive, negative, neutral), with classification accuracy improved through multiple rounds of manual and model comparison. Finally, the study utilized linear regression and log-transformed models to analyze the relationships between	This comprehensive method, which combines big data scraping, manual annotation, AI automatic classification, and statistical modeling, provides a new empirical foundation for understanding the dissemination effects of health misinformation corrections and designing more effective public health communication strategies.	Timeliness constraints: The study is based on three years of COVID-19 pandemic data, and its conclusions require validation for other health crises.<br>Automatic annotation bias: GPT-3.5 exhibits misclassification in certain cultural contexts, leading to inaccurate categorization of some comments.<br>Unclear variable explanations lead to confusion in interpreting the results.<br>Image factors were not disaggregated; the study only considered whether posts contained images, without analyzing image content or whether they conveyed key messages.	Optimize the classification system by simplifying comment participation types (e.g., merging knowledge-based and critical types, positive and neutral emotions) to improve classification accuracy and practicality.<br>Conduct in-depth comment analysis to systematically extract user feedback on post design (e.g., criticism of lack of focus, praise for practicality) to assess the acceptability of corrective information.<br>Numerical information framework research: Further explore how to more effectively present numerical information such as vaccine adverse reaction rates to avoid misunderstandings and improve dissemination effectiveness.<br>Future research should explore the optimal combination of multiple attributes within a single post to investigate the effects of multi-attribute combinations and determine which combinations most effectively enhance user engagement.<br>Expand cross-contextual applications by extending this research framework to non-pandemic scenarios or other health-related issues to validate its universality and robustness.

																post attributes, comment categories, and user engagement metrics (number of shares, reactions, and comments).			
**24**	Potential Breakthrough Theme (X<0, Y⩾3.51, Z⩾ 0.5)	attitude	Cluster 3	IF_max、Hindex_max	39869893	6	214		Italy	Twitter	COVID-19; Egypt; Italy; LDA; SARS-CoV-2; UK; attitude; citizen opinion; content analysis; dataset; developing economies; health crisis; infectious; latent Dirichlet allocation; machine learning; pandemic; perception; perspective; public health; sentiment; social media; text mining; tweet; twitter; vaccination; vaccine	Kahlawi et. al.(2025) [24]	Cross-Cultural Sense-Making of Global Health Crises: A Text Mining Study of Public Opinions on Social Media Related to the COVID-19 Pandemic in Developed and Developing Economies.	Journal of medical Internet research	2025	This study aims to explore how citizens engage in collective sense-making during the COVID-19 pandemic across different cultural and economic contexts. The study focuses on Italy, the United Kingdom, and Egypt, which represent different pandemic response and social contexts, with the goal of revealing the evolutionary characteristics and differences of public discourse on social media. Methodologically, the study collected a total of 755,215 Twitter posts covering three key periods: the initial phase of the virus outbreak (February 15–March 31, 2020), the strict lockdown period (April 1–May 30, 2020), and the vaccine rollout phase (December 1, 2020–January 15, 2021). Data collection utilized the Python Tweepy library, employing keyword strategies and geocoding filters to ensure posts met language and geographic criteria. Each post must contain at least one keyword related to the pandemic. Datasets were established separately for the three countries and three periods, ultimately forming nine independent corpora. Text analysis was conducted using the Latent Dirichlet Allocation (LDA) model, combined with a bigram model to enhance semantic coherence. The analysis process comprised three steps: ① Data preparation, where each post was treated as a document; ② Determining the optimal number of themes, achieved through multiple experiments to extract 2–10 themes and calculate consistency values, selecting the optimal model; ③ Extracting topic keywords and manually annotating them by experts to ensure the accuracy of topic interpretation. LDA ultimately outputs the vocabulary distribution of each topic and its proportion in the overall corpus, thereby revealing the main topics and concerns of the public in different countries at different stages.	This method, which combines cross-national large-scale data collection, machine learning topic modeling, and human verification, effectively captures the meaning-making process of citizens in different contexts during the pandemic and provides empirical references for future cross-cultural comparisons and crisis communication studies in public crises.	Limitations of single-platform data.<br>Data timeliness limitations: The data only covers the early stages of the pandemic, lockdown periods, and the initial phase of vaccine rollout, failing to capture changes in meaning construction during the long-term evolution of the pandemic.<br>Methodological limitations: While topic modeling can reveal macro-level themes, it struggles to capture the nuanced narratives and context-dependent nuances at the individual level, potentially leading to simplifications of discourse diversity.<br>Cultural interpretation biases: Although the study involves Italy, the UK, and Egypt, explanations of complex cultural factors may still be incomplete.	Multi-platform data integration.<br>Expanding the temporal scope and conducting longitudinal studies.<br>Methodological refinement: Combining sentiment analysis, semantic networks, and qualitative analysis to more comprehensively capture citizens' narratives and emotional expressions during crises.<br>Cultural and contextual studies to systematically explore the role of cultural and other factors in meaning construction and integrate these findings with health communication strategies.
**25**	Immature but Declining Theme (X<0, Y⩾3.51, Z<0.5)	tweet	Cluster 4	IF_max、Hindex_max	37428522	6	214	1.241582407	USA	Twitter	COVID-19; Twitter; assessment; diet; dieticians; food; food groups; immune system; immunity; immunity building; nutrition; nutrition discourse; nutritionists; social media; text mining; tweets	Shankar et. al.(2023) [25]	Investigating the Role of Nutrition in Enhancing Immunity During the COVID-19 Pandemic: Twitter Text-Mining Analysis.	Journal of medical Internet research	2023	This study focuses on the public's perceptions and attitudes toward nutrition and immunity on Twitter during the COVID-19 pandemic, aiming to reveal how the general public understands and responds to health crises through food and diet. In terms of methodology, the study first used the TWINT tool to collect 71,178 nutrition-related tweets from January 1 to	This method combines large-scale social media data collection, preprocessing, semi-supervised topic modeling, and sentiment analysis to systematically reveal the public's focus and emotional tendencies toward nutrition-related topics during the pandemic, providing empirical support for health communication and	The limitation of relying on a single platform reduces the generalizability of the results.<br>Insufficient population representativeness and lack of analysis of users' demographic characteristics.<br>Lack of geographical variation prevents exploration of differences in user perspectives on nutrition-related topics across regions, limiting cross-cultural	Combine data from multiple platforms to obtain a more comprehensive overview of social media discussions.<br>Conduct population segmentation studies, incorporating demographic analysis of users, or compare the distinct discussion patterns between professional groups and the general public.<br>Compare regional differences to study variations in nutrition-

																September 30, 2020, avoiding the time constraints of the Twitter API. Subsequently, the study conducted rigorous data preprocessing: using Stanford NLP's stemming algorithm to unify synonyms, removing stop words and invalid characters to ensure the analysis focused on meaningful textual features. Following this, the study employed the Correlation Explanation (CorEx) semi-supervised topic modeling algorithm, combining the U.S. Department of Agriculture (USDA) food classification system and keywords as "anchor words" to automatically identify the main topics in the tweets. Each tweet was categorized into the most relevant topic based on topic strength, and the importance of each topic was calculated. Finally, the study utilized the VADER sentiment analysis tool to classify tweets into positive, negative, or neutral sentiment polarities and combined qualitative analysis to further understand public attitudes toward different topics.	public policy making.	and regional understanding.<br>Limitations in keywords: The keywords used for data collection may be incomplete, potentially omitting some relevant discussions.	related tweets across different countries or regions, and understand how cultural and geographical backgrounds influence topic selection and attitudes.<br>Optimize methodologies by experimenting with BERTopic (BERT-based topic modeling) to enhance semantic understanding of potential topic identification and better integrate human input with domain knowledge.

References

1. Park A, Conway M, Chen AT. Examining Thematic Similarity, Difference, and Membership in Three Online Mental Health Communities from Reddit: A Text Mining and Visualization Approach. Comput Human Behav 2018;78:98-112. PMID:29456286
2. Li J, Xu Q, Cuomo R, Purushothaman V, Mackey T. Data Mining and Content Analysis of the Chinese Social Media Platform Weibo During the Early COVID-19 Outbreak: Retrospective Observational Infoveillance Study. JMIR Public Health Surveill 2020;6(2):e18700. PMID:32293582
3. Daughton AR, Shelley CD, Barnard M, Gerts D, Watson Ross C, Crooker I, Nadiga G, Mukundan N, Vaquera Chavez NY, Parikh N, Pitts T, Fairchild G. Mining and Validating Social Media Data for COVID-19-Related Human Behaviors Between January and July 2020: Infodemiology Study. J Med Internet Res 2021;23(5):e27059. PMID:33882015
4. Svenstrup D, Jørgensen HL, Winther O. Rare disease diagnosis: A review of web search, social media and large-scale data-mining approaches. Rare Dis 2015;3(1):e1083145. PMID:26442199
5. Dai H, Hao J. Mining social media data for opinion polarities about electronic cigarettes. Tob Control 2017;26(2):175-180. PMID:26980151
6. Lazard AJ, Saffer AJ, Wilcox GB, Chung AD, Mackert MS, Bernhardt JM. E-Cigarette Social Media Messages: A Text Mining Analysis of Marketing and Consumer Conversations on Twitter. JMIR Public Health Surveill 2016;2(2):e171. PMID:27956376
7. Lazard AJ, Wilcox GB, Tuttle HM, Glowacki EM, Pikowski J. Public reactions to e-cigarette regulations on Twitter: a text mining analysis. Tob Control 2017;26(e2):e112-e116. PMID:28341768
8. Subirats L, Reguera N, Bañón AM, Gómez-Zúñiga B, Minguillón J, Armayones M. Mining Facebook Data of People with Rare Diseases: A Content-Based and Temporal Analysis. Int J Environ Res Public Health 2018;15(9). PMID:30200209
9. Di Wang, Lyu JC, Zhao X. Public Opinion About E-Cigarettes on Chinese Social Media: A Combined Study of Text Mining Analysis and Correspondence Analysis. J Med Internet Res 2020;22(10):e19804. PMID:33052127
10. Cheng Q, Li TM, Kwok C-L, Zhu T, Yip PS. Assessing Suicide Risk and Emotional Distress in Chinese Social Media: A Text Mining and Machine Learning Study. J Med Internet Res 2017;19(7):e243. PMID:28694239
11. Lam CS, Zhou K, Loong HH-F, Chung VC-H, Ngan C-K, Cheung YT. The Use of Traditional, Complementary, and Integrative Medicine in Cancer: Data-Mining Study of 1 Million Web-Based Posts From Health Forums and Social Media Platforms. J Med Internet Res 2023;25:e45408. PMID:37083752
12. Nikfarjam A, Sarker A, O'Connor K, Ginn R, Gonzalez G. Pharmacovigilance from social media: mining adverse drug reaction mentions using sequence labeling with word embedding cluster features. J Am Med Inform Assoc 2015;22(3):671-681. PMID:25755127
13. Blumenthal KG, Topaz M, Zhou L, Harkness T, Sa'adon R, Bar-Bachar O, Long AA. Mining social media data to assess the risk of skin and soft tissue infections from allergen immunotherapy. J Allergy Clin Immunol 2019;144(1):129-134. PMID:30721764
14. Ayyoubzadeh SM, Ayyoubzadeh SM, Zahedi H, Ahmadi M, R Niakan Kalhori S. Predicting COVID-19 Incidence Through Analysis of Google Trends Data in Iran: Data Mining and Deep Learning Pilot Study. JMIR Public Health Surveill 2020;6(2):e18828. PMID:32234709
15. Gagne JC de, Cho E, Yamane SS, Jin H, Nam JD, Jung D. Analysis of Cyberincivility in Posts by Health Professions Students: Descriptive Twitter Data Mining Study. JMIR Med Educ 2021;7(2):e28805. PMID:33983129
16. Qorib M, Oladunni T, Denis M, Ososanya E, Cotae P. Covid-19 vaccine hesitancy: Text mining, sentiment analysis and machine learning on COVID-19 vaccination Twitter dataset. Expert Syst Appl 2023;212:118715. PMID:36092862
17. Lytras MD, Visvizi A, Jussila J. Social media mining for smart cities and smart villages research. Soft comput 2020;24(15):10983-10987. PMID:32837290
18. Park JY, Mistur E, Kim D, Mo Y, Hoefer R. Toward human-centric urban infrastructure: Text mining for social media data to identify the public perception of COVID-19 policy in transportation hubs. Sustain Cities Soc 2022;76:103524. PMID:34751239
19. Zhang C, Xu S, Li Z, Hu S. Understanding Concerns, Sentiments, and Disparities Among Population Groups During the COVID-19 Pandemic Via Twitter Data Mining: Large-scale Cross-sectional Study. J Med Internet Res 2021;23(3):e26482. PMID:33617460
20. Zhang J, Wang Y, Shi M, Wang X. Factors Driving the Popularity and Virality of COVID-19 Vaccine Discourse on Twitter: Text Mining and Data Visualization Study. JMIR Public Health Surveill 2021;7(12):e32814. PMID:34665761
21. Bravo C, Castells VB, Zietek-Gutsch S, Bodin P-A, Molony C, Frühwein M. Using social media listening and data mining to understand travellers' perspectives on travel disease risks and vaccine-related attitudes and behaviours. J Travel Med 2022;29(2). PMID:35085399
22. Lin S-Y, Cheng X, Zhang J, Yannam JS, Barnes AJ, Koch JR, Hayes R, Gimm G, Zhao X, Purohit H, Xue H. Social Media Data Mining of Antitobacco Campaign Messages: Machine Learning Analysis of Facebook Posts. J Med Internet Res 2023;25:e42863. PMID:36780224
23. Kuo H-Y, Chen S-Y. Predicting User Engagement in Health Misinformation Correction on Social Media Platforms in Taiwan: Content Analysis and Text Mining Study. J Med Internet Res 2025;27:e65631. PMID:39847418
24. Kahlawi A, Masri F, Ahmed W, Vidal-Alaball J. Cross-Cultural Sense-Making of Global Health Crises: A Text Mining Study of Public Opinions on Social Media Related to the COVID-19 Pandemic in Developed and Developing Economies. J Med Internet Res 2025;27:e58656. PMID:39869893
