# Supplementary material for: Thematic Mapping and Evolution of Social Media Mining in Health Research: Hybrid Bibliometric Synthesis
Source: J Med Internet Res. 2026 May 8;28:e86200. doi: 10.2196/86200 (PMC13160668; doi:10.2196/86200)
Supplement: Multimedia Appendix 6 [file jmir-v28-e86200-s006.pdf]

Content of Multimedia Appendix

Multimedia Appendix 6. Supplementary tables to support the study ... 2

Table S1. ARIMA-Based Exploratory Projection and Baseline Comparisons for Annual Publication Counts (2015–2025) ... 2

Table S2. Frequency of Co-authorship Between Author Pairs and Associated PubMed Publications ... 2

Table S3. Details of Author Collaborations and Associated PubMed Publications ... 4

Table S4. Statistics of Centroid Distances for Six Clusters ... 5

Table S5. Cross-Cluster Strength between Thematic Clusters Underlying the Inter-cluster Coupling Heatmap ... 5

Table S6. Full List of Inter-Cluster Pairs Ranked by Cross-Cluster Strength ... 5

Table S7. Full List of Pair-Specific Bridging Keywords and Keyword–Pair Contributions Across All Inter-Cluster Pairs ... 6

Table S8. Top 30 Overall Bridging Keywords and Their Cross-Cluster Strengths ... 7

Table S9. Sensitivity Analysis of Burst Detection Parameter Grid (Kleinberg model across γ, slice width, and minimum burst length) ... 8

Table S10. Internal validation metrics across clustering configurations (UMAP + HDBSCAN with multiple profiles and K values) ... 8

Multimedia Appendix 6. Supplementary tables to support the study

Table S1. ARIMA-Based Exploratory Projection and Baseline Comparisons for Annual Publication Counts (2015–2025)

Metric	Value
ARIMA Order	(1,1,1)
AIC	73.46
BIC	74.37
LogLikelihood	-33.73
2025 Predicted	32
2025 Predicted 95% CI Lower	18
2025 Predicted 95% CI Upper	45
2025 Actual (to 7/31)	15
Baseline Mean (2015–2024)	17.40
Baseline Linear Trend Pred2025 (fit on 2015–2024)	32.2
Baseline Naive (2024 carry-forward)	17

The annual publication-count time series was fitted on 2015–2024 using an ARIMA(1,1,1) model to generate a one-step-ahead exploratory projection for 2025. Model fit statistics (AIC, BIC, and log-likelihood) are reported for reference. The projected 2025 count was 32 with a 95% prediction interval of 18–45. 15 publications had been recorded by July 31, 2025. Given the short annual series, three baselines are provided for contextual comparison: the 2015–2024 mean (17.40), a linear-trend projection for 2025 fitted on 2015–2024 (32.2), and a naïve carry-forward baseline using the 2024 value (17). Thus, the ARIMA model serves only as an exploratory projection, yielding a broad prediction interval. The predicted counts for year 2025 should be interpreted with caution.

Table S2. Frequency of Co-authorship Between Author Pairs and Associated PubMed Publications

Author 1	Author 2	Co-occurrence	Number of Publications for Author Pair	PubMed IDs
Davy Weissenbacher	Graciela Gonzalez-Hernandez	4	4	30292855; 32634878; 33594374; 34270701
Allison J Lazard	Gary B Wilcox	3	3	26138998; 27956376; 28341768
Chunyan Zhang	Songhua Xu	2	2	33617460; 40053739
Adel Mebarki	Joelle Malaab	2	2	36447795; 37143935
Chunyan Zhang	Zongfang Li	2	2	33617460; 40053739
Joelle Malaab	Paul Loussikian	2	2	36447795; 37143935
Jonathan Koss	Sabine Bohnet-Joschko	2	2	34481080; 36007131
Adel Mebarki	Paul Loussikian	2	2	36447795; 37143935
Nathalie Texier	Pierre Foulquié	2	2	29881351; 36447795
Ari Z Klein	Graciela Gonzalez-Hernandez	2	2	30292855; 38218723
Arjun Magge	Davy Weissenbacher	2	2	33594374; 34270701
Frada Burstein	Pari Delir Haghighi	2	2	32883653; 35708760
Anne Dirkson	Suzan Verberne	2	2	34270701; 36309197
Songhua Xu	Zongfang Li	2	2	33617460; 40053739
Adel Mebarki	Stéphane Schück	2	2	37143935; 40289322
Abeed Sarker	Ari Z Klein	2	2	30292855; 38218723
Paméla Voillot	Stéphane Schück	2	2	34255645; 40289322
Gerard Van Oortmerssen	Hans Gelderblom	2	2	35233640; 36309197
Abeed Sarker	Graciela Gonzalez-Hernandez	2	2	30292855; 38218723
Gerard Van Oortmerssen	Suzan Verberne	2	2	35233640; 36309197
Gerard Van Oortmerssen	Wessel Kraaij	2	2	35233640; 36309197
Hans Gelderblom	Suzan Verberne	2	2	35233640; 36309197
Hans Gelderblom	Wessel Kraaij	2	2	35233640; 36309197
Suzan Verberne	Wessel Kraaij	2	2	35233640; 36309197
Arjun Magge	Graciela Gonzalez-Hernandez	2	2	33594374; 34270701
Jennie C De Gagne	Sandra S Yamane	2	2	30321747; 33983129
Davy Weissenbacher	Karen O'Connor	2	2	32634878; 33594374
Graciela Gonzalez-Hernandez	Karen O'Connor	2	2	32634878; 33594374
Elizabeth M Glowacki	Gary B Wilcox	2	2	28341768; 32970973
Omar El-Gayar	Tareq Nasralah	2	2	32421686; 32788147
Abeed Sarker	Haitao Cai	2	2	29084707; 30292855
Agnès Lillo-Le-Louët	Anita Burgun	2	2	29295108; 29881351
Agnès Lillo-Le-Louët	Armelle Guenegou-Arnoux	2	2	29295108; 29881351
Agnès Lillo-Le-Louët	Badisse Dahamna	2	2	29295108; 29881351
Agnès Lillo-Le-Louët	Carole Faviez	2	2	29295108; 29881351
Agnès Lillo-Le-Louët	Nathalie Texier	2	2	29295108; 29881351
Agnès Lillo-Le-Louët	Pierre Karapetiantz	2	2	29295108; 29881351
Agnès Lillo-Le-Louët	Sandrine Katsahian	2	2	29295108; 29881351
Agnès Lillo-Le-Louët	Xiaoyi Chen	2	2	29295108; 29881351
Anita Burgun	Armelle Guenegou-Arnoux	2	2	29295108; 29881351
Anita Burgun	Badisse Dahamna	2	2	29295108; 29881351
Anita Burgun	Carole Faviez	2	2	29295108; 29881351
Anita Burgun	Nathalie Texier	2	2	29295108; 29881351
Anita Burgun	Pierre Karapetiantz	2	2	29295108; 29881351
Anita Burgun	Sandrine Katsahian	2	2	29295108; 29881351
Anita Burgun	Xiaoyi Chen	2	2	29295108; 29881351
Armelle Guenegou-Arnoux	Badisse Dahamna	2	2	29295108; 29881351
Armelle Guenegou-Arnoux	Carole Faviez	2	2	29295108; 29881351
Armelle Guenegou-Arnoux	Nathalie Texier	2	2	29295108; 29881351
Armelle Guenegou-Arnoux	Pierre Karapetiantz	2	2	29295108; 29881351
Armelle Guenegou-Arnoux	Sandrine Katsahian	2	2	29295108; 29881351
Armelle Guenegou-Arnoux	Xiaoyi Chen	2	2	29295108; 29881351
Badisse Dahamna	Carole Faviez	2	2	29295108; 29881351
Badisse Dahamna	Nathalie Texier	2	2	29295108; 29881351
Badisse Dahamna	Pierre Karapetiantz	2	2	29295108; 29881351
Badisse Dahamna	Sandrine Katsahian	2	2	29295108; 29881351
Badisse Dahamna	Xiaoyi Chen	2	2	29295108; 29881351
Carole Faviez	Nathalie Texier	2	2	29295108; 29881351
Carole Faviez	Pierre Karapetiantz	2	2	29295108; 29881351
Carole Faviez	Sandrine Katsahian	2	2	29295108; 29881351
Carole Faviez	Xiaoyi Chen	2	2	29295108; 29881351
Nathalie Texier	Pierre Karapetiantz	2	2	29295108; 29881351

Nathalie Texier	Sandrine Katsahian	2	2	29295108; 29881351
Nathalie Texier	Xiaoyi Chen	2	2	29295108; 29881351
Pierre Karapetiantz	Sandrine Katsahian	2	2	29295108; 29881351
Pierre Karapetiantz	Xiaoyi Chen	2	2	29295108; 29881351
Sandrine Katsahian	Xiaoyi Chen	2	2	29295108; 29881351
Abeed Sarker	Graciela Gonzalez	2	2	25755127; 29084707
Ling Yang	Yuyang Cai	2	2	32401210; 34720798
Allison J Lazard	Jay M Bernhardt	2	2	26138998; 27956376
Gary B Wilcox	Jay M Bernhardt	2	2	26138998; 27956376
Luis M Rocha	Rion Brattig Correia	2	2	32550337; 40460925

This table presents all co-author pairs identified in the study corpus, including Author 1, Author 2, the number of co-occurrences, the number of collaborative publications for each pair, and the corresponding PubMed IDs. The co-occurrence count indicates the frequency of collaboration between the pair in the network, while the number of publications reflects the strength of their joint research output.

Table S3. Details of Author Collaborations and Associated PubMed Publications

Author	Total Collaborations	Co-authors	Total Number of Collaborative Publications	PubMed IDs
Nathalie Texier	21	Adel Mebarki; Agnès Lillo-Le-Louët; Anita Burgun; Armelle Guenegou-Arnoux; Badisse Dahamna; Carole Faviez; Joelle Malaab; Paul Loussikian; Pierre Foulquié; Pierre Karapetiantz; Sandrine Katsahian; Xiaoyi Chen	3	29295108; 29881351; 36447795
Graciela Gonzalez-Hernandez	17	Abeed Sarker; Anne Dirkson; Ari Z Klein; Arjun Magge; Davy Weissenbacher; Elena Tutubalina; Haitao Cai; Juan M Banda; Karen O'Connor; Suzan Verberne	5	30292855; 32634878; 33594374; 34270701; 38218723
Anita Burgun	17	Agnès Lillo-Le-Louët; Armelle Guenegou-Arnoux; Badisse Dahamna; Carole Faviez; Nathalie Texier; Pierre Foulquié; Pierre Karapetiantz; Sandrine Katsahian; Xiaoyi Chen	2	29295108; 29881351
Armelle Guenegou-Arnoux	17	Agnès Lillo-Le-Louët; Anita Burgun; Badisse Dahamna; Carole Faviez; Nathalie Texier; Pierre Foulquié; Pierre Karapetiantz; Sandrine Katsahian; Xiaoyi Chen	2	29295108; 29881351
Agnès Lillo-Le-Louët	17	Anita Burgun; Armelle Guenegou-Arnoux; Badisse Dahamna; Carole Faviez; Nathalie Texier; Pierre Foulquié; Pierre Karapetiantz; Sandrine Katsahian; Xiaoyi Chen	2	29295108; 29881351
Xiaoyi Chen	17	Agnès Lillo-Le-Louët; Anita Burgun; Armelle Guenegou-Arnoux; Badisse Dahamna; Carole Faviez; Nathalie Texier; Pierre Foulquié; Pierre Karapetiantz; Sandrine Katsahian	2	29295108; 29881351
Sandrine Katsahian	17	Agnès Lillo-Le-Louët; Anita Burgun; Armelle Guenegou-Arnoux; Badisse Dahamna; Carole Faviez; Nathalie Texier; Pierre Foulquié; Pierre Karapetiantz; Xiaoyi Chen	2	29295108; 29881351
Pierre Karapetiantz	17	Agnès Lillo-Le-Louët; Anita Burgun; Armelle Guenegou-Arnoux; Badisse Dahamna; Carole Faviez; Nathalie Texier; Pierre Foulquié; Sandrine Katsahian; Xiaoyi Chen	2	29295108; 29881351
Carole Faviez	17	Agnès Lillo-Le-Louët; Anita Burgun; Armelle Guenegou-Arnoux; Badisse Dahamna; Nathalie Texier; Pierre Foulquié; Pierre Karapetiantz; Sandrine Katsahian; Xiaoyi Chen	2	29295108; 29881351
Badisse Dahamna	17	Agnès Lillo-Le-Louët; Anita Burgun; Armelle Guenegou-Arnoux; Carole Faviez; Nathalie Texier; Pierre Foulquié; Pierre Karapetiantz; Sandrine Katsahian; Xiaoyi Chen	2	29295108; 29881351
Pierre Foulquié	15	Adel Mebarki; Agnès Lillo-Le-Louët; Anita Burgun; Armelle Guenegou-Arnoux; Badisse Dahamna; Carole Faviez; Joelle Malaab; Nathalie Texier; Paméla Voillot; Paul Loussikian; Pierre Karapetiantz; Sandrine Katsahian; Stéphane Schück; Xiaoyi Chen	3	29881351; 34255645; 36447795
Davy Weissenbacher	14	Abeed Sarker; Anne Dirkson; Ari Z Klein; Arjun Magge; Elena Tutubalina; Graciela Gonzalez-Hernandez; Haitao Cai; Karen O'Connor; Suzan Verberne	4	30292855; 32634878; 33594374; 34270701
Suzan Verberne	12	Anne Dirkson; Arjun Magge; Davy Weissenbacher; Elena Tutubalina; Gerard Van Oortmerssen; Graciela Gonzalez-Hernandez; Hans Gelderblom; Wessel Kraaij	3	34270701; 35233640; 36309197
Abeed Sarker	12	Ari Z Klein; Arjun Magge; Davy Weissenbacher; Graciela Gonzalez; Graciela Gonzalez-Hernandez; Haitao Cai; Juan M Banda; Karen O'Connor	7	25755127; 29084707; 30292855; 31584645; 34485849; 37113382; 38218723
Arjun Magge	11	Abeed Sarker; Anne Dirkson; Davy Weissenbacher; Elena Tutubalina; Graciela Gonzalez; Graciela Gonzalez-Hernandez; Haitao Cai; Karen O'Connor; Suzan Verberne	3	29084707; 33594374; 34270701
Anne Dirkson	9	Arjun Magge; Davy Weissenbacher; Elena Tutubalina; Gerard Van Oortmerssen; Graciela Gonzalez-Hernandez; Hans Gelderblom; Suzan Verberne; Wessel Kraaij	2	34270701; 36309197
Adel Mebarki	9	Joelle Malaab; Nathalie Texier; Paméla Voillot; Paul Loussikian; Pierre Foulquié; Stéphane Schück	3	36447795; 37143935; 40289322
Joelle Malaab	7	Adel Mebarki; Nathalie Texier; Paul Loussikian; Pierre Foulquié; Stéphane Schück	2	36447795; 37143935
Stéphane Schück	7	Adel Mebarki; Joelle Malaab; Paméla Voillot; Paul Loussikian; Pierre Foulquié	3	34255645; 37143935; 40289322
Ari Z Klein	7	Abeed Sarker; Davy Weissenbacher; Graciela Gonzalez-Hernandez; Haitao Cai; Juan M Banda	2	30292855; 38218723
Paul Loussikian	7	Adel Mebarki; Joelle Malaab; Nathalie Texier; Pierre Foulquié; Stéphane Schück	2	36447795; 37143935
Hans Gelderblom	7	Anne Dirkson; Gerard Van Oortmerssen; Suzan Verberne; Wessel Kraaij	2	35233640; 36309197
Wessel Kraaij	7	Anne Dirkson; Gerard Van Oortmerssen; Hans Gelderblom; Suzan Verberne	2	35233640; 36309197
Karen O'Connor	7	Abeed Sarker; Arjun Magge; Davy Weissenbacher; Graciela Gonzalez; Graciela Gonzalez-Hernandez	3	25755127; 32634878; 33594374
Haitao Cai	7	Abeed Sarker; Ari Z Klein; Arjun Magge; Davy Weissenbacher; Graciela Gonzalez; Graciela Gonzalez-Hernandez	2	29084707; 30292855
Gerard Van Oortmerssen	7	Anne Dirkson; Hans Gelderblom; Suzan Verberne; Wessel Kraaij	2	35233640; 36309197
Gary B Wilcox	7	Allison J Lazard; Elizabeth M Glowacki; Jay M Bernhardt	4	26138998; 27956376; 28341768; 32970973
Allison J Lazard	6	Elizabeth M Glowacki; Gary B Wilcox; Jay M Bernhardt	3	26138998; 27956376; 28341768
Graciela Gonzalez	5	Abeed Sarker; Arjun Magge; Haitao Cai; Karen O'Connor	2	25755127; 29084707
Elena Tutubalina	5	Anne Dirkson; Arjun Magge; Davy Weissenbacher; Graciela Gonzalez-Hernandez; Suzan Verberne	3	34270701; 36184069; 38008241
Chunyan Zhang	4	Songhua Xu; Zongfang Li	2	33617460; 40053739
Songhua Xu	4	Chunyan Zhang; Zongfang Li	2	33617460; 40053739
Zongfang Li	4	Chunyan Zhang; Songhua Xu	2	33617460; 40053739
Paméla Voillot	4	Adel Mebarki; Pierre Foulquié; Stéphane Schück	2	34255645; 40289322
Jay M Bernhardt	4	Allison J Lazard; Gary B Wilcox	2	26138998; 27956376
Frada Burstein	3	Pari Delir Haghighi; Roee Sa'Adon	2	32883653; 35708760
Juan M Banda	3	Abeed Sarker; Ari Z Klein; Graciela Gonzalez-Hernandez	2	32634870; 38218723
Elizabeth M Glowacki	3	Allison J Lazard; Gary B Wilcox	2	28341768; 32970973
Pari Delir Haghighi	3	Frada Burstein; Roee Sa'Adon	2	32883653; 35708760
Rion Brattig Correia	2	Luis M Rocha	2	32550337; 40460925
Luis M Rocha	2	Rion Brattig Correia	2	32550337; 40460925
Jonathan Koss	2	Sabine Bohnet-Joschko	2	34481080; 36007131
Jennie C De Gagne	2	Sandra S Yamane	2	30321747; 33983129

Sandra S Yamane	2	Jennie C De Gagne	2	30321747; 33983129
Yuyang Cai	2	Ling Yang	2	32401210; 34720798
Ling Yang	2	Yuyang Cai	2	32401210; 34720798
Sabine Bohnet-Joschko	2	Jonathan Koss	2	34481080; 36007131
Omar El-Gayar	2	Tareq Nasralah	2	32421686; 32788147
Tareq Nasralah	2	Omar El-Gayar	2	32421686; 32788147
Roee Sa'Adon	2	Frada Burstein; Pari Delir Haghighi	2	30721764; 32883653

This table summarizes the collaboration profiles of each author in the study corpus, including total collaborations, list of co-authors, the total number of collaborative publications with their most frequent collaborator, and the associated PubMed IDs. The total collaboration count reflects the author's overall activity in the co-authorship network, while the number of collaborative publications indicates the strength of their most frequent collaboration.

Table S4. Statistics of Centroid Distances for Six Clusters

Cluster	Mean of CentroidDist	SD of CentroidDist	Median of CentroidDist	Min of CentroidDist	Max of CentroidDist
**Cluster 1(Noise)**	1.657	0.410	1.685	0.872	2.450
**Cluster 2**	0.623	0.272	0.511	0.067	1.153
**Cluster 3**	0.367	0.150	0.379	0.150	0.567
**Cluster 4**	0.504	0.250	0.603	0.173	0.829
**Cluster 5**	0.471	0.190	0.474	0.175	0.876
**Cluster 6**	0.558	0.217	0.603	0.202	1.018

Centroid Distance is Euclidean distance between a keyword's (UMAP1, UMAP2) coordinates and the centroid of its assigned cluster (CentroidX, CentroidY) in the UMAP embedding space. In short, it represents the distance from a keyword to the centroid of its cluster. A larger distance indicates a more fragmented cluster.

Table S5. Cross-Cluster Strength between Thematic Clusters Underlying the Inter-cluster Coupling Heatmap

	Cluster 1 (Noise)	Cluster 2	Cluster 3	Cluster 4	Cluster 5	Cluster 6
**Cluster 1 (Noise)**	0.000	5.295	5.429	10.649	2.557	5.601
**Cluster 2**	5.295	0.000	2.835	7.216	3.262	6.967
**Cluster 3**	5.429	2.835	0.000	7.989	1.478	4.409
**Cluster 4**	10.649	7.216	7.989	0.000	6.710	10.404
**Cluster 5**	2.557	3.262	1.478	6.710	0.000	3.114
**Cluster 6**	5.601	6.967	4.409	10.404	3.114	0.000

This table represents the coupling matrix between thematic clusters. Cell (i,j) denotes the cross-cluster strength between cluster i and cluster j, defined as the sum of weights for all cross-cluster edges connecting keywords from two distinct thematic clusters. This quantifies the thematic overlap intensity between the two clusters. The matrix is symmetric with a zero main diabolical line, meaning intra-cluster edges are not computed. Cluster 1 (Noise), labeled as the noise category by HDBSCAN, represents a heterogeneous peripheral keyword set. Its relatively high coupling strength with other clusters can be interpreted as a cross-domain convergence effect, rather than necessarily indicating a single cohesive thematic cluster.

Table S6. Full List of Inter-Cluster Pairs Ranked by Cross-Cluster Strength

Cluster pair		Cross-cluster strength
**Cluster a**	**Cluster b**	
Cluster 1 (Noise)	Cluster 4	10.649
Cluster 4	Cluster 6	10.404
Cluster 3	Cluster 4	7.989
Cluster 2	Cluster 4	7.216
Cluster 2	Cluster 6	6.967
Cluster 4	Cluster 5	6.710
Cluster 1 (Noise)	Cluster 6	5.601
Cluster 1 (Noise)	Cluster 3	5.429
Cluster 1 (Noise)	Cluster 2	5.295
Cluster 3	Cluster 6	4.409
Cluster 2	Cluster 5	3.262
Cluster 5	Cluster 6	3.114
Cluster 2	Cluster 3	2.835
Cluster 1 (Noise)	Cluster 5	2.557
Cluster 3	Cluster 5	1.478

Cross-cluster strength is defined as the sum of weights of cross-cluster edges connecting keywords from two distinct thematic clusters, which is used to quantify the degree of overlap between thematic clusters.

Table S7. Full List of Pair-Specific Bridging Keywords and Keyword–Pair Contributions Across All Inter-Cluster Pairs

Cluster pair		Cross-cluster strength	keyword	Source cluster/ Keyword's cluster	Keyword-pair-contribution
Cluster a	Cluster b				
Cluster 1 (Noise)	Cluster 4	10.649	covid-19	Cluster 4	3.052
Cluster 2	Cluster 4	7.216	natural language processing	Cluster 2	3.031
Cluster 3	Cluster 4	7.989	covid-19	Cluster 4	3.013
Cluster 4	Cluster 6	10.404	twitter	Cluster 4	2.724
Cluster 4	Cluster 6	10.404	covid-19	Cluster 4	2.540
Cluster 2	Cluster 6	6.967	natural language processing	Cluster 2	2.476
Cluster 2	Cluster 6	6.967	social media mining	Cluster 2	2.436
Cluster 1 (Noise)	Cluster 3	5.429	pandemics	Cluster 3	2.308
Cluster 4	Cluster 6	10.404	deep learning	Cluster 6	2.271
Cluster 3	Cluster 4	7.989	pandemics	Cluster 3	2.182
Cluster 1 (Noise)	Cluster 4	10.649	public health	Cluster 1 (Noise)	2.104
Cluster 1 (Noise)	Cluster 3	5.429	public health	Cluster 1 (Noise)	2.087
Cluster 2	Cluster 4	7.216	covid-19	Cluster 4	2.081
Cluster 4	Cluster 5	6.710	content analysis	Cluster 5	2.028
Cluster 1 (Noise)	Cluster 2	5.295	machine learning	Cluster 2	2.021
Cluster 1 (Noise)	Cluster 4	10.649	twitter	Cluster 4	2.018
Cluster 3	Cluster 4	7.989	twitter	Cluster 4	1.973
Cluster 1 (Noise)	Cluster 2	5.295	natural language processing	Cluster 2	1.957
Cluster 4	Cluster 5	6.710	covid-19	Cluster 4	1.834
Cluster 4	Cluster 6	10.404	sentiment analysis	Cluster 6	1.650
Cluster 4	Cluster 6	10.404	topic modeling	Cluster 4	1.646
Cluster 3	Cluster 6	4.409	vaccine	Cluster 6	1.645
Cluster 4	Cluster 6	10.404	latent dirichlet allocation	Cluster 6	1.617
Cluster 1 (Noise)	Cluster 6	5.601	sentiment analysis	Cluster 6	1.595
Cluster 4	Cluster 6	10.404	vaccine	Cluster 6	1.563
Cluster 4	Cluster 5	6.710	topic modeling	Cluster 4	1.558
Cluster 3	Cluster 6	4.409	latent dirichlet allocation	Cluster 6	1.553
Cluster 1 (Noise)	Cluster 4	10.649	topic modeling	Cluster 4	1.549
Cluster 2	Cluster 4	7.216	infodemiology	Cluster 4	1.541
Cluster 3	Cluster 4	7.989	attitude	Cluster 3	1.532
Cluster 4	Cluster 5	6.710	twitter	Cluster 4	1.485
Cluster 3	Cluster 4	7.989	tweet	Cluster 4	1.440
Cluster 1 (Noise)	Cluster 4	10.649	coronavirus	Cluster 4	1.429
Cluster 4	Cluster 6	10.404	social networking	Cluster 6	1.420
Cluster 1 (Noise)	Cluster 6	5.601	vaccine	Cluster 6	1.401
Cluster 2	Cluster 6	6.967	deep learning	Cluster 6	1.360
Cluster 2	Cluster 6	6.967	sentiment analysis	Cluster 6	1.359
Cluster 1 (Noise)	Cluster 3	5.429	perception	Cluster 1 (Noise)	1.347
Cluster 2	Cluster 6	6.967	latent dirichlet allocation	Cluster 6	1.288
Cluster 1 (Noise)	Cluster 4	10.649	perception	Cluster 1 (Noise)	1.247
Cluster 1 (Noise)	Cluster 6	5.601	big data	Cluster 1 (Noise)	1.222
Cluster 3	Cluster 6	4.409	vaccination	Cluster 3	1.213
Cluster 3	Cluster 6	4.409	sentiment analysis	Cluster 6	1.210
Cluster 1 (Noise)	Cluster 6	5.601	public health	Cluster 1 (Noise)	1.208
Cluster 2	Cluster 4	7.216	twitter	Cluster 4	1.208
Cluster 3	Cluster 6	4.409	pandemics	Cluster 3	1.178
Cluster 1 (Noise)	Cluster 4	10.649	mental health	Cluster 1 (Noise)	1.170
Cluster 1 (Noise)	Cluster 4	10.649	big data	Cluster 1 (Noise)	1.158
Cluster 3	Cluster 4	7.989	vaccination	Cluster 3	1.149
Cluster 1 (Noise)	Cluster 3	5.429	vaccination	Cluster 3	1.140
Cluster 3	Cluster 4	7.989	sars-cov-2	Cluster 3	1.135
Cluster 2	Cluster 4	7.216	machine learning	Cluster 2	1.135
Cluster 2	Cluster 6	6.967	machine learning	Cluster 2	1.106
Cluster 4	Cluster 5	6.710	infodemiology	Cluster 4	1.097
Cluster 3	Cluster 4	7.989	infodemiology	Cluster 4	1.082
Cluster 1 (Noise)	Cluster 2	5.295	mental health	Cluster 1 (Noise)	1.053
Cluster 2	Cluster 4	7.216	social media mining	Cluster 2	1.036
Cluster 4	Cluster 5	6.710	association rule mining	Cluster 5	1.012
Cluster 4	Cluster 5	6.710	social media analysis	Cluster 5	0.886
Cluster 1 (Noise)	Cluster 2	5.295	social media mining	Cluster 2	0.856
Cluster 4	Cluster 5	6.710	smoking	Cluster 5	0.854
Cluster 3	Cluster 6	4.409	attitude	Cluster 3	0.819
Cluster 3	Cluster 6	4.409	sars-cov-2	Cluster 3	0.805
Cluster 1 (Noise)	Cluster 3	5.429	attitude	Cluster 3	0.803
Cluster 1 (Noise)	Cluster 3	5.429	sars-cov-2	Cluster 3	0.772
Cluster 1 (Noise)	Cluster 2	5.295	big data	Cluster 1 (Noise)	0.770
Cluster 1 (Noise)	Cluster 2	5.295	public health	Cluster 1 (Noise)	0.742
Cluster 1 (Noise)	Cluster 6	5.601	latent dirichlet allocation	Cluster 6	0.737
Cluster 1 (Noise)	Cluster 2	5.295	suicide	Cluster 1 (Noise)	0.727
Cluster 2	Cluster 6	6.967	reddit	Cluster 6	0.727
Cluster 2	Cluster 6	6.967	social networking	Cluster 6	0.711
Cluster 2	Cluster 4	7.216	symptom	Cluster 2	0.703
Cluster 2	Cluster 4	7.216	topic modeling	Cluster 4	0.693
Cluster 1 (Noise)	Cluster 6	5.601	perception	Cluster 1 (Noise)	0.663
Cluster 1 (Noise)	Cluster 2	5.295	depression	Cluster 1 (Noise)	0.645
Cluster 1 (Noise)	Cluster 6	5.601	drug repositioning	Cluster 1 (Noise)	0.588
Cluster 1 (Noise)	Cluster 6	5.601	social networking	Cluster 6	0.433
Cluster 1 (Noise)	Cluster 3	5.429	youtube	Cluster 1 (Noise)	0.420
Cluster 1 (Noise)	Cluster 3	5.429	vaccination hesitancy	Cluster 3	0.406
Cluster 3	Cluster 6	4.409	vaccination hesitancy	Cluster 3	0.393

This table lists the keywords contributing to cross-cluster coupling for all cluster pairs. Cross-cluster strength is defined as the sum of weights of cross-cluster edges connecting keywords from two distinct thematic clusters, which is used to quantify the degree of overlap between thematic clusters. Keyword-pair contribution represents the contribution of this keyword to the cross-cluster coupling strength of this cluster, which is part of the cumulative contribution to cross-cluster edge weights.

Table S8. Top 30 Overall Bridging Keywords and Their Cross-Cluster Strengths

Keyword	Source cluster/ Keyword's cluster	Cross strength
covid-19	Cluster 4	12.520
natural language processing	Cluster 2	10.642
twitter	Cluster 4	9.408
public health	Cluster 1 (Noise)	7.669
content analysis	Cluster 5	7.472
pandemics	Cluster 3	7.035
vaccine	Cluster 6	6.053
machine learning	Cluster 2	6.023
sentiment analysis	Cluster 6	5.814
latent dirichlet allocation	Cluster 6	5.591
infodemiology	Cluster 4	5.481
topic modeling	Cluster 4	5.446
social media mining	Cluster 2	5.274
vaccination	Cluster 3	4.452
deep learning	Cluster 6	4.062
perception	Cluster 1 (Noise)	3.950
tweet	Cluster 4	3.914
attitude	Cluster 3	3.813
big data	Cluster 1 (Noise)	3.555
sars-cov-2	Cluster 3	3.332
mental health	Cluster 1 (Noise)	2.972
coronavirus	Cluster 4	2.636
social networking	Cluster 6	2.563
reddit	Cluster 6	2.380
vaccination hesitancy	Cluster 3	1.809
adolescent	Cluster 5	1.609
thematic analysis	Cluster 1 (Noise)	1.601
health promotion	Cluster 5	1.552
lstm	Cluster 1 (Noise)	1.396
facebook	Cluster 5	1.388

Cross strength is a metric for each keyword, representing the cross-cluster strength at the keyword level. It denotes the sum of all cross-cluster edge weights between that keyword and keywords in other clusters.

Table S9. Sensitivity Analysis of Burst Detection Parameter Grid (Kleinberg model across γ, slice width, and minimum burst length)

gamma	slice_w	min_len	n_bursts	n_keywords_with_burst	level_mean	level_median	level_max	strength_mean	strength_median	strength_max	longest_duration_years	recent_new_bursts	coverage_mean_kw	realism_median_kw	discriminability	jaccard_to_baseline	spearman_to_baseline	mean_score
0.5	1	1	856	23	9.914719626	10	20	19.84579439	20	40	5	352	0.689389006	2	0.097821575	0.973684211	0.698168555	0.83592638
0.5	1	2	856	23	9.914719626	10	20	19.84579439	20	40	5	352	0.689389006	2	0.097821575	0.973684211	0.698168555	0.83592638
0.5	1	3	3	3	2	2	2	8.666666667	8	10	5	0	0.748148148		0.005262762	0.973684211	0.698168555	0.83592638
1	1	1	232	7	9.271551724	9	18	18.57758621	18	36	4	60	0.683400694	1.5	0.136273095	0.973684211	0.698168555	0.83592638
1	1	2	232	7	9.271551724	9	18	18.57758621	18	36	4	60	0.683400694	1.5	0.136273095	0.973684211	0.698168555	0.83592638
1	1	3	2	2	2	2	2	8	8	8	4	1	0.603529412	2.75	0	0.973684211	0.698168555	0.83592638
2	1	1	65	4	8.292307692	8	16	16.8	16	32	5	21	0.641715383	2.017857143	0.27943492	0.973684211	0.698168555	0.83592638
2	1	2	65	4	8.292307692	8	16	16.8	16	32	5	21	0.641715383	2.017857143	0.27943492	0.973684211	0.698168555	0.83592638
2	1	3	3	3	2	2	2	8.666666667	8	10	5	0	0.677325937	2.75	0.005262762	0.973684211	0.698168555	0.83592638
5	1	1	0	0							0	0				0.973684211	0.698168555	0.83592638
5	1	2	0	0							0	0				0.973684211	0.698168555	0.83592638
5	1	3	0	0							0	0				0.973684211	0.698168555	0.83592638
0.5	2	1	967	31	9.877973113	10	20	39.51189245	40	80	4	310	0.773940982	2	0.040426089	0.973684211	0.698168555	0.83592638
0.5	2	2	967	31	9.877973113	10	20	39.51189245	40	80	4	310	0.773940982	2	0.040426089	0.973684211	0.698168555	0.83592638
0.5	2	3	0	0							0	0				0.973684211	0.698168555	0.83592638
1	2	1	310	12	9.274193548	9	18	37.09677419	36	72	4	94	0.73292234	2.4	0.046089497	0.973684211	0.698168555	0.83592638
1	2	2	310	12	9.274193548	9	18	37.09677419	36	72	4	94	0.73292234	2.4	0.046089497	0.973684211	0.698168555	0.83592638
1	2	3	0	0							0	0				0.973684211	0.698168555	0.83592638
2	2	1	129	5	8.759689922	9	17	35.03875969	36	68	4	39	0.755569322	2.4	0.044331169	0.973684211	0.698168555	0.83592638
2	2	2	129	5	8.759689922	9	17	35.03875969	36	68	4	39	0.755569322	2.4	0.044331169	0.973684211	0.698168555	0.83592638
2	2	3	0	0							0	0				0.973684211	0.698168555	0.83592638
5	2	1	0	0							0	0				0.973684211	0.698168555	0.83592638
5	2	2	0	0							0	0				0.973684211	0.698168555	0.83592638
5	2	3	0	0							0	0				0.973684211	0.698168555	0.83592638
**0.5**	**3**	**1**	**1046**	**37**	**9.718929254**	**10**	**19**	**58.31357553**	**60**	**114**	**6**	**152**	**0.8652386**	**1.8**	**0.028139635**	**0.973684211**	**0.698168555**	**0.83592638**
0.5	3	2	1046	37	9.718929254	10	19	58.31357553	60	114	6	152	0.8652386	1.8	0.028139635	0.973684211	0.698168555	0.83592638
0.5	3	3	0	0							0	0				0.973684211	0.698168555	0.83592638
1	3	1	379	17	9.124010554	9	18	54.74406332	54	108	6	45	0.831436749	3.125	0.02625413	0.973684211	0.698168555	0.83592638
1	3	2	379	17	9.124010554	9	18	54.74406332	54	108	6	45	0.831436749	3.125	0.02625413	0.973684211	0.698168555	0.83592638
1	3	3	0	0							0	0				0.973684211	0.698168555	0.83592638
2	3	1	114	7	8.666666667	9	16	52	54	96	6	0	0.77175331	2.625	0.027544291	0.973684211	0.698168555	0.83592638
2	3	2	114	7	8.666666667	9	16	52	54	96	6	0	0.77175331	2.625	0.027544291	0.973684211	0.698168555	0.83592638
2	3	3	0	0							0	0				0.973684211	0.698168555	0.83592638
5	3	1	26	2	8	8	14	48	48	84	6	0	0.74348557	3.922043011	0	0.973684211	0.698168555	0.83592638
5	3	2	26	2	8	8	14	48	48	84	6	0	0.74348557	3.922043011	0	0.973684211	0.698168555	0.83592638
5	3	3	0	0							0	0				0.973684211	0.698168555	0.83592638

The table reports burst detection outcomes across parameter combinations, including burst counts, number of keywords involved, burst strength statistics, coverage, realism, discriminability, and similarity to the baseline configuration (Jaccard and Spearman). The configuration G0.5_W3_L1 achieved the highest coverage (0.865) and strong realism (1.8), while maintaining a large number of bursts (1046) and broad keyword coverage (37 terms), indicating that the results are both comprehensive and plausible. Compared with other parameter settings, this configuration also showed high consistency (Jaccard=0.974; Spearman=0.698), demonstrating robustness and methodological rigor. Therefore, G0.5_W3_L1 was selected as the primary parameter set for the analysis.

Table S10. Internal validation metrics across clustering configurations (UMAP + HDBSCAN with multiple profiles and K values)

Composite score	Profile	Target K	Got K	Got K nonnoise	No. of noise	Noise ratio	Silhouette	Calinski Harabasz	Davies_bouldin
0.981004085	balanced	6	7	6	47	0.602564103	0.50172465	64.84408731	0.574476393
0.587089011	fine	6 with centrality	7	6	17	0.217948718	0.458542577	67.73738006	0.644529181
0.587089011	fine	6 with strengths	7	6	17	0.217948718	0.458542577	67.73738006	0.644529181
**0.576715564**	**fine**	**6**	6	5	19	0.243589744	0.448627692	**84.39072549**	0.730327862
0.245112431	robust	4	4	3	57	0.730769231	0.4825995	20.89563527	0.51583863
0.183604927	fine	5	5	4	27	0.346153846	0.442824791	63.96509015	0.737507783
-0.125161039	robust	5	5	4	63	0.807692308	0.450033024	41.81641074	0.672841489
-0.151887163	robust	4	5	4	63	0.807692308	0.450033024	41.81641074	0.672841489
-0.215315981	balanced	5	5	4	51	0.653846154	0.467014006	33.72002292	0.822394064
-0.245428598	robust	6	5	4	63	0.807692308	0.450033024	41.81641074	0.672841489
-0.33558354	balanced	6	5	4	51	0.653846154	0.467014006	33.72002292	0.822394064
-0.59187112	fine	4	4	3	26	0.333333333	0.401771696	41.15363732	0.747062657
-2.082456601	balanced	4	4	3	50	0.641025641	0.345855805	23.21677016	1.054850367

The table compares clustering results obtained under different profiles (balanced, fine, robust) and target cluster numbers K=4,5,6. Reported metrics include the proportion of noise points, Silhouette coefficient, Calinski–Harabasz (CH) index, Davies–Bouldin (DB) index, and a composite score. The results indicate that fine K=6 achieves the highest CH value (84.39) and a low noise ratio (0.244) while maintaining reasonable Silhouette (0.449) and DB (0.730). Compared to balanced K=6 (Noise=0.603, CH=64.84), the fine K=6 configuration provides greater interpretability and coverage, and was therefore selected as the default clustering solution.
