# Supplementary material for: Thematic Mapping and Evolution of Social Media Mining in Health Research: Hybrid Bibliometric Synthesis
Source: J Med Internet Res. 2026 May 8;28:e86200. doi: 10.2196/86200 (PMC13160668; doi:10.2196/86200)
Supplement: Multimedia Appendix 7 [file jmir-v28-e86200-s007.pdf]

Multimedia Appendix 7. Interpretation and rational for retaining the Hierarchical Density-Based Spatial Clustering of Applications with Noise unassigned noise set (cluster 1: candidate incubator pool of peripheral cross-cutting topics in health-related social media mining)

In this study, we performed unsupervised clustering using HDBSCAN after UMAP dimensionally reduction on a similarity matrix derived from semantic-structural integration. HDBSCAN is a density-based clustering method whose core principle is not to force each point into a specific cluster, but to identify stable dense regions that remain cohesive across various density thresholds. Consequently, when certain keywords either lack sufficient regional density support in the embedding space, fail to achieve significant membership in stable clusters, or fall within transitional boundary regions between multiple clusters, HDBSCAN labels them as unassigned noise sets. These are uniformly denoted by the Computationally Calculated Label −1 (noise) in the output. First, this typically occurs due to weak co-occurrence relationships or broad semantic references among keywords. Examples include cross-thematic general terms, method generalization terms, or words introduced across fields that have not yet formed stable co-occurrence structures. This results in their discrete or scattered distribution within both the graph structure and semantic space. Second, HDBSCAN's parameter-controlled conservatism may also play a role. When the algorithm prioritizes higher density consistency and cluster stability, it tends to preserve boundary or scattered points as −1 to avoid forced inclusion of heterogeneous points into clusters, thereby reducing overall clustering accuracy. Therefore, Computationally Calculated Cluster Label = −1 is to be understood in this paper as an unassigned and peripheral set, rather than a standard thematic cluster with high internal consistency.

Intuitively, the term “noise” can easily be misunderstood as meaningless or even something to be thrown away. However, in density-based clustering like HDBSCAN, the meaning of Cluster Label −1 is more accurately expressed as the set of points that cannot yet be definitively assigned to any stable thematic cluster. It indicates the algorithm's cautious allocation strategy, avoiding forcing dissimilar keywords into clusters at the expense of internal thematic consistency. In other words, Cluster Label −1 is the result of a conservative approach, and the unassigned set does not automatically indicate misclustering. In this research context, we retain this set because it contains keywords that are peripheral yet demonstrate cross-topic connections, or are in an early emerging phase without stable configurations. Deleting it would cause two types of information loss. First, information loss, which would exclude research signals that actually exist but have not yet solidified into stable thematic clusters. Second, interpretive bias, as its removal would forcefully present the network as overly orderly, thereby obscuring the true knowledge structure driven by cross-thematic, interdisciplinary, and time-sensitive event windows.

Since HDBSCAN may sometimes generate “noise clusters (−1)” indicating poor clustering results, their retention requires careful consideration based on evidence such as clustering performance or intra-cluster variance. While we preserve this keyword set under the label “cluster,” to avoid overinterpreting it as a stable thematic cluster, we adopted an empirical and verifiable evaluation approach in this study. We treat Cluster −1 as an unassigned peripheral set and evaluate its merits for retention across multiple dimensions: internal consistency and structural significance, as well as the characteristics of keywords within this set.

First, if Cluster −1 represents mere algorithmic error or random noise, it typically exhibits interpretability deficiency. If these keywords display highly chaotic semantic content, lack relevance to the research context, and fail to form stable connections with other thematic clusters, then retention is unnecessary. In contrast, if it approaches a peripheral yet meaningful heterogeneous set, it may present another pattern. This cluster contains keywords that are indeed dispersed, leading the algorithm to cautiously label them as unassigned. However, it still includes keywords with clear research semantics and exhibits quantifiable connections and interactions with multiple thematic clusters within the network structure. Based on this logic, we do not treat Cluster −1 as a homogeneous thematic cluster equivalent to others, but rather as a peripheral set/cluster requiring additional evidence to support its inclusion. Therefore, this appendix sequentially provides: quantitative evidence of internal dispersion based on centroid distance in UMAP space; network evidence based on cross-cluster coupling interactions; and trend evidence based on temporal signal consistency in burst detection. These demonstrate the need and rationale for retaining this set at both the knowledge structure and interpretive levels.

Starting with the most straightforward spatial consistency, we performed descriptive statistics on the distances (CentroidDist) between each cluster's keywords and their respective cluster centers in the UMAP embedding space. We calculated their mean, standard deviation, median, minimum, and maximum values to measure the spatial compactness and dispersion of each cluster (see **Table S4 in Multimedia Appendix 6**). Results indicate that Cluster 1 exhibits greater dispersion than other clusters (CentroidDist: Mean = 1.657, SD = 0.410, Median = 1.685, Min = 0.872, Max = 2.450). In contrast, the mean CentroidDist values for Clusters 2–6 clustered within the range of 0.367–0.623, with overall lower standard deviations. This indicates that these clusters are more tightly clustered and exhibit greater internal structural consistency within the two-dimensional embedding space. This finding leads to two key conclusions. First, Cluster 1 is indeed more dispersed, meaning its keywords are farther from the cluster centers. This aligns perfectly with HDBSCAN's mechanism for labeling it as “unassigned/noise.” Furthermore, when a group of points cannot form a stable, dense cluster structure in terms of density, the algorithm conservatively assigns it to −1 to avoid misclassification caused by forced categorization. Second, even more importantly, “greater dispersion” does not equate to absolute “meaninglessness.” Within density-based clustering frameworks, −1 more often reflects a “peripheral and heterogeneous” structural pattern. That is, while the similarity among keywords within -1 is insufficient to establish a homogeneous thematic cluster, they may still fulfill significant roles within the overall knowledge structure.

Beyond spatial dispersion evidence, we can observe that several keywords within Cluster 1 possess clear semantic associations with health research. For instance, it includes mental health-related terms like mental

health, depression, and suicide, as well as keywords related to digital health, data, and data processing such as digital health, big data, LSTM, and web scraping. Such terms are typically not domain-specific keywords central to any single thematic cluster. Instead, they act as interdisciplinary keywords that flow across multiple thematic directions. Consequently, within co-occurrence networks and embedding spaces, they tend to present a structural pattern connecting multiple clusters while belonging to no tightly centralized cluster centers. This behavior leads HDBSCAN to cautiously label them as −1.

We further combined the temporal progression of burst detection results (see **Figures 3** and **4**) to conduct a "trend consistency" test on Cluster 1 (unassigned set). If Cluster 1 were merely algorithmic error or random noise, its keywords would typically lack clear temporal development patterns. That is, these keywords would neither exhibit significant burst growth during specific periods nor correlate with known major events or research trends. On the contrary, if Cluster 1 represents a peripheral yet meaningful heterogeneous set, at least some keywords within it should display burst characteristics during specific time windows, reflecting their role as emerging topics or event-driven diffusion. Combining this with our burst results reveals that many keywords related to public health event windows, platform ecosystem changes, or method expansion exhibited clear burst signals during research phases. More importantly, the temporal window logic revealed by burst detection offers additional insight into the existence of Cluster 1. During major public health events like pandemics or periods of rapid research methodology transformation, academic attention experiences phased focus and rapid spread. Some topics rapidly enter core thematic clusters, such as keywords surrounding the COVID-19 information ecosystem. Other topics, however, may grow in a more dispersed, interdisciplinary way. They are repeatedly referenced across different disciplines, methodological approaches, and platform data studies, yet have not yet formed a stable, homogeneously co-occuring cluster with sufficient density. This state of growth without structure manifests precisely as burst signals in burst detection and as items retained in the unassigned set in HDBSCAN. In other words, bursts provide "temporal consistency," while 1 clusters offer "structural informality. When combined, Cluster 1 more reasonably represents an "early aggregation of peripheral topics" or a "cross-domain convergence set within an event window," rather than meaningless random noise. Results from Figures 4 and 5 indicate that several Cluster 1 keywords—such as mental health, public health, big data, and Google Trends—also feature among high-intensity burst keywords. These keywords exhibit distinct burst intervals on both heatmaps and timelines, often occurring in sync with high-activity years across the entire field—such as the pandemic window and its subsequent phases. This suggests they are not entirely random, meaningless data points, but rather research signals systematically activated by specific events and research agendas. More importantly, the presence of bursts does not imply these keywords form a highly homogeneous, stable thematic cluster internally. Instead, it aligns with Cluster 1's structural characteristics, suggesting these words may emerge from diverse subdisciplines, specific research tasks, and platform data contexts. Their co-occurrence density is insufficient to form a stable cluster, yet they are simultaneously projected into the research foreground during critical periods. Based on this, we explicitly define Cluster 1 as an "unassigned and peripheral set of topics." The term "incubator pool" is used solely to indicate its inclusion of potential developmental directions and cross-thematic signals, rather than equating this set directly with a mature or stable thematic cluster.

Moreover, we examined whether Cluster 1 represents a meaningless point set that should be removed from the network structure. We calculated the cross-cluster coupling strength between thematic clusters, which is the weighted sum of all co-occurrence edges between keywords across clusters, to quantify the degree of co-occurrence and interdependence between different clusters in the literature. Results (see **Table S5-S7 in Multimedia Appendix 6**) indicate that although Cluster 1 is more dispersed in the embedding space, it is not isolated at the network level. Instead, it demonstrates clear, quantifiable connections with multiple key clusters. Among these, the coupling strength between Cluster 1 and Cluster 4 is the highest (cross-cluster strength = 10.649), ranking first in strength among all cluster pairs. Additionally, Cluster 1 exhibits moderately high cross-cluster connections with Cluster 6 (5.601), Cluster 3 (5.429), and Cluster 2 (5.295), while its coupling with Cluster 5 is relatively weaker (2.557). In other words, a more reasonable interpretation of Cluster 1 is that it exhibits systematic co-occurrence coupling with multiple key thematic clusters, particularly showing the strongest connections with the application cluster centered around Cluster 4. This phenomenon fits better with the “peripheral heterogeneous set” interpretation. It indicates that Cluster 1 lacks internal homogeneity to form a stable thematic cluster, yet it serves as a cross-thematic interface/interconnection within the overall knowledge structure. This reflects significant cross-domain coupling and conceptual convergence among different research themes during specific periods, especially during major public health event windows.

To better explain that this cross-clustering connection is not random noise but driven by an “explainable keyword mechanism,” we identified pair-specific bridging keywords for highly connected cluster-pairs, specifically which keyword pairs contribute most to the cross-cluster coupling strength between a given pair of clusters. Results (see **Table S7 in Multimedia Appendix 6**) reveal that the strong coupling between Cluster 1 and core clusters does not arise from numerous meaningless scattered edges, but is concentrated around a small number of keywords possessing clear research semantics and high correspondence with disciplinary development. Taking the strongest coupling between Cluster 1 and Cluster 4 as an example, the primary bridging keywords are: covid-19 (contribution = 3.052), public health (2.104), twitter (2.018), topic modeling (1.549), and coronavirus (1.429). These terms are not random fragments but form a shared linguistic framework linking "event topic (COVID-19/coronavirus) — public health context (public health) — Platform Data (twitter) — Analytical Methodology (topic modeling)." This naturally explains why Cluster 1 exhibits the strongest coupling with Cluster 4 during the pandemic window. These keywords are associated with multiple thematic clusters yet struggle to form sufficiently homogeneous and dense structures within any single cluster, making them more likely to be conservatively labeled as tag −1 by HDBSCAN. Similarly, in the Cluster 1–Cluster 6 pairing, bridging keywords include twitter (1.887), sentiment analysis (1.544), covid-19 (1.493), deep learning (1.074), drug-related side effects and adverse reactions (0.967), further suggesting an interpretable cross-domain shared pipeline of “platform terms + task terms + method terms” between these two clusters. More importantly, from the perspective of overall bridging strength (see **Table S8 in Multimedia Appendix 6**), Cluster 1 also contains keywords with high cross-cluster connectivity across the entire network. For instance, “public health” ranks very high in global bridging strength, while “LSTM”, “thematic analysis”, “big data” and “mental health” also demonstrate significant cross-cluster connectivity contributions.

In addition, the results (see **Table 3, Figure 6, Figure S7-S9 in Multimedia Appendix 1**) within the 3D strategic coordinate diagram for these keywords also indicate the recency of the keywords within this cluster, supporting our decision to retain it. Notably, 16 of the 19 keywords in this cluster show a five-year recency rate above 0.5, indicating that their associated publications are concentrated since five years. This temporal concentration supports interpreting Cluster 1 as a peripheral pool of recently active signals rather than meaningless noise. However, given the low frequencies of some terms, we treat “emerging” here as “recently active” and triangulate this interpretation with burst dynamics and cross-cluster coupling evidence.

In summary, this evidence supports our interpretation of Cluster 1. It represents an unassigned and peripheral cross-cutting set containing keywords that frequently serve as “connectors” within specific event windows or interdisciplinary intersections. It may also include emerging directions still in their early stages, which could potentially develop into stable themes independently in the future.
