# Supplementary material for: Thematic Mapping and Evolution of Social Media Mining in Health Research: Hybrid Bibliometric Synthesis
Source: J Med Internet Res. 2026 May 8;28:e86200. doi: 10.2196/86200 (PMC13160668; doi:10.2196/86200)
Supplement: Checklist 2 [file jmir-v28-e86200-s009.pdf]

Checklist 2. PRISMA-S checklist

Section/topic	#	Checklist item	Location(s) Reported
**INFORMATION SOURCES AND METHODS**			
Database name	1	Name each individual database searched, stating the platform for each.	Page 5-6
Multi-database searching	2	If databases were searched simultaneously on a single platform, state the name of the platform, listing all of the databases searched.	Not applicable.<br>PubMed as only one database was searched, therefore, no multi-database searching was processed.
Study registries	3	List any study registries searched.	Not searched.<br>Study registries were not searched because this bibliometric analysis focused on PubMed-indexed published literature, but not a systematic review of interventions or unpublished studies.
Online resources and browsing	4	Describe any online or print source purposefully searched or browsed (e.g., tables of contents, print conference proceedings, web sites), and how this was done.	Not performed.<br>No additional online or print sources were purposefully browse, as the analysis was limited to PubMed-collected journal articles and the data retrieval was fully automated via API.
Citation searching	5	Indicate whether cited references or citing references were examined, and describe any methods used for locating cited/citing references (e.g., browsing reference lists, using a citation index, setting up email alerts for references citing included studies).	Not searched.<br>We did not conduct forward or backward citation searching, because it was not performed as the study aimed to analyze the complete result set from a predefined query.
Contacts	6	Indicate whether additional studies or data were sought by contacting authors, experts, manufacturers, or others.	Not contacted.<br>We did not contact the authors or other expects because only bibliographic metadata from PubMed were analyzed.
Other methods	7	Describe any additional information sources or search methods used.	Not applicable.
**SEARCH STRATEGIES**			
Full search strategies	8	Include the search strategies for each database and information source, copied and pasted exactly as run.	Page 5-6

Limits and restrictions	9	Specify that no limits were used, or describe any limits or restrictions applied to a search (e.g., date or time period, language, study design) and provide justification for their use.	Page 5-6
Search filters	10	Indicate whether published search filters were used (as originally designed or modified), and if so, cite the filter(s) used.	Page 5-6
Prior work	11	Indicate when search strategies from other literature reviews were adapted or reused for a substantive part or all of the search, citing the previous review(s).	Not applicable.<br>The search strategy was developed de novo for this study and prior reviews informed term selection conceptually, but no existing search strings were reused verbatim.
Updates	12	Report the methods used to update the search(es) (e.g., rerunning searches, email alerts).	Not performed.<br>The search was not updated after the final run on July 31, 2025.
Dates of searches	13	For each search strategy, provide the date when the last search occurred.	Page 5-6
**PEER REVIEW**			
Peer review	14	Describe any search peer review process.	Not performed.<br>The search strategy was not externally peer-reviewed; however, it was iteratively tested and refined, with all parameters and outputs logged for reproducibility
**MANAGING RECORDS**			
Total Records	15	Document the total number of records identified from each database and other information sources.	Page 5, Page 8
Deduplication	16	Describe the processes and any software used to deduplicate records from multiple database searches and other information sources.	Not applicable.<br>Because only PubMed was searched.

Rights and permissions: PRISMA-S: An Extension to the PRISMA Statement for Reporting Literature Searches in Systematic Reviews. Rethlefsen ML, Kirtley S, Waffenschmidt S, Ayala AP, Moher D, Page MJ, Koffel JB, PRISMA-S Group. Last updated February 27, 2020.
